# Gene mutant dosage is associated with prognosis and metastatic tropism in 60,000 clinical cancer samples

**DOI:** 10.1038/s41588-026-02666-z

**Published:** 2026-07-31

**Authors:** Nicola Calonaci, Eriseld Krasniqi, Daniel Colic, Stefano Scalera, Giorgia Gandolfi, Salvatore Milite, Konstantin Bräutigam, Andrea Sottoriva, Trevor A. Graham, Leonardo Egidi, Biagio Ricciuti, Marcello Maugeri-Saccà, Giulio Caravagna

**Affiliations:** 1 Department of Mathematics, Informatics and Geosciences, University of Trieste, Trieste, Italy; 2 Phase IV Clinical Studies Unit, IRCCS Regina Elena National Cancer Institute, Rome, Italy; 3 Institute for Research in Biomedicine (IRB Barcelona), The Barcelona Institute for Science and Technology (BIST), Barcelona, Spain; 4 Clinical Trial Center, Biostatistics and Bioinformatics Division, IRCCS Regina Elena National Cancer Institute, Rome, Italy; 5 Computational Biology Research Centre, Human Technopole, Milan, Italy; 6 Centre for Evolution and Cancer, Institute of Cancer Research, London, UK; 7 Institute of Medical Genetics and Pathology, University Hospital Basel, Basel, Switzerland; 8 Department of Economics, Business, Mathematics, and Statistics ‘Bruno de Finetti’, University of Trieste, Trieste, Italy; 9 Lowe Center for Thoracic Oncology, Dana Farber Cancer Institute, Harvard Medical School, Boston, MA USA; 10 Division of Medical Oncology 2, IRCCS Regina Elena National Cancer Institute, Rome, Italy; 11 Area Science Park, Trieste, Italy

**Keywords:** Tumour biomarkers, Data mining

## Abstract

The interplay between somatic mutations and copy number alterations influences tumor evolution and prognosis. These alterations are often treated independently, overlooking gene mutant dosage (GMD)—a key property of their interaction. Here we develop a computational framework that infers mutation copy number and multiplicity from targeted sequencing panels without requiring matched normal samples. We derive GMD for over 500,000 mutations across 60,000 pan-cancer samples. By stratifying more than 20,000 patients according to GMD across multiple genes, we identify 46 tumor-type-specific biomarkers predictive of survival, 13 of which were undetectable using binary mutant/wild-type models, 26 were associated with metastatic spread and 20 predicted metastatic tropism. Our method reveals GMD patterns as independent predictors of disease prognosis, metastatic potential and site-specific dissemination across diverse tumor types. This augmented insight into genomic drivers enhances our understanding of cancer progression and metastasis and holds the potential to substantially enhance biomarker discovery.

## Abstract (web summary)

The authors present INCOMMON, an open-source Bayesian inference tool that determines the multiplicity and copy number of driver mutations from tumor sequencing datasets.

## Main

Cancerogenesis is a complex, multi-step process involving several genetic and epigenetic changes in tumor suppressor genes (TSGs) and oncogenes^1^. Central to this process are somatic mutations and copy number alterations (CNAs), a byproduct of genomic instability, which can confer a proliferative advantage to specific cancer cell subpopulations^2^, leading to the dominance of particular clones^3–6^. Genetic biomarkers derived from such driver mutations^7–9^ have substantially affected our understanding of cancer and its treatment, providing insights into cancer prognosis^10^ and guiding treatment strategies against specific biological vulnerabilities^11–16^. Despite several successes, understanding the prognostic and predictive implications of key somatic mutations remains a challenge^17^, promoting research toward other approaches, such as those that focus on epigenomic alterations^18,19^.

**Fig. 1:**
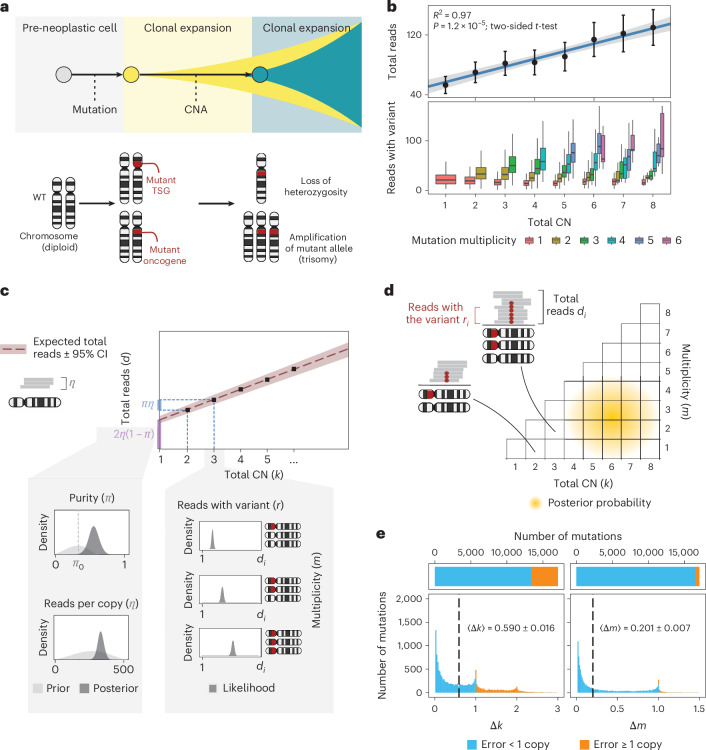
Bayesian inference of mutation multiplicity and CN with INCOMMON. **a**, Clonal expansions from a normal cell that first acquires a mutation in a TSG or an oncogene, then a CNA on the mutant locus, pictured as a loss of the WT allele or a gain of the mutant. **b**, Linear correlation between total read counts and mutation (CN, top), and distribution of reads with the variants (bottom), for increasing mutation multiplicity values, for *n* = 8,339 high-resolution WGS samples available from the PCAWG, HMF and TCGA cohorts. Error bars indicate mean values ± SD. Error bands indicate 95% CIs of the linear regression. Box plots show median (center line), interquartile range (box; 25th–75th percentiles), whiskers extend to 1.5× the interquartile range. **c**, INCOMMON is a Bayesian model that leverages a biology-informed prior and infers the posterior distribution of two sample-level parameters (tumor purity *π* and the rate of reads per chromosome copy *η*) and two mutation-level parameters (total CN *k* and mutation multiplicity *m*) from the number of reads with the variant *r* and the total number of reads *d* for each mutation in the sample. **d**, INCOMMON assigns a posterior probability to any possible configuration of a mutant gene, determined by the co-occurrence of mutations and CN alterations. **e**, Performance of INCOMMON across *n* = 50 cross-validation iterations of random-split cross-validation (70% training, 30% test) using ground truth data from PCAWG^31^ and HMF^32^. The deviation from true values of *k* and *m*, denoted Δ*k* and Δ*m*, was less than one copy on average, occurring in 78.6 ± 0.9% of *k* predictions and 96.4 ± 0.4% of *m* predictions. Chromosome illustrations in panels **a**, **c** and **d** created in BioRender; Calonaci, N. https://biorender.com/5i8eauz (2026).

The current approach to biomarker detection via clinical sequencing lacks an integrative view of the tumor genome. In fact, mutation-derived biomarkers are usually investigated in isolation, and the intricate interplay and epistasis with aneuploidy is potentially overlooked (Fig. 1a). A clear example is the recent discovery of gene mutant dosage (GMD) as a prognostic marker of pancreatic adenocarcinoma^20,21^. GMD reports the number of copies of a mutation in the genome and can be computed only by integrating mutation and CNA data. The derivation of potent biomarkers from clinical sequencing is also complicated by tumor heterogeneity, which drastically reduces sample size when distinct combinations of tumor type, clinical condition and treatment regimen are considered^22^. Moreover, the detection of CNAs from clinical targeted sequencing assays is complicated for experimental reasons, such as the frequent lack of matched normal samples^23–26^.

However, understanding and improving the detection of genetic events that can be exploited in the clinical setting remains crucial, prompting a reevaluation of current tools and data, especially in light of the recent availability of large targeted sequencing clinical cohorts screened for mutations in hundreds of cancer-related genes^27,28^. In this work, we developed INCOMMON, an open-source Bayesian inference tool to determine GMD, as well as mutation CN and multiplicity, and the mutant fraction statistics, for mutations detected in a tumor-only clinical targeted sequencing assay. These statistics naturally reveal the joint effect of mutations and focal aneuploidy on TSG inactivation or oncogene activation, and are readily available for integrated biomarker discovery. To process targeted read count data without matched normal efficiently, INCOMMON is calibrated to use Bayesian priors derived from large-scale whole-genome sequencing (WGS) cohorts. Moreover, to facilitate the usage of orthogonal metrics reported by pathologists in clinical tissue records, INCOMMON models the sample purity probabilistically, resisting potential discrepancies between histological and molecular purity estimates. Compared to state-of-the-art tools like ABSOLUTE^23^, FACETS^24^, PureCN^25^ and CopyWriter^26^, INCOMMON can work without raw data (FASTQ or BAM files or presegmented depth ratios) or matched normal samples, facilitating its clinical implementation while lifting limitations of accessing large controlled-access datasets.

We used INCOMMON to determine GMD patterns over 500,000 mutations detected in 39 principal solid cancer types of over 60,000 clinical samples from the Memorial Sloan Kettering—Metastatic Events and Tropisms (MSK-MET)^29^ and AACR GENIE-DFCI^30^ v.13.0 cohorts. By unraveling GMD statistics from large-scale clinical cohorts, we identified (1) 46 tumor-type-specific biomarkers prognostic of overall survival across 12 tumor types (nine hotspot mutations), (2) 26 biomarkers frequently associated with metastatic spread in 10 tumor types (five hotspot mutations) and (3) 20 biomarkers that predict organotropism in 5 tumor types (two hotspot mutations). Besides confirming established results regarding known TSGs and oncogenes, our method reveals specific GMD patterns as independent predictors of patient prognosis, metastatic propensity and organ-specific metastatic spread across a wide range of solid tumors.

## Results

### The INCOMMON mutation CN caller

We developed INCOMMON (inference of copy number (CN) and mutation multiplicity for oncology), a Bayesian model that infers, for a mutation, (1) the total CN at the mutant locus and (2) the mutation multiplicity (that is, the number of DNA copies that harbor the mutation). This information provides the allele-specific configuration of the mutant locus and the GMD. INCOMMON takes input read counts data for *n* mutations *X* = {*x*_1_,…, *x*_*n*_} to develop the joint likelihood

1 $$p(X{\rm{| }}\Theta)=\mathop{\Pi}\limits_{{x_i}\in {\rm{X}}}p({x}_{i}{\rm{| }}\Theta )=\mathop{\Pi}\limits_{{x_i}\in {X}}p({d}_{i}{\rm{| }}\Theta )p({r}_{i}{\rm{| }}{d}_{i},\Theta)$$

For every mutation *x*_*i*_ = 〈${r}_{i},{d}_{i}$〉, we used the number of reads *r*_*i*_ with the alternative allele and the total reads *d*_*i*_ (depth of sequencing) to infer two sample-specific parameters, (1) the purity (that is, the fraction of tumor cells in the sample, 0 < *π* ≤ 1) and (2) the rate of reads per chromosome copy (*η* ∈ R^+^), and two mutation-specific parameters, (3) the tumor total CN at the locus (*k*_*i*_ ∈ Z^+^) and (4) the mutation multiplicity (*m*_*i*_ ∈ Z^+^, *m*_*i*_ ≤ *k*_*i*_). All mutations in a sample were modeled jointly, irrespective of their location, borrowing information across multiple loci to infer sample-level parameters and per-locus CNs more accurately. INCOMMON links CN to coverage linearly—the expected number of reads for *k* chromosome copies is *kη*—following the observation of a significant linear correlation (*R*^2^ ≥ 90% with *P* < 0.05; Fig. 1b) in 8,339 samples from the Pan-Cancer Analysis of Whole Genomes^31^ (PCAWG), Hartwig Medical Foundation^32,33^ (HMF) and The Cancer Genome Atlas (TCGA)^34^ cohorts, as well as earlier RNA-based work^35–37^.

INCOMMON links the number of reads to the multiplicity and total CN (Fig. 1c), considering tumor–normal admixing:

2 $$p({d}_{i}{\rm{| }}\pi ,\eta ,{k}_{i})={\mathrm{Poisson}}({d}_{i}{\rm{| }}\lambda )\qquad\;\lambda =(1-\pi )2\eta +\pi \eta {k}_{i}$$

3 $$p({r}_{i}{\rm{| }}\pi ,{k}_{i},{m}_{i})={\mathrm{binomial}}({r}_{i}{\rm{| }}{d}_{i},\varphi )\qquad\;\varphi =\frac{{m}_{i}\pi }{2\left(1-\pi \right)+{k}_{i}\pi }$$

The sequencing depth ${d}_{i}$ follows a Poisson distribution with an expected number of reads *λ* defined by combining tumor and (diploid) normal readouts. Given the depth, the number of mutant reads ${r}_{i}$ follows a binomial distribution with probability *φ* determined by the mixing of tumor and normal rates^38^. This hierarchical modeling induces overdispersion naturally, avoiding the need for an arbitrary overdispersion parameter. A graphical representation of the model, its priors and likelihood is shown in Supplementary Fig. 1.

**Extended Data Fig. 2:**
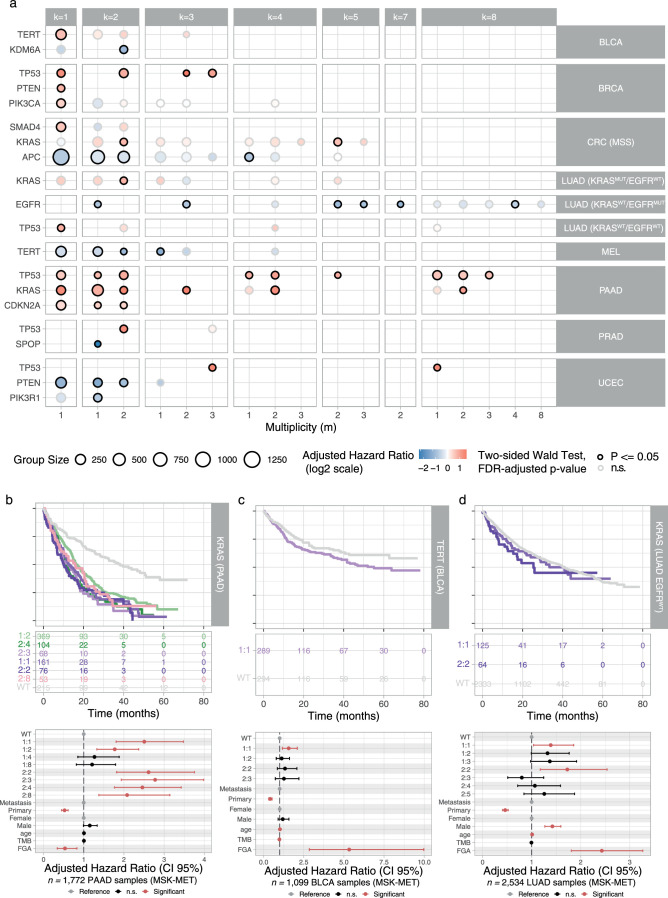
Pan-cancer survival analyses at mutation multiplicity and copy number resolution. **a**. Cox multivariate regression, using sample type (primary vs metastasis), sex, age, TMB and FGA as covariates, at (*k*, *m*) resolution for MSK-MET patients. Survival analysis including Kaplan-Meier estimation and Cox regression hazard ratio estimates for **b**. KRAS mutant pancreatic adenocarcinoma, **c**. TERT mutant bladder cancer, **d**. KRAS mutant, EGFR-WT lung adenocarcinoma. Groups are named by (*k*: *m*), with wild-type (WT) patients as reference. n.s: non-significant.

INCOMMON uses Markov Chain Monte Carlo sampling to estimate a posterior distribution over *m*, *k*, *η* and *π* (Fig. 1d). To leverage the massive amount of public WGS data of human tumors and gain precision with targeted assays, we derived biologically informed prior distributions for (*k*, *m*) configurations from the PCAWG and HMF cohorts (Supplementary Fig. 2; full distribution in Supplementary Table 1). For frequently mutated genes (for example, *PIK3CA* and *TP53* in breast cancer, *KRAS* in pancreatic cancer), these priors were gene-specific and tissue-specific where possible (Supplementary Fig. 3 and Methods). The prior on *η* was derived empirically from the sequencing depth distribution of the datasets analyzed (Supplementary Fig. 4 and Supplementary Table 2). Notably, we also modeled a prior over the input purity *π*, an essential parameter for estimating CNs. To support orthogonal data (for example, from histopathological evaluation) but resist potential errors in the input estimate, we centered a broad prior around the purity measurements provided with each sample (the 95% confidence interval (CI) lying within ±10–15% of the estimate). INCOMMON uses posterior predictive checks (PPCs) (Methods) to monitor the deviation between observed values and inferred posterior distributions. This information is used to annotate rare events (for example, unexpectedly high CNs or multiplicities) and flag poor model fits before assembling all data in a detailed report of the analysis (Extended Data Fig. 2). Example INCOMMON reports for MSK-MET samples, including some for extreme cases with just a single mutation, are shown in Supplementary Figs. 5–8 and discussed in detail in Supplementary Note 1.

Using extensive cross-validation (Methods) across combined PCAWG and HMF data (*n* = 6,492 samples, 46 tumor types; Fig. 1e and Supplementary Fig. 9a), INCOMMON showed low error rates, with 78.6 ± 0.9% of *k* predictions and 96.4 ± 0.4% of *m* predictions within plus or minus one copy of the true value, and strong consistency across folds, supporting the reliability of its estimates for downstream analyses. Comparison with FACETS calls derived from raw data unseen by INCOMMON and available for a subset of MSK-MET^39^ showed low deviations for CN and tumor purity estimates (Supplementary Fig. 9b–d). INCOMMON demonstrated high computational efficiency, requiring a median of 42.0 ± 4.2 s and 90.607 ± 0.003 MB of random access memory to analyze samples containing ten mutations (median of five mutations per sample in MSK-MET). Runtime scaled linearly with input size, supporting its application in large-scale genomic pipelines (Supplementary Table 3).

### Prognostic effect of GMD across 20,000 cancers

**Extended Data Fig. 3:**
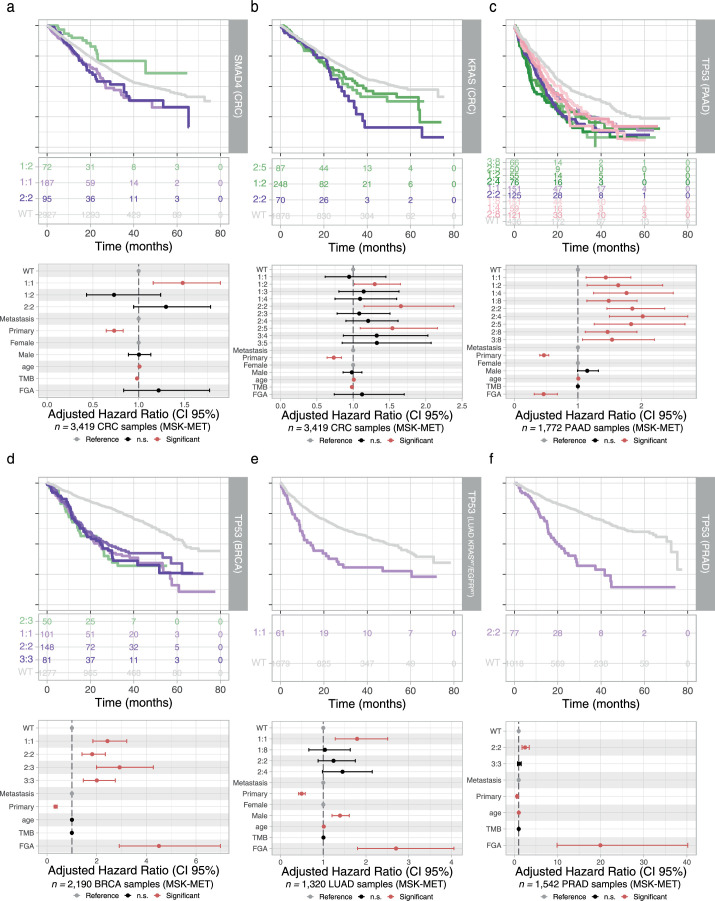
Survival analysis of MSK-MET samples at full allelic resolution. Overall survival analysis including Kaplan-Meier estimation and Cox multivariate regression, using sample type (primary vs metastasis), sex, age, TMB and FGA as covariates, at (*k*, *m*) resolution for MSK-MET patients with **a**. SMAD4 mutant colorectal cancer, **b**. KRAS mutant colorectal cancer, **c**. TP53 mutant pancreatic adenocarcinoma, **d**. TP53 mutant breast cancer, **e**. TP53 mutant lung cancer with no KRAS or EGFR mutations, and **f**. TP53 mutant prostate cancer. Groups are named by (*k*: *m*), with wild-type (WT) patients as reference. n.s: non-significant.

**Extended Data Fig. 4:**
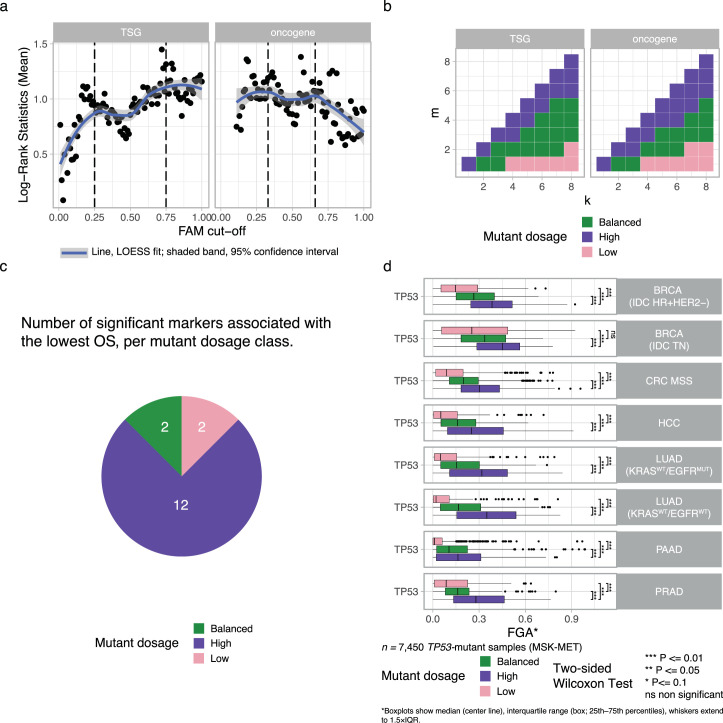
Definition and characterization of gene mutant dosage classes. **a**. Maximally selected rank statistics quantifies the optimal lower and upper cut-points of the fraction of allele with the mutation (FAM) in overall survival (OS) analysis stratifying mutant vs wild-type patients across 84 tumor suppressor genes (TSG, optimal values 25% and 75%), 35 oncogenes (optimal values 33% and 66%) and 18 tumour types. **b**. Classification of mutation total copy number and multiplicity combinations (*k*, *m*) into Low, Balanced and High mutant dosage classes. **c**. Relative fraction of prognostic gene mutant dosage classes across significant markers. **d**. Distribution of the fraction of genome altered (FGA) across gene mutant dosage classes of TP53 in tumour types, compared through two-sided, pairwise Wilcoxon test.

We used INCOMMON on *n* = 21,937 MSK-MET tumors to determine GMD and its prognostic potential against overall survival (OS). Although INCOMMON could jointly model pairs of total and mutant allele configurations (*k, m*) (Extended Data Figs. 2 and 3), stratification at this resolution resulted in small and uneven subgroup sizes, limiting statistical power (Supplementary Fig. 10a). To obtain a more robust approach, comparable with the canonical stratification into mutant (MUT) and wild-type (WT) tumors, we derived the fraction of alleles carrying the mutation (FAM),

4 $${{\rm{FAM}}}_{g,t}=\mathop{\sum }\limits_{k=1}^{{k}_{\max }}\mathop{\sum }\limits_{m=1}^{k}\displaystyle\frac{m}{k}p(m,{k|}{X}_{g,t})$$

from the full INCOMMON posterior distribution *p*(*m*, *k* | *X*_*g*,*t*_). For each gene *g* and tumor type *t*, we used optimized cutoffs to define high, balanced and low GMD classes (Methods and Extended Data Fig. 4), which is consistent with canonical interpretations of allele-specific CN states. High dosage captures loss of the wild-type allele^39–41^ or mutant copy gains^39,40,42,43^, balanced dosage reflects near-equal mutant and wild-type alleles and low dosage reflects wild-type-favored configurations.

**Fig. 2:**
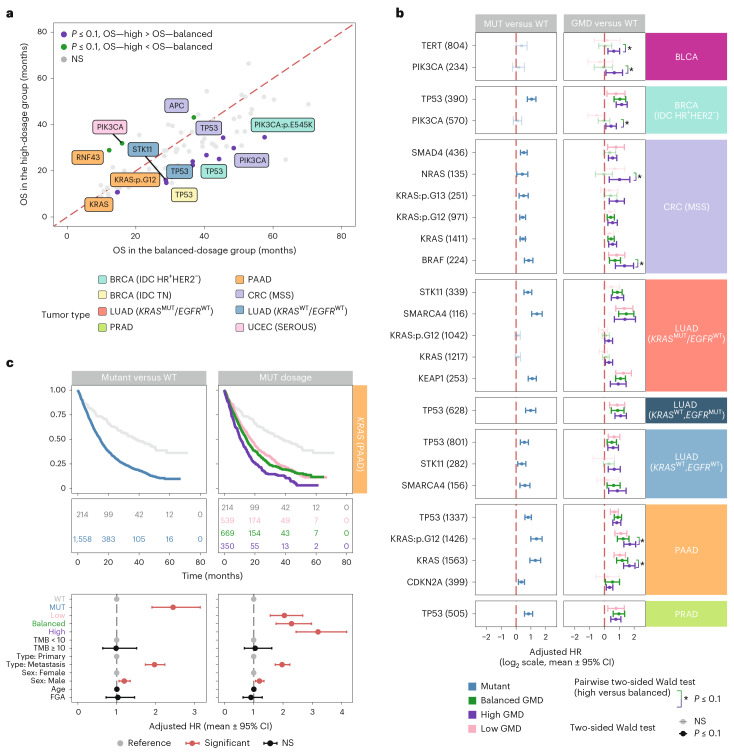
Prognostic effect of GMD across 20,000 cancers. **a**, Comparison of median OS between high and balanced GMD dosage classes, highlighting markers with significant (pairwise two-sided log-rank test *P* ≤ 0.1) OS difference. Tumor subtypes include hormone receptor HR^+^, HER2^−^ and triple-negative (TN) breast cancer (BRCA), lung adenocarcinoma (LUAD) with or without *KRAS* or *EGFR* mutations (superscript MUT and WT indicate whether the respective gene carries a mutation or not), prostate adenocarcinoma (PRAD), PAAD, MSS CRC and serous uterine corpus endometrial carcinoma (UCEC). IDC, invasive ductal carcinoma. **b**, Adjusted hazard ratios (HRs) with 95% CIs from multivariate Cox regression accounting for sex, age, sample type (primary versus metastasis), TMB and FGA, comparing canonical analysis of mutant versus WT tumors (left) with GMD analysis (right). Markers with significant (two-sided Wald test, FDR-adjusted *P* ≤ 0.1) negative prognostic impact associated with high GMD are shown, highlighting those with a significant difference relative to the balanced GMD class. Translucent points and bars indicate non-significant hazard ratio estimates. *n* = 21,937 samples (MSK-MET). BLCA, bladder cancer. **c**, Comparison of Kaplan–Meier curves of OS (top) and adjusted HRs between MUT and WT (left) and GMD stratification (right) for *KRAS* in PAAD. The WT (gray line) is used as the reference group in both cases. For each patient group, the forest plot indicates whether the corresponding regression coefficient is significant (red) or not (black). The regression coefficient for the reference group (gray) is 1. *n* = 1,772 PAAD samples (MSK-MET). NS, not significant.

After establishing the posterior distributions of read count per copy *η*, mutation CN *k* and multiplicity *m* across 493 genes and 50 tumor types (Supplementary Fig. 13), univariate analysis identified significant OS differences across GMD classes in 16 cases (false discovery rate (FDR) *P*_adj_ ≤ 0.1, log-rank test), spanning seven genes and two hotspot mutations across eight tumor types (Fig. 2a). In 75.0% of cases, high dosage was associated with the lowest median OS (16.7% and 8.3% for the balanced and low classes, respectively; Extended Data Fig. 4; full analysis in Supplementary Table 4).

We next evaluated these effects in multivariable Cox models (Methods). Adjustment for tumor mutational burden^44–46^ (TMB) and aneuploidy^47–49^, measured according to the fraction of genome altered (FGA) was essential, as both covariates potentially influence GMD (Supplementary Fig. 14). High GMD was an independent adverse prognostic factor in 24 cases (11 genes and three hotspots) across eight tumor types (Fig. 2b and Supplementary Table 5). Compared with purity-adjusted variant allele frequency (VAF)^11^ (adjusted VAF), GMD provided improved consistency across purity levels in simulations and greater prognostic resolution and precision with MSK-MET data, identifying 26 additional associations supported by prior studies (12 in univariate models; Supplementary Note 2, Supplementary Fig. 10–12 and Supplementary Table 6).

In pancreatic adenocarcinoma (PAAD), high *KRAS* GMD was strongly associated with worse OS compared with both WT and balanced groups (HR = 3.19, CI = 2.45–4.17, *P* < 0.0001; Fig. 2c), especially for G12 mutations, supporting growing evidence of the key prognostic role of *KRAS* GMD^20,21,50,51^. This effect was primarily driven by mutant copy gains (HR = 2.78, *m* = 2 and *k* = 3) and loss of the wild-type allele (HR = 2.51, *m* = *k* = 1), with additional mutant copies further increasing risk (HR = 2.61, *m* = *k* = 2). Notably, the presence of two mutant copies conferred a poor outcome even outside the high-dosage class (HR = 2.46, *m* = 2 and *k* = 4; HR = 2.08, *m* = 2 and *k* = 8), indicating that mutant copy gain can determine disease aggressiveness per se.

**Fig. 3:**
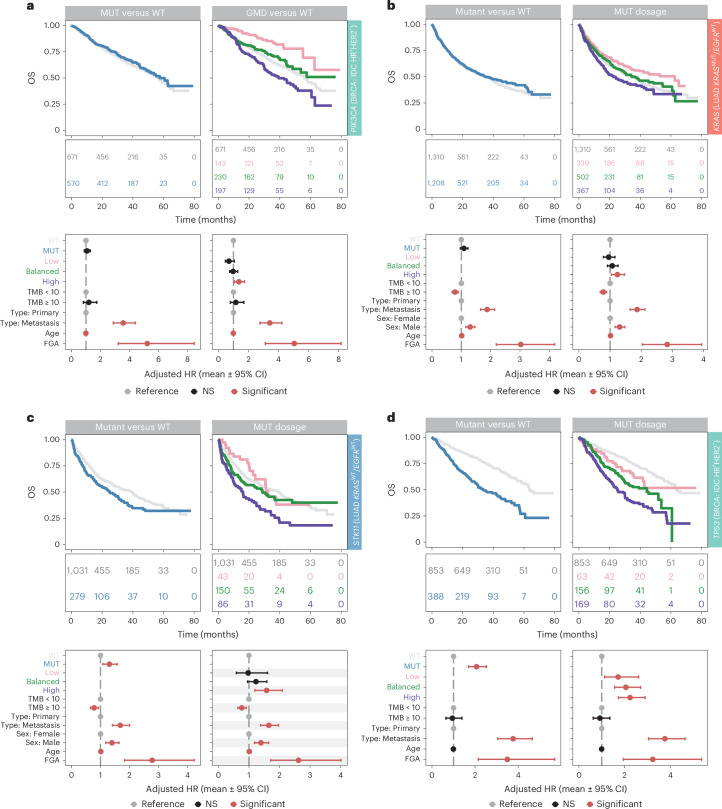
GMD refines survival analysis beyond mutation status. **a**, Comparison of Kaplan–Meier curves, annotated with numbers at risk, of OS (top) and adjusted HR with 95% CIs (bottom) between MUT and WT (left) and GMD stratification (right) for patients with *PIK3CA* mutated and invasive ductal HR^+^HER2^−^ BRCA. *n* = 1,241 (MSK-MET). **b**, Results for mutated *KRAS* and WT *EGFR* LUAD. *n* = 2,518 (MSK-MET) **c**, Results for *STK11* in WT *KRAS* and WT *EGFR* LUAD. *n* = 1,310 (MSK-MET). **d**, Results for *TP53* in invasive ductal HR^+^HER2^−^ BRCA. *n* = 1,241 (MSK-MET). Colours (gray, blue, green, purple and pink) represent the same as in Fig. 2b,c.

In microsatellite-stable (MSS) colorectal cancer (CRC), high GMD *BRAF* (HR = 2.55, CI = 1.67–3.90, *P* = 0.0001) and *NRAS* (HR = 2.02, CI = 1.25–3.27, *P* = 0.0144) mutations were adverse prognostic factors, potentially underappreciated in the prognosis and therapeutic response of *RAS*/*RAF*-driven tumors^12,13^. In bladder cancer, high GMD *TERT* (HR = 1.55, CI = 1.18–2.03, *P* = 0.0140) and, with borderline significance, *PIK3CA* (HR = 1.58, CI = 1.07–2.33, *P* = 0.0945) mutations predicted worse OS, with the *TERT* effect possibly linked to its sensitivity to even modest expression increases^52^, as risk (Extended Data Fig. 3c) was concentrated in states of loss of the wild-type allele with a single mutant copy (HR = 1.56, *m* = *k* = 1). Aligning with and extending recent findings^53,54^, high *PIK3CA* GMD was also prognostic in HR^+^HER2^−^ breast carcinoma (HR = 1.36, CI = 1.07–1.74, *P* = 0.0325; Fig. 3a), driven primarily by loss of the wild-type allele (HR = 1.35, *m* = *k* = 1). Notably, these associations were not detected using canonical binary MUT versus WT stratifications.

In LUAD, the prognostic role of GMD was context-dependent. In WT *EGFR* tumors, high GMD *KRAS* mutations, primarily contributed by *G12*, were associated with worse OS (HR = 1.23, CI = 1.04–1.46, *P* = 0.0384; Fig. 3b), supporting and extending prior findings^55,56^. The adverse effect was primarily driven by loss of the wild-type allele (HR = 1.39, *m* = *k* = 1; HR = 1.73, *m* = *k* = 2; Extended Data Fig. 3d). In wild-type *KRAS* and *EGFR* tumors, only high *STK11* GMD was prognostic (HR = 1.58, CI = 1.19–2.09, *P* = 0.0115; Fig. 3c), whereas in *KRAS* mutant tumors, even balanced GMD was associated with increased risk, clarifying a previously ambiguous pattern^57^.

In *SMAD4* mutant colorectal MSS tumors, only high (HR = 1.46, CI = 1.20–1.78, *P* = 0.0006) and low (HR = 1.71, CI = 1.16–2.54, *P* = 0.0431) GMD were prognostic, highlighting the strong impact of CN alterations compared to plain mutations. The adverse effect was mainly attributable to loss of the wild-type allele with a single mutant copy (HR = 1.48, *m* = *k* = 1), whereas states with mutant copy gains showed greater variability (Extended Data Fig. 3a), probably because of reduced sample size. Conversely, in *KRAS* mutant tumors, particularly with *G12* mutations, only the balanced (HR = 1.36, CI = 1.17–1.58, *P* < 0.0001) and high GMD groups (HR = 1.46, CI = 1.22–1.76, *P* = 0.0003) were associated with increased risk, confirming and extending prior observations^58^. While there was no significant difference between the two groups, gains of the mutant allele with loss of the wild-type (HR = 1.66, *m* = *k* = 2; Extended Data Fig. 3b) and higher ploidy states (HR = 1.54, *m* = 2 and *k* = 5) had a stronger impact than diploid heterozygous states (HR = 1.3, *m* = 1 and *k* = 2).

For the remaining markers, class-level differences across GMD groups were generally not significant despite evidence of prognostic effects. Allelic-level analyses identified multiple individual configurations associated with adverse outcomes, even in the absence of a consistent monotonic relationship with dosage, including in *TP53* mutant PAAD (Extended Data Fig. 3c). In selected breast (Fig. 3d), lung and prostate cancer subtypes, differences across *TP53* GMD classes were significant in univariate but not multivariable models, potentially reflecting confounding by genomic instability^14,59^ (Extended Data Fig. 4 and Supplementary Note 3), resisted only by specific allelic states (Extended Data Fig. 3d–f). This pattern extended to additional genes and tumor types, including *KDM6A*, *PTEN*, *EGFR*, *TERT, CDKN2A*, *SPOP* and *PIK3R1* (Supplementary Note 4 and Supplementary Table 7).

### GMD predicts metastatic propensity and tropism

**Fig. 4:**
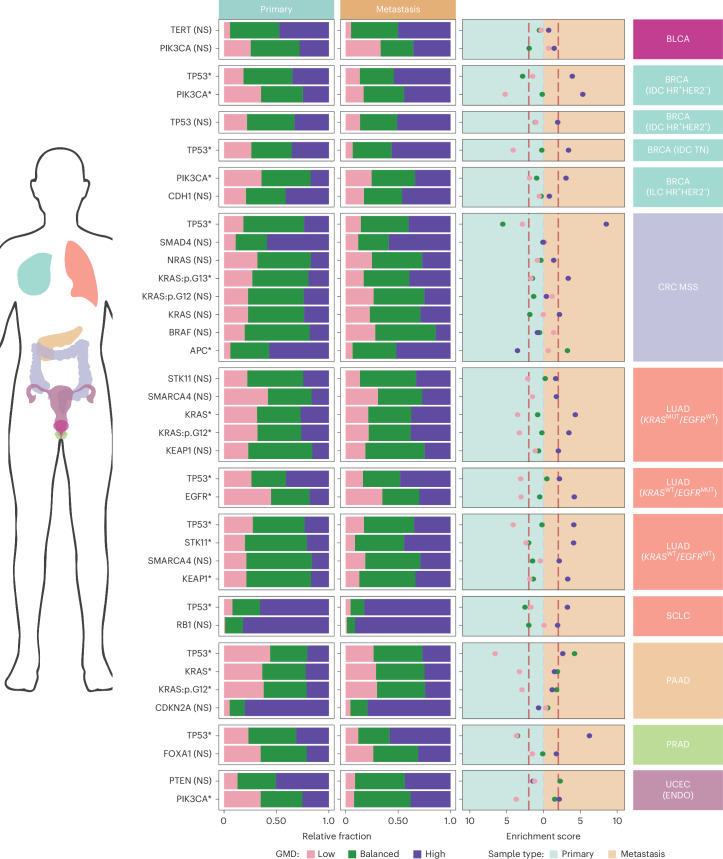
Enrichment of GMD classes in primary tumor versus metastases. Distribution of low, balanced and high GMD classes across 22 genes and 13 tumor subtypes, compared between primary and metastatic tumor samples (left). Right: Enrichment score *r* for each class in either primary or metastatic tumors, evaluated using a two-tailed Fisher’s exact test with a significance level of *α* = 0.1 and using standardized residuals. *P < 0.1 using two-sided Fisher’s test. The vertical dashed lines indicate the thresholds of large effect size (*r* = 2). Tumor subtypes include BLCA, HR^+/−^, HER2^+/−^ and TN BRCA, LUAD with or without *KRAS* or *EGFR* mutations, small-cell lung cancer (SCLC), PAAD, PRAD, MSS CRC and UCEC. Created in BioRender; Calonaci, N. https://biorender.com/5i8eauz (2026).

**Extended Data Fig. 5:**
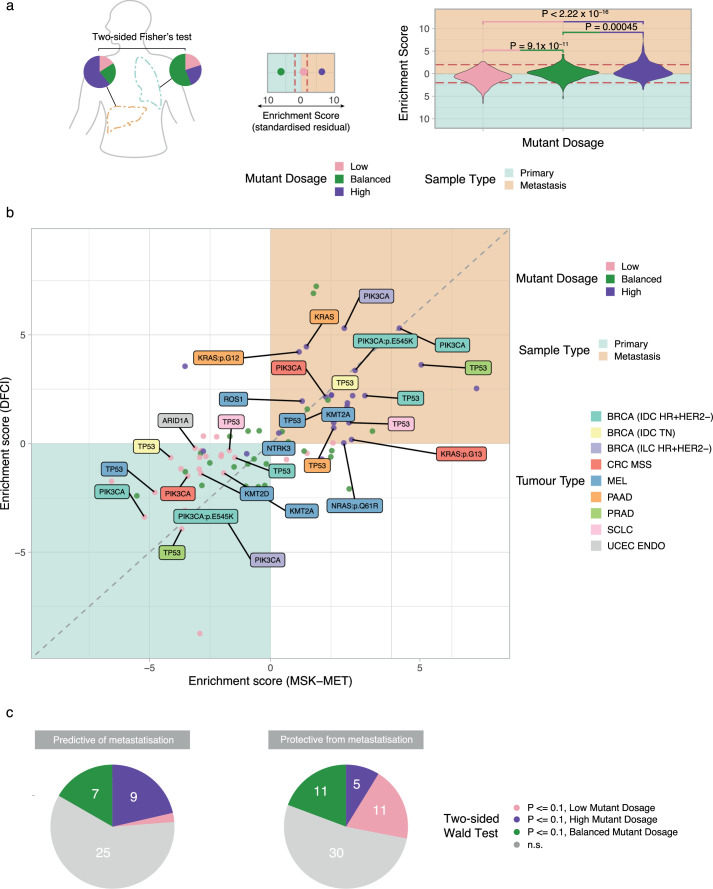
Consistency and distribution of gene mutant dosage enrichment and metastatic associations. **a**. Distribution of enrichment scores in primary tumours vs metastases, defined as the standardised residuals of Fisher’s exact test, across mutant dosage classes. **b**. Comparison of enrichment scores between the MSK-MET and the DFCI cohorts. Significant markers with consistent enrichment scores (distance lower than independent expected noise) across the two cohorts are highlighted. **c**. Distribution of mutant gene dosage classes across markers of increased (left) and decreased (right) metastatic propensity using a univariate logistic model. Panel a created in BioRender; Calonaci, N. https://biorender.com/5i8eauz (2026).

We next examined the impact of GMD on metastatic propensity and organotropism (Methods). Comparing primary (*n* = 13,216) and metastatic (*n* = 8,721) tumors in the MSK-MET cohort revealed a significantly uneven distribution of GMD across disease stages (FDR-adjusted *P* ≤ 0.1, two-tailed Fisher’s exact test), involving 95 genes and 21 hotspots across 27 tumor types (Fig. 4). Although effect sizes were modest (standardized residual *r* = 0.732 for low GMD in primary tumors and *r* = 0.136 and *r* = 0.39 for balanced and high GMD in metastases, respectively), enrichment patterns differed significantly across GMD classes (Extended Data Fig. 5).

Low and high GMD were enriched in primary tumors and metastases, respectively (Supplementary Fig. 15 and Supplementary Table 8). This effect was particularly marked for *TP53* mutant MSS CRC (high-dosage, *r* = 8.52, *P* < 0.0001) and prostate cancer (high-dosage, *r* = 6.23, *P* < 0.0001), and for *PIK3CA* in HR^+^HER2^−^ breast cancers (high-dosage, *r* = 5.33, *P* < 0.0001), which is consistent with the wild-type allele loss requirement for metastatic progression^60^ and with the role of the *PI3K* pathway in chemotherapy resistance^61^. We observed similar patterns consistent with the association of metastatic dissemination with late suppressor biallelic inactivation and amplified *PI3K* pathway activation^32,33,62,63^, *RAS*/*MAPK* pathway regulation and *RTK* signaling^15^, biallelic alteration of chromatin-remodeling genes^63^, *POLE*-driven hypermutation^64^, late *WNT* pathway deregulation^65^, *KEAP1* loss in metastatic niches of LUAD^66^, *p53* pathway inactivation^67^ and early oncogene activation in melanoma^68^ and prostate cancer^20,21,69^ (Supplementary Note 5). We validated the results from the MSK-MET cohort using *n* = 21,462 primary and *n* = 12,049 metastasis samples from the GENIE-DFCI cohort (Extended Data Fig. 5 and Supplementary Fig. 16). A large fraction (89.29%) of the 56 significant markers found in MSK-MET were consistently enriched across both cohorts (Methods), confirming the general trend on *n* = 55,448 total samples.

**Fig. 5:**
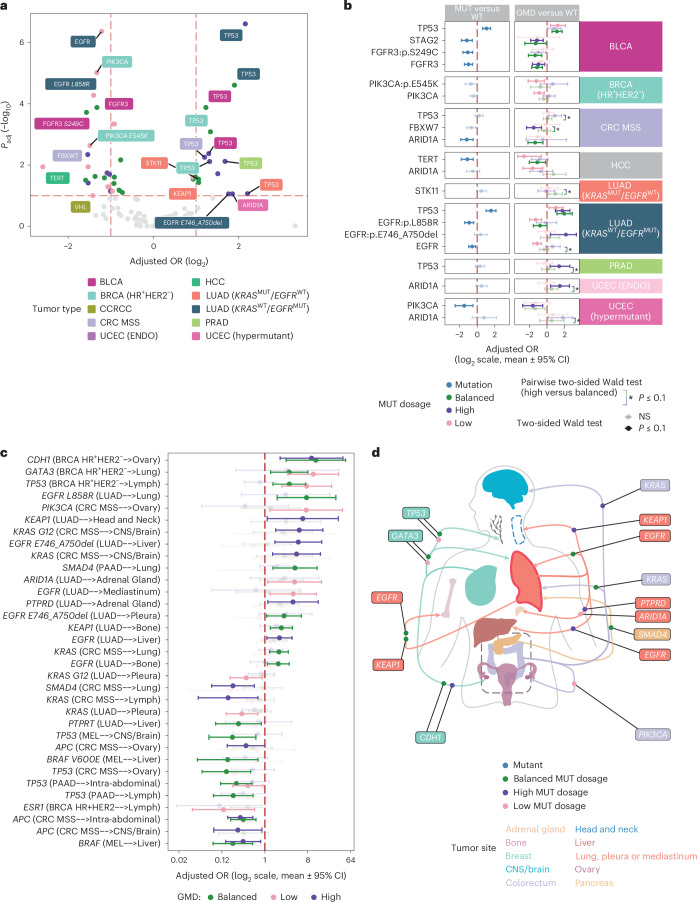
GMD predicts metastatic propensity and organotropism. **a**, ORs of primary tumors with low, high or balanced GMD, giving rise to metastasis. The FDR-adjusted *P* (−log_10_ scale) against the OR (log_2_ scale) is reported. Labels highlighting genes and specific hotspots with a significant impact on metastatic propensity are colored according to the primary tumor type. Values statistically significant (P ≤ 0.1 using a two-sided Wald test) are indicated by pink, purple and green dots for low, high and balanced GMD, respectively. Gray dots indicate values that are not statistically significant. **b**, Adjusted OR from multivariate logistic regression accounting for sex, age, TMB and FGA, comparing canonical analysis of MUT versus WT tumors (left) with GMD analysis (right). Markers with significant (Wald test, FDR-adjusted *P* ≤ 0.1) impact on metastatic propensity associated with GMD are shown, highlighting those with a significant difference between high and balanced GMD classes. *n* = 13,216 primary tumours (MSK-MET). **c**, Adjusted OR of metastatic tropism from primary tumors to other organs associated with GMD. Significant cases are colored. n = 13,216 primary tumours (MSK-MET) **d**, Organotropic patterns associated with GMD, represented by arrows starting from the primary site and pointing to the metastatic site. Dots are colored according to the GMD class associated with the organotropic pattern, while labels highlight significant genes and specific hotspots, and are colored accordingly to the primary tumor type. Tumor subtypes include BLCA, clear-cell renal cell carcinoma, hepatocellular carcinoma (HCC), HR^+/−^, HER2^+/−^ and TN breast cancer, LUAD with or without *KRAS* or *EGFR* mutations, SCLC, PAAD, PRAD, MSS CRC, endometrial (ENDO) and hypermutant UCEC. CNS, central nervous system. Panel **d** created in BioRender; Calonaci, N. https://biorender.com/5i8eauz (2026).

We next quantified the impact of GMD on metastatic propensity. Following 12 genes and four hotspots detected in the univariate analysis (Fig. 5a and Extended Data Fig. 5), our estimates of the adjusted odds ratios (ORs) for GMD classes relative to the WT groups revealed 17 significant associations in multivariable models (FDR-adjusted *P* ≤ 0.1, Wald test; Methods) across eight tumor types (Fig. 5b, Supplementary Fig. 17 and Supplementary Table 9).

High GMD increased the metastatic propensity in 11.8% of the significant cases (Extended Data Fig. 5), including *EGFR* (OR = 4.42, CI = 1.31–27.5, *P* = 0.0884)—especially the exon 19 deletions, E746 A750del—and *TP53* (OR = 3.34, CI = 1.94–5.98, *P* = 0.0001) mutations in WT *KRAS* LUAD, *TP53* in prostate cancer (OR = 2.59, CI = 1.27–6.00, *P* = 0.0450) and *ARID1A* in endometrial cancer (OR = 2.73, CI = 1.31–5.83, *P* = 0.0476), supporting a model in which stronger oncogenic signaling or complete suppressor inactivation are required for invasion and adaptation to distant microenvironments^33,63,70,71^. High GMD alterations were also associated with reduced metastatic propensity for *FGFR3*, *STAG2*, *FBXW7* and *PIK3CA*, suggesting that increased pathway activation or total inactivation may constrain dissemination or reflect less aggressive disease biology, at least in specific contexts^72,73^. Low GMD more consistently showed protective effects, with significant cases including *TERT*, *ARID1A*, *PIK3CA* and *EGFR* (Supplementary Note 6). These findings offered supplementary evidence that partial pathway activation or incomplete loss of suppressor function may be insufficient to induce metastatic competence^52,63^.

Finally, we analyzed organ-specific patterns of metastasis^29^ using multinomial regression (Methods, Fig. 5c,d, Supplementary Fig. 18 and Supplementary Table 10). We identified 33 GMD-associated patterns, including 20 with strong statistical support (FDR-adjusted *P* ≤ 0.05, two-sided Wald test) spanning 14 genes and four hotspots across 11 metastatic sites.

High GMD identified several significant organotropic patterns, including the association of ovarian metastasis with *CDH1* mutations in HR^+^HER2^−^ breast cancer^74^ (OR = 9.58, CI = 2.09–43.8, *P* = 0.00837), which suggested a threshold-like requirement for enhanced epithelial plasticity during peritoneal dissemination, central nervous system (CNS) metastases with *KRAS* mutations (OR = 4.64, CI = 1.46–14.8, *P* = 0.0554), especially for *G12* alterations, indicating that *KRAS*-driven metabolic reprogramming and chromosomal instability might be linked to enhanced colonization of the CNS^75^, and liver involvement with *EGFR* mutations^71^ (OR = 2.03, CI = 1.1–3.74, *P* = 0.0574), which also identified distinct, hotspot-specific patterns for *E746* *A750del* and *L858R* alterations.

High GMD further revealed suppressive organotropic effects, including reduced intra-abdominal metastasis in *APC* mutant MSS CRC, and reduced liver and increased lung metastasis rates for high GMD *BRAF* and *SMAD4* alterations, respectively, which is consistent with context-specific *WNT* and *TGFβ* pathway regulation^75^. Low and balanced GMD showed additional organotropism across genes: *GATA3* was associated with lung metastasis (approximately tenfold enrichment), whereas in HR^+^HER2^−^ breast cancer, *TP53* and *ESR1* mutations had opposite effects, respectively increasing by approximately sevenfold and decreasing the odds of lymphatic dissemination, suggesting two opposite effects of partial impairment of *p53*-mediated differentiation and hormone receptor programs^76^. In MSS CRC, *PIK3CA* low GMD mutations increased the odds of ovarian metastasis (approximately sevenfold increase, borderline significance), suggesting a dosage-specific optimum for *PI3K*-driven peritoneal dissemination^77^. Other genes showed differential organotropism across GMD classes, as in *KEAP1* mutant LUAD, where increasing levels of inactivation were associated with differential metastatic fitness across bone and head-and-neck metastases, which is consistent with oxidative stress response modulation of tissue-specific colonization^78^. Further GMD-stratified patterns across genes and tumor types are provided in Supplementary Note 7.

Overall, our findings demonstrate that metastatic tropism is not simply mutation-specific but is frequently GMD-dependent, with distinct GMD classes within a single gene often predisposing tumors to entirely different organotropic routes.

## Discussion

In this study, we presented INCOMMON, a probabilistic framework to quantify GMD across thousands of human cancers. Unlike existing CN callers^23–26^, INCOMMON operates directly on tumor-only read count data, avoiding reliance on controlled-access raw sequencing data. INCOMMON simultaneously infers mutation CN and multiplicity, and uniquely incorporates informative prior distributions derived from large-scale WGS cohorts, enabling robust and scalable inference across diverse datasets. By jointly modeling mutation CN and multiplicity, our approach allows the measurement of GMD, a statistic that provides a powerful and generalizable stratification of tumor genomes. We demonstrated the high accuracy of our method through extensive cross-validation using well-established WGS data from PCAWG and HMF, confirming the robustness of INCOMMON’s GMD assignments for downstream analyses. By applying this method to approximately 60,000 samples from the MSK-MET and the GENIE-DFCI cohorts, we demonstrated that GMD is a robust biomarker for patient outcome and tumor dissemination across multiple tumor types and genomic contexts.

Our analysis of over 20,000 tumor genomes from MSK-MET uncovered 46 tumor-type-specific biomarkers for which GMD predicted OS, including 38 with strong statistical support and eight with borderline evidence. Notably, 13 of these associations were not detectable using conventional binary models. In most instances (70% of biomarkers), high GMD was associated with significantly poorer prognosis, highlighting a recurrent mechanism of aggressive tumor behavior. This was particularly evident across the *RAS*/*RAF* pathway, where increased GMD in *KRAS*, *NRAS* and *BRAF* consistently tracked with adverse outcomes across multiple tumor types, extending and unifying prior observations from mutation-based and CNA-based analyses^16,20,21,50,51,55,56,58^. These findings reinforce the emerging view that quantitative activation of *RAS*/*RAF* signaling, rather than the mere presence of a mutation, can be a key determinant of disease progression and may bear relevance for the use of *RAS*-targeted therapies^13^. Our results extend prior findings that the number of mutations within single-copy regions or present in multiple copies provides stronger clinical predictive power than global measures of mutational burden or aneuploidy^79^. Importantly, they highlight how GMD sharpens prognostic interpretation beyond coarse burden metrics^29,46^, although such metrics remain independent predictors in specific contexts^10^.

From the analysis of approximately 60,000 unpaired primary and metastatic samples, we found that high GMD was enriched in metastatic tumors, consistently for 95 genes and 21 hotspots across 27 tumor types, with the most prominent effect for *TP53* and *PIK3CA* in breast cancer and CRCs. This enrichment, which we validated across the two independent MSK-MET and GENIE-DFCI datasets, was echoed by elevated metastatic ORs in multivariable models, supporting GMD as a marker of metastatic fitness.

Beyond global metastatic risk, GMD also predicted specific patterns of metastatic spread. We identified 33 tumor-type-specific biomarkers, revealing a new layer of complexity in the molecular logic of organotropism and extending previous findings based on the independent analysis of mutations and CN alterations^29^.

Our findings support a model of oncogenesis in which the timing and allelic configuration of mutations—key drivers of GMD changes—modulate tumor evolution and metastatic trait acquisition. Moving beyond binary mutation classification, our approach integrates mutation type with aneuploidy to yield a more precise risk stratification metric and a mechanistic explanation for previously unresolved clinical heterogeneity. Therefore, incorporating GMD into diagnostic and prognostic models could enhance patient management, especially when treatment decisions depend on gene mutant status.

Despite the breadth of this new analysis, several aspects warrant further development to support broader clinical translation. Reference priors drawn from large WGS cohorts may need to be continuously refined to more closely reflect the histology, treatment exposure and sequencing depth of the clinical populations under study. Inference of GMD in tumors with pronounced intratumoral heterogeneity or very low purity remains challenging, even within our Bayesian framework, and represents an opportunity for methodological advancement. For example, the current model assumes that mutations and CNs are clonal (present in 100% of cancer cells). This approach is suboptimal for detecting ongoing evolutionary processes, which are better elucidated by subclonal deconvolution methods^80^. Unfortunately, these signals are hard to extract from targeted panels and will have to be investigated in future extensions of INCOMMON (Methods). The retrospective nature of the cohorts we analyzed highlights the need for prospective validation of the prognostic and metastatic associations we uncovered.

From a methodological perspective, extending the model to capture WT oncogene amplifications—although rare in primary tumors and undetectable in the present datasets—may provide additional insights into oncogenic activation. The analysis of GMD would also benefit from alternative assays, as transcriptomic and epigenomic readouts may reveal whether high GMD consistently translates into elevated oncogene expression or is tempered by compensatory mechanisms. Dissecting how high-dosage states modulate sensitivity or resistance to targeted agents, immunotherapies and conventional chemotherapy should help turn dosage-based stratification into a practical precision-medicine tool. Matched longitudinal primary or metastatic samples would further refine our understanding of metastatic tropism, while survival data for the GENIE-DFCI cohort would enable validation of our MSK-MET-derived inferences.

In summary, this research advances key aspects of cancer genomics and presents INCOMMON as a transformative tool for interpreting targeted sequencing in the clinic. Our findings lay a strong foundation for future research and clinical translation, contributing both to personalized cancer therapy and to a deeper understanding of the biological complexity underlying genomic alterations. The clinical relevance of these insights underscores their potential to improve patient care.

## Methods

### Inclusion and ethics

All collaborators of this study who have fulfilled the criteria for authorship required by Nature Portfolio journals have been included as authors because their participation was essential for the design and implementation of the study. Roles and responsibilities were agreed upon among collaborators ahead of the research.

### Datasets used for prior construction and performance evaluation

To compute the empirical prior distributions of CN configurations (*k*, *m*), we assembled a cohort of high-resolution WGS data, collecting samples from the PCAWG^31^ dataset (*n* = 2,777 samples, 40 tumor types, median depth 45 and purity 65%), and from the HMF^32^ (*n* = 8,616 samples, 28 tumor types, median depth 96 and purity 55%) dataset. We retrospectively assembled CNA calls from the PCAWG, previously obtained as part of a curated consensus^81^, and from the HMF (previously obtained using PURPLE^2^). We used the resulting dataset of mutations and CNAs as the benchmark dataset B. Importantly, this aggregated dataset includes samples from both primary (from the PCAWG) and metastatic (from the HMF) tumors, ensuring its usefulness in the analysis of datasets containing both sample types, such as the MSK-MET. Additionally, we used data from the TCGA^34^ (1,068 samples) as a validation dataset. As the method is fully data-driven, these empirical priors can be readily updated or refined as additional high-quality datasets become available.

### Bayesian inference of CN and mutation multiplicity using INCOMMON

INCOMMON is a Bayesian framework to infer mutation multiplicity and CN from read count data. The data consists of a set of *n* mutations *X* = {*x*_1_,…, *x*_*n*_} from a tumor sample, with each mutation ${x}_{i}=$ $\langle{r}_{i},{d}_{i}\rangle$ characterized by the number of reads with the variant *r*_*i*_ ∈ Z^+^ and the total number of reads *d*_*i*_ ∈ Z^+^ (sequencing depth). INCOMMON jointly models the purity 0 < *π* ≤ 1 and the rate of reads per chromosome copy (*η* ∈ R^+^) of the sample, and the total CN (*k*_*i*_ ∈ Z^+^) and mutation multiplicity (*m*_*i*_ ∈ Z^+^, *m*_*i*_ ≤ *k*_*i*_) of each mutation in the sample. The joint likelihood of the model is:

5 $$p(X{\rm{| }}\Theta )=\mathop{\Pi}\limits_{{\rm{x\_i}}\in X}p({x}_{i}{\rm{| }}\Theta )=\mathop{\Pi}\limits_{{\rm{x\_i}}\in X}p({d}_{i}{\rm{| }}\Theta )p({r}_{i}{\rm{| }}{d}_{i},\Theta )$$

where Θ is the vector of model parameters Θ = (*π*, *η*, {*k*, *m*}^*n*^).

In INCOMMON, the total number of reads follows a Poisson distribution

6 $$p({d}_{i}{\rm{| }}\pi ,\eta ,{k}_{i})=\mathrm{Poisson}({d}_{i}{\rm{| }}\lambda )\lambda =(1-\pi )2\eta +\pi \eta {k}_{i}$$

The expected value *λ* is contributed by reads from the normal cells present in the sample in a fraction 1 − *π*, and from tumor cells present in a fraction *π*. The expected number of reads from normal cells, assumed to be diploid, is 2*η* across the whole genome. The expected number of reads from tumor cells is *kη*, assumed to be proportional to the unknown total CN.

Given the total read count *d*_*i*_, the number of reads with the variant *r*_*i*_ follows a binomial distribution:

7 $$p({r}_{i}{\rm{| }}\pi ,{k}_{i},{m}_{i})=\mathrm{binomial}({r}_{i}{\rm{| }}{d}_{i},\varphi )\varphi =\frac{m\pi }{2\left(1-\pi \right)+k\pi }$$

The probability of collecting a read with the variant (success probability) *φ* is equal to the VAF expected for a mutation with CN configuration (*k*, *m*), adjusted for the sample purity. INCOMMON embeds prior knowledge of the model parameters Θ using a prior distribution that can be factorized into:

8 $$p(\varTheta )=p(\pi )p(\eta )p(k,m)$$

INCOMMON uses Markov Chain Monte Carlo sampling to estimate the model posterior *p*(Θ | *X*), which would otherwise be intractable analytically.

### Empirical priors

#### CN and mutation multiplicity

A key feature of INCOMMON is the use of biologically informed prior distributions for CN and mutation multiplicity configurations across genes and tumor types. These priors were derived from a training dataset of 4,543 samples (95,896 mutations with available CN states) curated from two large high-resolution WGS cohorts: the PCAWG (1,519 samples) and the HMF (3,024 samples). A subset of samples was held out for model performance evaluation.

We empirically estimated gene-type-specific and tumor-type-specific prior distributions *p*(*k*, *m*) over 36 possible combinations of total CN and mutation multiplicity, with an upper bound of *k*_*max*_ = 8. The prior probabilities were computed based on the observed frequencies of each configuration in the training dataset for each tumor type. To ensure statistical robustness, we required that a gene be mutated in at least 50 samples within a given tumor type to construct a tumor-specific prior; otherwise, a pan-cancer prior was used by aggregating data across all tumor types.

Missing observations were imputed using pseudocounts, estimated through a Gaussian kernel smoothing approach that incorporated CN and mutation multiplicity distances. In particular, given the observed mutation counts *n*_*i*_ for different $\left(k,m\right)$ configurations, we defined a custom distance metric ${d}_{{ij}}=|{k}_{i}-{k}_{j}|+|\displaystyle\frac{{m}_{i}}{{k}_{i}}-\displaystyle\frac{{m}_{j}}{{k}_{j}}|$ and applied a Gaussian kernel ${K}_{{ij}}=\exp (-\displaystyle\frac{{d}_{{ij}}^{2}}{2{\sigma}^{2}})$ to weigh the observations. We then computed the pseudocounts as ${\widetilde{n}}_{i}={\sum }_{j}{K}_{{ij}}{n}_{j}+1$, where we used 1 as the base pseudocount, ensuring numerical stability. To preserve the overall mutation burden, we applied a normalization step to get the final pseudocount: ${\widetilde{n}}_{i}\leftarrow {\widetilde{n}}_{i}\frac{10+{n}_{i}}{{\sum }_{j}{n}_{j}}$.

#### Tumor purity

The inference of CN and multiplicity with INCOMMON critically relies on sample purity, a piece of information that is usually accessible as provided together with genomic data. However, the available purity estimates are often pathology-based; thus, they are potentially incorrect or differing from molecular estimates. The full Bayesian formulation of INCOMMON allows using an informative, yet broad, prior on sample purity *π*, defined starting from the available estimate *π*_0_. INCOMMON uses a Beta distribution over purity:

9 $$p(\pi )={Beta}({\alpha }_{\pi },{\beta }_{\pi })$$

For each sample, we chose specific values of the shape parameter *α*_*π*_ such that the mean of the distribution was equal to the available estimate *π*_0_, and the expected variance was fixed to *σ*^2^, that is:

10 $${\alpha }_{\pi }={\pi }_{0}\left[\frac{{\pi }_{0}\left(1-{\pi }_{0}\right)}{{\sigma }_{\pi }^{2}}-1\right]$$

11 $${\beta }_{\pi }=\left(1-{\pi }_{0}\right)\left[\frac{{\pi }_{0}\left(1-{\pi }_{0}\right)}{{\sigma }_{\pi }^{2}}-1\right]$$

We used ${\sigma }_{\pi }^{2}=0.05$ for all the datasets we analyzed. In this way, INCOMMON accounts for uncertainty in purity estimation of around 10%. Potentially, there can be cases in which the input purity estimate is not supported by the inference of INCOMMON, despite the allowed uncertainty. We controlled for these extreme cases through PPCs (for an example, see Extended Data Fig. 2).

#### Read count rate per chromosome copy

For the read count rate per chromosome copy η, we estimated the prior distribution from the data in empirical Bayes fashion. In principle, in the following equation, the expected total number of reads from a mutation site with CN $k{\prime}$ is $E({d}_{i}{|k}={k}^{{\prime} })=(1-\pi )2\eta +\pi \eta k{\prime}$. Thus, an empirical estimator of $\widetilde{\eta }$ of the per-copy rate can be obtained from the mean total number of reads coming from all the mutations with $k=k{\prime}$ as $\widetilde{\eta }=\frac{ < d{ > }_{{k}_{i}=k{\prime} }}{2(1-\pi )+k{\prime} \pi }$. As we do not know the CN of mutations in the sample a priori, the best we can do is to use the expected value with respect to the prior probability $p(k)={\sum }_{m=1}^{k}p(k,m)$. Thus, we obtained the estimator:

12 $${\widetilde{\eta}}=\displaystyle\frac{1}{n}\mathop{\sum}\limits_{i=1}^{n}\mathop{\sum }\limits_{k^{\prime} }\displaystyle\frac{{d}_{i}p(k^{\prime} )}{2(1-\pi )+k^{\prime} \pi }\,\,$$

We then used a Gamma prior distribution over η

13 $$p(\eta )=\mathrm{Gamma}({\alpha }_{\eta },{\beta }_{\eta })$$

in which we chose the values of the shape parameters such that the mean is equal to our empirical estimation $\widetilde{\eta }$ and the variance is equal to the empirical variance $\mathrm{Var}(\widetilde{\eta })$ across the dataset:

14 $${\alpha }_{\eta }=\frac{{\widetilde{\eta }}^{2}}{\mathrm{Var}(\widetilde{\eta })}$$

15 $${\beta }_{\eta }=\frac{\widetilde{\eta }}{\mathrm{Var}(\widetilde{\eta })}\,$$

This prior reflects our prior knowledge over latent CN and multiplicity configurations p(k,m) and adapts it to the observed distribution of sequencing depth in the dataset, constraining the posterior estimates of η to reasonable values. The prior distribution over η for all the analyzed cohorts (MSK-MET, GENIE-DFCI) are reported in Supplementary Fig. 4, along with the corresponding observed distributions of sequencing depth. The use of an informative prior for the estimation of the per-copy count rate is crucial, especially in settings where normal samples are not available (because of questions of data disclosure or in tumor-only assays) so that depth ratios cannot be computed.

### Model performance and comparison with FACETS

We evaluated model performance using *n* = 50 iterations of random-split cross-validation (70% training, 30% test) across combined data from the PCAWG and HMF (*n* = 6,492 samples, 46 tumor types; Fig. 1e and Supplementary Fig. 9a). The model achieved a mean absolute error of Δ*k* = 0.590 ± 0.016 for total CN and Δ*m* = 0.201 ± 0.007 for mutation multiplicity, corresponding to deviations of less than one copy on average (that is, one copy miscalled). In fact, 78.6 ± 0.9% of *k* predictions and 96.4 ± 0.4% of *m* predictions fell within ±1 copy of the true value, underscoring the model’s high predictive accuracy. The narrow variability across folds further highlighted the robustness and consistency of the estimates, supporting their reliability for downstream biological interpretation. We also compared INCOMMON calls with FACETS results for lower, major and total CN, which were publicly available for the MSK-IMPACT cohort, a subset of the whole MSK-MET cohort (Supplementary Fig. 9b,c). While these statistics are lower resolution than mutation multiplicities (unavailable in FACETS), we observed relatively low deviations of 0.738, 1.00 and 1.94, respectively. Regarding tumor purity, the two methods tended to adjust the pathologist’s estimate consistently, with a mean deviation of 9.72% between them (Supplementary Fig. 9d). INCOMMON also demonstrated high computational efficiency, requiring under 12 min and less than 200 MB of random access memory to process samples containing up to 100 mutations (Supplementary Table 7 and Supplementary Fig. 9e). Runtime scaled linearly with input size, supporting practical deployment in large-scale genomic pipelines.

### Fraction of alleles with the mutation

INCOMMON allows for the estimation of mutation CN and multiplicity. In our study, using INCOMMON, we estimated the FAM, representing the relative fraction of MUT alleles of a gene within all cells of the sample. The probabilistic model enables the calculation of the expected value of FAM as:

16 $$E(\mathrm{FAM})=\mathop{\sum}\limits_{k=1}^{{k}_{\max }}\mathop{\sum }\limits_{m=1}^{k}\displaystyle\frac{m}{k}p(m,k|X)$$

where *p*(*m*, *k* | *X*) denotes the joint posterior probability of *k* and *m*, obtained by marginalizing the full posterior over sample purity *π* and read count rate per chromosome copy *η*, $p(m,{k|X})={\int }_{0}^{1}d\pi {\int }_{{R}^{+}}d\eta p(\varTheta {|X})$. Even in cases where the exact configuration of (*k*, *m*) cannot be precisely determined, the estimator in equation (16) leverages the full posterior distribution to provide a robust estimate of their ratio *m*/*k*, weighting contributions from different configurations according to their joint probability.

### Classification based on FAM cutoffs

To determine the optimal thresholds for stratifying the FAM with respect to OS, we applied the maximally selected rank statistics method. Lower and upper cutoffs were searched within the ranges FAM < 0.5 and FAM > 0.5, respectively, and defined as those maximizing separation in survival while accounting for multiple testing. This procedure consistently identified thresholds corresponding to biologically interpretable dosage classes. For each gene *g* and tumor type *t*, we defined high dosage (FAM ≥ 0.75 for TSGs; FAM ≥ 0.66 for oncogenes), low dosage (FAM ≤ 0.25 for TSGs; FAM ≤ 0.33 for oncogenes) and a balanced intermediate class. These cutoffs approximate canonical allele-specific CN states: the high-dosage class includes, for example, deletions of the WT allele leading to loss of heterozygosity, typically occurring in TSGs, and gains of the mutant allele frequently affecting oncogenes, possibly combined as in copy-neutral loss of heterozygosity states; the balanced class includes mostly heterozygous diploid states but also states of high ploidy with a similar number of mutant and wild-type alleles; low dosage reflects mutations after copy gains or post-mutation CN events favoring the WT allele. We required a minimum of 20% of samples per group. Survival models were adjusted for age, sex, TMB, fraction of genome altered and sample type (primary versus metastatic).

### Cancer subtypes

MSK-MET samples were stratified into cancer subtypes using established molecular and clinical classifications derived from curated annotations and genomic features. Analyses were restricted to subtypes with sufficient sample size to ensure statistical robustness. Lung cancers were grouped according to driver mutation status (*KRAS*^MUT^, *EGFR*^MUT^ and WT). Breast cancers were classified into standard molecular subtypes based on receptor status. CRCs were divided into MSS and hypermutant groups. Uterine cancers were partitioned into endometrioid, serous and hypermutant subtypes. All downstream analyses were performed independently within each subtype.

### Posterior predictive checking and Bayesian *P* value calculation

To assess model fit and evaluate potential discrepancies between observed data and posterior predictive distributions, we performed PPCs and computed Bayesian *P* values.

#### Posterior predictive *P* value

For each mutation-specific observed quantity ${y}_{i}$, such as reads with the variant ${r}_{i}$ or total reads ${d}_{i}$, INCOMMON generates S posterior predictive samples $\{{\hat{y}}_{i}^{s}{\}}_{s=1}^{S}$ from the model posterior distribution and evaluates the posterior predictive *P* value as:

17 $${P}_{i}=\displaystyle\frac{1}{S}\mathop{\sum}\limits_{s=1}^{S}{\rm{I}}\left(|{\hat{y}}_{i}^{s}-E[{\hat{y}}_{i}]|\ge |{y}_{i}-E[{\hat{y}}_{i}]|\right)$$

where $E[{\hat{y}}_{i}^{s}]$ is the posterior mean of the replicated observations. The indicator function ${\rm{I}}(\cdot )$ evaluates to 1 if the absolute deviation of the posterior predictive sample from the posterior mean is at least as large as the deviation of the observed data and 0 otherwise. Thus, this *P* value quantifies the extremity of the observed data under the model posterior, providing a measure of the goodness of fit for individual mutations.

In samples with only one or two observed mutations, posterior predictive *P* values for the Poisson depth model may approach 1, as the inferred CN *k* closely reproduces the single observed depth; this reflects the limited data rather than model misfit. INCOMMON remains applicable to such cases: the Bayesian formulation provides natural regularization as the sample-level parameters (purity and per-copy read rate) are informed by their priors, yielding stable and biologically plausible posteriors that appropriately reflect increased uncertainty. Examples of single-mutation cases are shown in Supplementary Fig. 6–8.

#### Bayesian *P* value

At the level of the global (sample-wise) parameter y, such as tumor purity π and read counts per chromosome copy η, INCOMMON estimates Bayesian *P* values by comparing posterior and prior distributions. Given S posterior samples $\{{y}^{\mathrm{post}}{\}}_{s=1}^{S}$ and prior samples $\{{y}^{\mathrm{prior}}{\}}_{s=1}^{S}$, the test statistic is based on deviations from the prior mean $E[{y}^{\mathrm{prior}}]$. INCOMMON computes the test statistic differently depending on the assumed prior distribution:

For a beta prior:

18 $${T}_{s}^{\mathrm{prior}}=|{y}_{s}^{\mathrm{prior}}-E[{y}^{\mathrm{prior}}]|,{T}_{s}^{\mathrm{post}}=|{y}_{s}^{\mathrm{post}}-E[{y}^{\mathrm{prior}}]|$$

For a gamma prior:

19 $${T}^{\mathrm{prior}}=\frac{|{y}^{\mathrm{prior}}-E\left[{y}^{\mathrm{prior}}\right]|}{E\left[{y}^{\mathrm{prior}}\right]},{T}^{\mathrm{post}}=\frac{|{y}^{\mathrm{post}}-E\left[{y}^{\mathrm{prior}}\right]|}{E\left[{y}^{\mathrm{prior}}\right]}$$

Thus, the Bayesian *P* value is computed as the proportion of posterior test statistics that exceed or equal the prior test statistics:

20 $$p=\displaystyle\frac{1}{S}\mathop{\sum }\limits_{s=1}^{S}{\rm{I}}\left({T}_{s}^{\mathrm{post}}\ge {T}_{s}^{\mathrm{prior}}\right)$$

This Bayesian *P* value quantifies the extent to which posterior samples deviate from prior expectations, providing an additional measure of model adequacy.

### Setting an upper bound for total CN *k*

In INCOMMON, the model likelihood is computed over a discrete set of combinations of total CN *k* and multiplicity *m*. While for multiplicity an upper bound is set naturally by the constraint that *m* ≤ *k*, that is, the number of mutant copies cannot exceed the total number of copies, for the total CN, the choice of the upper bound *k*_max_ is arbitrary. To choose a reasonable value of *k*_max_, we used the benchmark dataset B to evaluate the frequency of large (more than four) values of *k* and set a threshold above which the number of mutations is negligible. As shown in Supplementary Fig. 19, the distribution of total CN values significantly varied between wild-type and mutant sites, with the number of sites decreasing more rapidly with *k* for the mutant sites with respect to wild-type. In particular, we found fewer than *n* = 100 mutations per value of *k* for *k* > 8 across the whole benchmark dataset (*n* = 2,375,815 mutations in total). Thus, we set the upper bound to *k*_max_ = 8 for all our analyses.

### Exploratory heuristic to identify potentially subclonal mutations

Since INCOMMON assumes mutations to be clonal (cancer cell fraction = 1), we explored a simple post-hoc heuristic to assess whether its PPCs could flag potentially subclonal variants. Specifically, we considered mutations that failed the PPC for variant-supporting reads and whose observed counts were lower than predicted after accounting for CN and multiplicity. To minimize confounding, we restricted this set to mutations with maximum a posteriori multiplicity *m* = 1 and to samples that passed the PPC for purity, ensuring that apparent deficits could not be explained by inflated multiplicity or inaccurate purity inputs. Eventually, the number of potentially subclonal events per gene–tumor-type combination was extremely small, typically 1–10 mutations, and generally less than 5% of all mutations in the group. Because of such limited sample sizes, and the inability to confidently separate true subclonality from residual technical noise, we concluded that INCOMMON outputs do not currently support clonality-stratified survival and metastatic tropism analyses. Therefore, systematic estimation of cancer cell fraction is reserved for future work.

### Statistics and reproducibility

Statistical analyses were conducted in R v.4.3.2 using the following packages: tidyverse (v.2.0.0), stats (v.4.3.3), survival (v.3.7-0), survminer (v.0.4.9), multcomp (v.1.4-26) and nnet (v.7.3-20). Genomic and clinical data, including, when available, OS time (months) and status (living versus dead), sample type (primary tumor or metastasis), age at sequencing, sex, TMB per megabase, FGA, patient’s status (metastatic or nonmetastatic) and site of metastasis, were obtained from publicly available clinical releases. No statistical method was used to predetermine sample size, which was determined solely by data availability. Survival, enrichment, metastatic propensity and organotropism analyses were conducted using the following covariates: age, sex, TMB, FGA and, where relevant, sample type. OS curves and values were calculated using the univariate Kaplan–Meier estimator, with the two-sided log-rank test used to estimate *P* values. HRs were computed using multivariable Cox proportional hazards models constructed independently for each gene, with the two-sided Wald test used to estimate *P* values. For each tumor type, all genes mutated in fewer than 150 samples were excluded from this analysis; the same threshold was applied to specific recurrent amino acid substitutions (hotspot mutations). Enrichment of GMD classes in primary tumors versus metastases was assessed using a two-sided exact Fisher’s test to evaluate deviation from independence under the null hypothesis. An enrichment score was computed from the standardized residuals of the contingency table, which measures the deviation of observed from expected counts, normalized by the standard error. Metastatic propensity was evaluated using generalized linear models with a binomial family and a logit link, with the two-sided Wald test used for statistical significance. To evaluate associations between gene-specific GMD and patient status (organotropism), we used a multinomial log-linear model with metastatic site as the (multivariate) outcome variable and GMD (multivariable) as the predictor. For each gene, the most frequent metastatic site in the WT class was used as the reference category; the two-sided Wald test for statistical significance was performed for each regression coefficient. Wald pairwise comparisons between GMD classes were performed using general linear hypothesis testing. From all fits, we extracted coefficient estimates for all variables and 95% CIs. *P* values for all tests were FDR-adjusted using the Benjamini–Hochberg method, independently for each tumor type, using the number of genes analyzed simultaneously.

### Reporting summary

Further information on research design is available in the Nature Portfolio Reporting Summary linked to this article.

## Online content

Any methods, additional references, Nature Portfolio reporting summaries, source data, extended data, supplementary information, acknowledgements, peer review information; details of author contributions and competing interests; and statements of data and code availability are available at 10.1038/s41588-026-02666-z.

## Supplementary information

Supplementary Information Supplementary Note 1: Interpretation of posterior predictive checks. Supplementary Note 2: Comparison of INCOMMON with purity-adjusted VAF stratification. Supplementary Note 3: Association between TP53 MUT dosage and genomic instability. Supplementary Note 4: Survival analysis. Supplementary Note 5: MUT dosage enrichment in primary tumors versus metastases. Supplementary Note 6: MUT dosage-based metastatic propensity. Supplementary Note 7: MUT dosage-based metastatic organotropism.
Supplementary Figs. 1–19 and Tables 1–11.

Reporting Summary

Supplementary Tables S1-S11 Supplementary Table 1: Priors on (k,m) from WGS data. Supplementary Table 2: Priors on count rate per copy. Supplementary Table 3: Computational efficiency. Supplementary Table 4: Survival analysis (KM). Supplementary Table 5: Survival analysis (Cox). Supplementary Table 6: MUT dosage versus adjusted VAF. Supplementary Table 7: Survival analysis at (k,m) resolution. Supplementary Table 8: MUT dosage enrichment. Supplementary Table 9: Metastatic propensity. Supplementary Table 10: Metastatic tropism. Supplementary Table 11: HMF to INTOGEN conversion

## Figures and Tables

**Extended Data Fig. 1:**
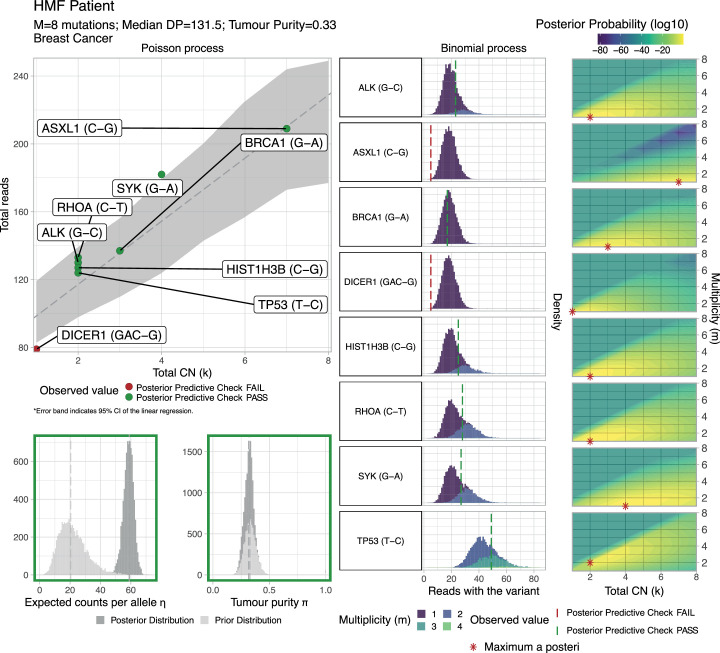
Report of INCOMMON inference for a tumour. Report of the INCOMMON fit of a breast cancer (BRCA) sample from HMF, containing 8 mutations sequenced at median depth 131.5 and with pathologist’s tumour purity estimate of 0.33. In the top left panel, the report displays the Poisson likelihood of total read counts parametrised by mutations’ total copy number (CN). The grey line and ribbon represent the expected number of reads per *k* chromosome copies-*kη*, and the 95% confidence interval of the posterior distribution of total reads per value of *k*. Red dots highlight data failing the posterior predictive check (PPC). The central panel displays the posterior distribution of reads with the variant for all sampled values of multiplicity *m*, with the vertical line indicating the observed value, in red if failing the PPC. The bottom left panels show the comparison between the prior and posterior distributions for the expected count per allele *η* and the tumour purity *π*. The rightmost panels display the posterior distribution over all the possible combinations (*k*, *m*), with the red marker indicating the maximum a posteriori value.

## Data Availability

The PCAWG and The Cancer Genome Atlas data used in this article (mutations with validated matched CNAs) are available from via Zenodo at 10.5281/zenodo.6410935 (ref. ^82^). The MSK MetTropism data used in this article were downloaded from the CBioPortal at https://www.cbioportal.org, under the ‘msk_met_2021’ cohort. The AACR GENIE-DFCI data (v.13.0) were downloaded from Synapse at https://www.synapse.org/ under access nos. syn50678641, syn50678411, syn50678410, syn50678644, syn50678531, syn50678642, syn50678530, syn50678532, syn50678295, syn50678640, syn50678653 and syn50678296. HMF data (segmentation files and purity/ploidy estimates for *n* = 4,496 samples) were obtained from the Hartwig Medical Foundation (version 17/10/2020), using request forms that can be found at www.hartwigmedicalfoundation.nl. The files were generated using the PURPLE pipeline available via GitHub at github.com/hartwigmedical/hmftools/. We then manually converted the Hartwig annotations to match those in ICGC (Supplementary Table 11).

## Code availability

INCOMMON is an open-source R package available via GitHub at https://caravagnalab.github.io/INCOMMON and permanently archived on Zenodo^83^. The tool web page contains RMarkdown vignettes to run analyses, visualize inputs and outputs, and parametrize the tool. INCOMMON is also available as a ShinyApp that can be used to browse the results of the analysis and run similar analyses online. The app is available online at https://ncalonaci.shinyapps.io/incommon/. All analyses presented in this paper, from data gathering to the results of the analysis, are available at Zenodo^84^.
