# Supplementary material for: Gene mutant dosage is associated with prognosis and metastatic tropism in 60,000 clinical cancer samples
Source: Nat Genet. 2026 Jul 31;58(8):1906–17. doi: 10.1038/s41588-026-02666-z (PMC13447112; doi:10.1038/s41588-026-02666-z)
Supplement: Supplementary file 1 [file 41588_2026_2666_MOESM1_ESM.pdf]

nature genetics

Supplementary information https://doi.org/10.1038/s41588-026-02666-z

Gene mutant dosage is associated with prognosis and metastatic tropism in 60,000 clinical cancer samples

In the format provided by the authors and unedited

Supplementary Note 1: Interpretation of posterior predictive checks
1.1 Localised discrepancies
1.2 Global instability
1.3 Inference with one or two mutations: regularisation and uncertainty
Supplementary Note 2: Comparison of INCOMMON with purity-adjusted VAF stratification
2.1 Overlap and differential discoveries
2.2 Biological support for associations uniquely detected by INCOMMON
2.3 Precision of hazard ratio estimates
2.4 Analytical distinction between adjusted VAF and FAM
2.5. Sensitivity to tumour purity misspecification
Supplementary Note 3: Association between TP53 mutant dosage and genomic instability
Supplementary Note 4: Survival Analysis
Supplementary Note 5: Mutant dosage enrichment in primary tumours vs metastases
Supplementary Note 6: Mutant dosage-based metastatic propensity
Supplementary Note 7: Mutant dosage-based metastatic organotropism.
Supplementary Tables
Supplementary Figures
References

Supplementary Note 1: Interpretation of posterior predictive checks

After estimating the full posterior distribution of model parameters Θ = (π, η, k, m), INCOMMON evaluates the consistency between observed data and model-implied distributions for total copy number k, multiplicity m, per-copy read rate η, and tumour purity π using posterior predictive checks (PPCs). Rather than acting as binary acceptance/rejection criteria, PPCs provide structured information about where and how discrepancies arise. Within the MSK-MET cohort, in addition to perfectly fitted samples (N = 14259), we identified the following three major scenarios:

1.1 Localised discrepancies

In N = 6631 samples, although most parameters were well calibrated, we observed minor deviations, including a posterior purity estimate that deviated slightly from the prior mean. In such cases, the discrepancy typically propagated to localised copy-number errors, such as single or a few mutations whose observed variant reads fell in the tail of the posterior predictive distribution (e.g. Supplementary Figure S5).

These situations may reflect locus-specific technical variation, mild subclonality, or prior misspecification, but they do not necessarily indicate model failure at the sample level. INCOMMON explicitly reports these deviations, allowing users to distinguish between isolated events and systemic misfit.

1.2 Global instability

By contrast, in a smaller number of samples (N=1076), PPC failures were coordinated across multiple model parameters (e.g. Supplementary Figure S6). Such discrepancies suggested a structural incompatibility between observed data and the model assumptions, potentially due to inaccurate purity input, unmodelled tumour

heterogeneity, or overly complex copy-number architectures. In these cases, INCOMMON flags the sample as poorly fitted, preventing unreliable estimates from entering downstream analyses.

1.3 Inference with one or two mutations: regularisation and uncertainty

Targeted panel assays frequently yield samples with only one or two detectable mutations. When a single mutation is internally consistent with the joint constraints of the model (e.g. Supplementary Figure S7), INCOMMON can still infer tumour purity, per-copy read rate, total copy number, and multiplicity. The Bayesian formulation was critical in this context: priors on π and η provided regularisation, preventing degenerate solutions and yielding biologically plausible posterior distributions. In such cases, posterior predictive p-values for the Poisson depth model may approach 1 because the inferred copy number closely matches the observed depth. This behaviour reflected limited information rather than overfitting or model misspecification. Importantly, uncertainty was appropriately reflected in broader posterior distributions.

Conversely, when even a single mutation was inconsistent with the joint constraints imposed by sequencing depth and sample purity (e.g. Supplementary Figure S8), PPCs revealed simultaneous deviations in total copy number, multiplicity, and purity. This demonstrated that sparsity alone does not explain poor fit; rather, inconsistency between the observed data and the model's generative structure drives the fit diagnostics.

Thus, INCOMMON can both stabilise inference in underdetermined settings and detect incompatibility in minimal-data scenarios.

Supplementary Note 2: Comparison of INCOMMON with purity-adjusted VAF stratification

To further clarify the added value of INCOMMON relative to tumour purity-adjusted VAF (VAF/π), we performed a systematic comparison of the two approaches across univariate and multivariate Cox regression frameworks, assessed overlap in significant discoveries, evaluated robustness to purity misspecification, and examined biological plausibility of uniquely identified associations.

2.1 Overlap and differential discoveries

Across tumour types, the adjusted VAF approach identified 248 significant associations in univariate models and 121 in multivariate models (FDR < 0.1), compared to 260 and 147, respectively, for INCOMMON mutant dosage. Thus, INCOMMON yielded +12 additional significant classes in univariate analysis and +26 in multivariate analysis.

A comprehensive comparison, including hazard ratios (mean and 95% CI) and FDR-adjusted p-values for both approaches, is provided in Supplementary Table S10. The majority of associations identified by adjusted VAF were also detected by INCOMMON, whereas a non-trivial subset of robust multivariate associations were uniquely recovered by mutant dosage.

2.2 Biological support for associations uniquely detected by INCOMMON

Importantly, several associations uniquely identified by INCOMMON in multivariate analysis are strongly supported by independent biological and clinical evidence. In breast cancer, mutant dosage uniquely recovered associations involving CDH1 (Balanced; HR = 1.56, CI = 1.18–2.06, P = 0.0133), GATA3 (Balanced; HR = 0.708, CI = 0.553–0.906, P = 0.0423), and PIK3CA (Low; HR = 0.628, CI = 0.476–0.827, P = 0.0133), all of which have well-established prognostic or subtype-defining roles[1–3].

In colorectal cancer, INCOMMON uniquely detected robust effects for APC (High class; HR = 0.647, CI = 0.561–0.746, P < 0.0001), BRAF (High class; HR = 2.17, CI = 1.46–3.23, P = 0.00609; Low class; HR = 1.84, CI = 1.30–2.60, P = 0.0180), CTCF (High class; HR = 10.6, CI = 2.64–42.9, P = 0.0230), FAT1 (Intermediate class; HR = 0.469, CI = 0.303–0.727, P = 0.0203), MAP3K1 (High class; HR = 2.82, CI = 1.46–5.45, P = 0.0450), SMAD4 (High class; HR = 1.48, CI = 1.22–1.79, P = 0.00423), and TCF7L2 (Low class; HR = 0.521, CI = 0.361–0.751, P = 0.0180), each previously implicated in tumour progression, chromosomal instability, WNT signalling, or clinical outcome[4–10].

In hepatocellular carcinoma, dosage-based stratification identified prognostic effects for TP53 (Low class; HR = 1.52, CI = 1.14–2.02, P = 0.0142), consistent with established TP53-associated immune and survival models[11].

In lung adenocarcinoma, INCOMMON uniquely detected associations for EGFR (High class; HR = 0.700, CI = 0.577–0.848, P = 0.00411; Low class; HR = 0.647, CI = 0.553–0.756, P < 0.0001), KEAP1 (Low class; HR = 1.76, CI = 1.35–2.29, P = 0.000581), SMARCA4 (High class; HR = 2.00, CI = 1.51–2.66, P < 0.0001; Low class; HR = 1.71, CI = 1.29–2.26, P = 0.00295), and TBX3 (Low class; HR = 0.280, CI = 0.125–0.627, P = 0.0230), all supported by prior evidence linking dosage or mutation context to treatment response and survival[12–15].

In pancreatic adenocarcinoma, mutant dosage stratification highlighted effects for ARID1A (Intermediate class; HR = 1.35, CI = 1.03–1.77, P = 0.0496), CDKN2A (High class; HR = 1.26, CI = 1.11–1.43, P = 0.000695), RNF43 (High class; HR = 0.521, CI = 0.293–0.925, P = 0.0496), and TP53 (Low class; HR = 1.59, CI = 1.32–1.90, P < 0.0001), consistent with previous clinicogenomic studies implicating these drivers in pancreatic tumour progression and outcome[16–18]

Finally, in uterine cancer, additional robust associations were identified for ARID1A (Intermediate class; HR = 0.657, CI = 0.490–0.879, P = 0.0489), BCOR (Low class; HR = 0.116, CI = 0.0285–0.471, P = 0.0343), ERBB3 (High class; HR = 4.14, CI = 1.53–11.2, P = 0.0490), and PTEN (High class; HR = 0.507, CI = 0.381–0.674, P < 0.0001), all consistent with known endometrial tumorigenesis pathways[19–22].

Collectively, these results indicate that the additional associations detected by INCOMMON are not random or unstable findings, but instead correspond to biologically coherent and independently supported potential prognostic relationships.

2.3 Precision of hazard ratio estimates

Beyond differences in the number of significant discoveries, Cox regression analyses demonstrated systematically wider 95% confidence intervals for hazard ratios derived from adjusted VAF stratification (Supplementary Figure S10d), with INCOMMON-based mutant dosage stratification yielding, on average, a 16.93% smaller standard deviation of hazard ratio estimates, on average. Consistently, in both univariate and multivariate models, the variability of hazard ratio estimates was generally higher for adjusted VAF than for INCOMMON (Supplementary Figure S10e). The largest differences were observed in the high-dosage class (FDR-adjusted p-value under t-test $P = 3.68 \times 10^{-16}$ for univariate models; $P = 6.12 \times 10^{-17}$ for multivariate models), where adjusted VAF exhibited

substantially higher median standard errors (0.504 and 0.579 for univariate and multivariate models, respectively) compared with mutant dosage (0.318 and 0.321), corresponding to nearly twofold greater variability. Differences were considerably smaller in the other dosage classes. In the low-dosage class, adjusted VAF showed modestly higher median standard errors (0.335 and 0.343 for univariate and multivariate models, respectively; FDR-adjusted $P = 2.81 \times 10^{-2}$ and $P = 1.79 \times 10^{-2}$) than mutant dosage (0.295 and 0.301). In the intermediate-dosage class, the two approaches produced very similar estimates, with adjusted VAF showing slightly lower median standard errors (0.174 and 0.182; FDR-adjusted $P = 3.05 \times 10^{-5}$ and $P = 3.46 \times 10^{-5}$) than mutant dosage (0.202 and 0.210). Overall, these results indicate that even when both approaches detect significant associations, mutant dosage generally provides more precise and stable estimates of effect size, with the most pronounced improvements observed for high-dosage mutations.

2.4 Analytical distinction between adjusted VAF and FAM

Although purity-adjusted VAF may approximate the fraction of alleles carrying the mutation (FAM) in specific configurations, the two quantities are analytically distinct.

The expected VAF, conditioned on tumor purity π, mutation multiplicity m, and total copy number k, is

$$\varphi = \mathbb{E}[VAF] = \frac{m\pi}{2(1-\pi)+k\pi} \qquad (13)$$

The adjusted VAF equals the FAM only under restricted purity and copy-number configurations. Taking limits over tumor purity yields

$$\lim_{\pi \to 1} \frac{VAF}{\pi} = \frac{m}{k\pi} > \frac{m}{k}; \qquad \lim_{\pi \to 0} \frac{VAF}{\pi} = \frac{m}{2} \neq \frac{m}{k} \quad \forall k \neq 2 \qquad (14)$$

The expected VAF conditional on tumour purity π, mutation multiplicity (m), and total copy number (k) depends jointly on all three parameters. The adjusted VAF equals FAM only under restricted purity and copy-number regimes. In particular, taking limits over tumour purity shows that (i) at high purity, adjusted VAF systematically overestimates FAM (converging to m/k but remaining biased in finite samples), (ii) at low purity, adjusted VAF converges to approximately m/2 only when k = 1 and diverges for other copy-number states.

Thus, adjusted VAF does not provide a consistent estimator of the fraction of mutant alleles across copy-number contexts. In contrast, INCOMMON explicitly models mutation multiplicity and total copy number, directly targeting the biologically interpretable quantity of mutant allele dosage.

2.5. Sensitivity to tumour purity misspecification

We next examined the impact of tumour purity misspecification through simulation. We generated 1,000 tumour samples across varying purity levels and mutation burdens, sampling copy number and mutation multiplicity from prior distributions derived from PCAWG and HMF. Total and variant reads were simulated from Poisson and Binomial distributions, respectively. Simulated purities were then perturbed with errors ranging from 0–100% (median 12.7%, matching TCGA consensus pipeline estimates), enabling direct comparison against ground-truth FAM. Under increasing purity error, the adjusted VAF approach exhibited marked degradation in accuracy, with

FAM error rising to ~50%. In contrast, INCOMMON remained comparatively stable, with error remaining below 20% across the same perturbation range. Quantitatively, adjusted VAF accumulated approximately 10% additional FAM error for every 10% absolute increase in purity error relative to INCOMMON.

This behaviour is consistent with the deterministic structure of adjusted VAF, which divides observed VAF by a fixed purity estimate and therefore directly propagates misspecification error. In contrast, INCOMMON adopts a Bayesian framework that jointly models purity, copy number, and mutation multiplicity, integrating uncertainty into posterior inference and attenuating downstream bias.

Supplementary Note 3: Association between TP53 mutant dosage and genomic instability

In *TP53*-mutant breast, lung and prostate cancers, the prognostic effect of *TP53* mutant dosage was attenuated after adjustment for fraction of genome altered (FGA), suggesting a strong confounding relationship between allele-specific *TP53* status and global aneuploidy. While *TP53* dosage classes were associated with overall survival in univariate analyses in selected breast, lung and prostate cancer subtypes (Fig. 3d), these associations were not robust in multivariable models including FGA and sample type.

To investigate this relationship, we examined the distribution of FGA across *TP53* dosage classes. In all tumour types where *TP53* dosage showed nominal prognostic effects, FGA increased progressively from low to balanced to high dosage groups (Extended Data Fig. 4), with differences reaching statistical significance (Wilcoxon test, $P \leq 0.1$). This pattern is consistent with the established role of *TP53* in maintaining genomic stability, where loss of wild-type function and/or increased mutant allele dosage is associated with widespread chromosomal alterations.

Importantly, allelic-resolution analyses revealed that this association is not uniform across configurations. Although class-level effects were largely explained by FGA, several specific allelic states retained significant hazard ratios after adjustment (Extended Data Fig. 3d–f). These included configurations characterised by loss of the wild-type allele (for example, $m = k = 1$) as well as higher-copy states with multiple mutant alleles, indicating that discrete genomic contexts of *TP53* alteration can identify clinically aggressive subsets beyond the contribution of genome-wide instability.

Supplementary Note 4: Survival Analysis

We first established the distribution of the maximum a posteriori values of read count per copy η, mutation copy number k and multiplicity m, FAM and sample purity across 493 genes and 50 tumour types in the MSK-MET dataset (Supplementary Figure S13). We then investigated the prognostic power of the classes derived from gene mutant dosage. Given the known different prognosis of cancer subtypes, we separately analysed those supported by sufficient sample numbers: mutant/wild-type KRAS and EGFR lung cancers, breast cancer molecular subtypes, micro-satellite stable (MSS) and hypermutant (HM) colorectal cancers, as well as endometrioid, serous and HM uterine cancers. A preliminary univariate analysis showed that the median OS was significantly different (false discovery rate, FDR, adjusted p-value $P \leq 0.1$, Log-rank test) across mutant dosage classes in 16 cases. This covered 7 mutated genes (APC, KRAS, NRAS, PIK3CA, RNF43, STK11, TP53) regardless of the specific

mutation position, and 2 hotspot mutations (KRAS G12 and PIK3CA E545K), for a total of 8 tumour types (Figure 2a). Interestingly, the lowest median OS was associated with the high dosage class in 75.0% of cases, and only rarely with the balanced and low classes (16.7% and 8.3%, respectively; Extended Data Figure 4; full analysis in Supplementary Table S4).

Next, we tested this prognostic effect in a multivariable Cox model for OS adjusted for sex, age at diagnosis, sample type (primary versus metastasis), as well as two well-known biomarkers: the tumour mutation burden (TMB)[23–25] and the overall aneuploidy[26,27], measured as the fraction of genome altered (FGA). Since the association between gene dosage classes and survival may be confounded by higher TMB and FGA, adjusting for these two covariates was essential to understand the actual impact of gene mutant dosage statistics (Supplementary Figure S14).

Our analysis of over 20,000 tumour genomes from the MSK-MET cohort uncovered 46 tumour type–specific biomarkers for which dosage-derived classes were predictive of overall survival, including 38 with strong statistical support (FDR adjusted p-value $P \leq 0.05$, Wald Test), and 8 showing borderline evidence ($0.05 < P \leq 0.1$). Notably, 13 of these associations were not detectable using conventional binary mutant/wild-type models. In most instances (70% of biomarkers), high mutant dosage was associated with significantly poorer prognosis, highlighting mutant dosage increase as a recurrent mechanism of aggressive tumour behaviour. This was particularly evident across the RAS/RAF pathway, where increased mutant dosage in KRAS, NRAS and BRAF consistently tracked with adverse outcomes across multiple tumour types, extending and unifying prior observations from mutation- and CNA-based analyses[16–18,28–31]. These findings reinforce the emerging view that quantitative activation of RAS/RAF signalling – rather than the mere presence of a mutation – can be a key determinant of disease progression and may bear relevance for the use of RAS-targeted therapies[32]. Dosage effects also revealed context-dependent behaviour in genes such as PIK3CA and TERT. The prognostic impact of high PIK3CA dosage in HR+HER2- breast cancer aligned with recent results[2,33], and with the tendency to increase metastatic risk. Conversely, in hypermutated uterine cancers, high dosage PIK3CA mutations were associated with a markedly reduced risk of metastasis and improved survival, possibly identifying a subset of endometrioid-like, estrogen-dependent tumours. In addition, the association of high dosage PIK3CA and TERT mutations with poor outcome in bladder cancer points to a broader relevance of dosage-mediated pathway activation in these tumours. That TERT dosage emerged as a prognostic marker is consistent with the unusually sensitive regulation of telomerase expression, where even modest dosage imbalances may meaningfully influence tumour adaptation[34]. Similarly, dosage-dependent effects in STK11 clarified previously ambiguous prognostic patterns in lung adenocarcinoma[35], revealing distinct interactions with oncogenic background that would remain obscured under standard mutant-versus-wild-type analyses. Finally, while TP53 dosage showed prognostic associations in univariate analyses, multivariate models revealed that much of its apparent effect was absorbed by global aneuploidy measures. The observation that FGA progressively increases from low to balanced to high TP53 dosage suggests a dosage-sensitive relationship between TP53 disruption and genomic instability[36,37], underscoring the need to account for background aneuploidy when evaluating the clinical impact of TP53 alterations.

Our analysis showed that high mutant dosage was a negative prognostic factor in 24 cases (11 genes and 3 hotspots) across 8 tumour types (Figure 2b; full analysis in Supplementary Table S5).
Among these, we searched for scenarios where the high dosage classes were significantly more associated with worse prognosis (higher adjusted hazard ratio, HR) compared to balanced ones. We found this strong trend for KRAS in pancreatic adenocarcinoma (HR = 3.19, CI = 2.45-4.17, P < 0.0001, Figure 2c), including G12 hotspot mutations (HR = 3.27, CI = 2.48-4.30, P < 0.0001), supporting previous evidence that mutant dosage might be a key determinant of poor outcome in KRAS-mutant tumours[16–18,28–31]. When we checked this in individual allelic configurations of (m:k) values (Extended Data Figure 2b), the adverse effect of high KRAS mutant dosage was

primarily driven by mutant copy gains in trisomy (m=2 and k=3, HR = 2.78, CI = 1.93-3.99, P < 0.0001) and by loss of the WT allele (m=k=1, HR = 2.51, CI = 1.81-3.48, P < 0.0001), with additional mutant copies further increasing risk (m=k=2, HR = 2.61, CI = 1.82-3.76, P < 0.0001). The presence of two mutant copies was associated with poor outcome even outside the high-dosage class, including balanced tetrasomy (m=2 and k=4, HR = 2.46, CI = 1.77-3.43, P = 0) and in high ploidy states (m=2 and k=8, HR = 2.08, CI = 1.38-3.14, P = 0.0013), with milder impact in diploid heterozygous states (m=1 and k=2, HR = 1.77, CI = 1.33-2.37, P = 0.0003). These findings indicate that mutant copy gain per se, rather than the specific copy-number configuration, was the principal determinant of more aggressive KRAS-mutant pancreatic tumours. Similarly, high dosage of BRAF (HR = 2.55, CI = 1.67-3.90, P = 0.0001) and NRAS (HR = 2.02, CI = 1.25-3.27, P = 0.0144) mutations in MSS colorectal cancer were adverse prognostic factors, potentially underappreciated in the prognosis and therapeutic response of RAS/RAF-driven tumours[32,38]. High dosage of PIK3CA (HR = 1.58, CI = 1.07-2.33, P = 0.0945) and TERT (HR = 1.55, CI = 1.18-2.03, P = 0.0140) mutations in bladder cancer predicted worse survival, representing, to our knowledge, a novel observation for these genes, even if the statistical significance of the effect for PIK3CA was borderline in adjusted models. The TERT finding was notable, given its unusually sensitive regulation, where even modest expression increase from promoter mutations or copy-number gains could drive tumorigenesis[34]. Allelic configuration-level analyses (Extended Data Figure 2c) indicated that TERT-associated risk was concentrated in states of loss of the WT allele with a single mutant copy (m=k=1, HR = 1.56, CI = 1.16-2.1, P = 0.0176), whereas higher-copy groups were not significant, possibly owing to reduced statistical power. High PIK3CA mutant dosage was also prognostic (HR = 1.36, CI = 1.07-1.74, P = 0.0325) in HR+HER2- invasive ductal breast cancer (Figure 3a), aligning with and extending recent findings(Migliaccio et al. 2022; Correia et al. 2022), contributed almost completely by mutations in single copy combined with loss of WT (m=k=1, HR = 1.35, CI = 1.03-1.76, P = 0.0499) . Notably, PIK3CA and TERT in bladder and breast cancers (Figure 3a) did not emerge as significant markers when using a canonical stratification of mutant versus wildtype patients, highlighting the enhanced resolution provided by gene mutant dosage. A similar result occurred in specific lung cancer subtypes. For instance, high mutant *KRAS* dosage (HR = 1.23, CI = 1.04-1.46, P = 0.0384, Figure 3b) and its *G12* hotspot mutations (HR = 1.22, CI = 1.02-1.46, P = 0.0328) were significantly prognostic in *EGFR*-wildtype adenocarcinomas, supporting and extending prior findings[29,31], although in this case we found only mild difference for the non-prognostic balanced class (HR = 1.06, CI = 0.899-1.26, P = 0.474). In contrast to pancreatic and colorectal cancers, the adverse effect of high mutant dosage in this subtype was predominantly driven by loss of the wild-type allele rather than by additional mutant copy gain (Extended Data Figure 2d), being supported by both m=k=1 (HR = 1.39, CI = 1.04-1.86, P = 0.0927) and, with a stronger effect, m=k=2 (HR = 1.73, CI = 1.18-2.54, P = 0.0378) configurations, whereas double-mutant copy states retaining the wild-type allele were associated with lower and non-significant risks.

The possibility of zooming into a mutant dosage analysis allowed us to detect new prognostic classes which might be obscured in canonical analysis (mutant versus wild-type), increasing our biological understanding of tumour genomes. An exemplary case was *STK11* in lung adenocarcinomas[35]: in *KRAS* and *EGFR* wild-type tumours (Figure 3c), only high *STK11* mutant dosage was associated with worse prognosis (HR = 1.58, CI = 1.19-2.09, P = 0.0115), whereas in *KRAS*-mutant tumours even balanced *STK11* dosage was prognostic. Similarly, in *SMAD4* mutant colorectal MSS tumours, only high (HR = 1.46, CI = 1.20-1.78, P = 0.0006) and low (HR = 1.71, CI = 1.16-2.54, P = 0.0431) *SMAD4* dosage were prognostic, highlighting the strong impact of copy number alterations compared to plain *SMAD4* mutations. The adverse effect of high dosage was mainly attributable to configurations involving loss of the WT allele with a single mutant copy (m=k=1, HR = 1.48, CI = 1.16-1.89, P = 0.0125), whereas states with additional mutant copy gains (for example m=k=2 or higher) showed more variable estimates (Extended Data Figure 3a), likely due to reduced sample size. Conversely, in mutant *KRAS* tumours – particularly those with *G12* hotspot mutations – a low mutant dosage was not prognostic, unlike a balanced (HR = 1.36, CI = 1.17-1.58, P < 0.0001) or high dosage class (HR = 1.46, CI = 1.22-1.76, P = 0.0003), which were associated with

an increased risk of death, confirming and extending prior observations[30]. While mutant dosage did not identify significantly different outcomes, gains of the mutant allele in loss of the WT (2:2, HR = 1.66, CI = 1.15-2.39, P = 0.0263, Extended Data Figure 3b) and higher ploidy states (m=2, k=5 , HR = 1.54, CI = 1.1-2.16, P = 0.039) had again a stronger impact than diploid heterozygous states (m=1, k=2, HR = 1.3, CI = 1.02-1.65, P = 0.0946).
For the remaining markers where the high-dosage class was prognostic, we did not observe significant differences across dosage classes in univariate or multivariate analyses. Allelic-level analyses confirmed the absence of a clear monotonic dosage gradient, yet showed that multiple specific configurations were individually associated with adverse outcomes, for instance, in *TP53*-mutant pancreatic adenocarcinoma (Extended Data Figure 3c), where multiple allelic contexts conferred comparable risk without defining a coherent class-level effect. The only exceptions were *TP53* in certain breast, lung and prostate cancer subtypes (Figure 3d), where mutant dosage was significant in univariate analysis, but lacked robustness in multivariate analysis. In these cases, FGA and sample type accounted for most of the prognostic effect, with FGA progressively increasing from low to balanced to high *TP53* dosage (significant at $P \leq 0.1$, Wilcoxon test, Extended Data Figure 4), suggesting that the relationship between *TP53* alteration and genomic instability[36,39] strengthened with increasing mutant dosage and underscoring the importance of accounting for background aneuploidy when evaluating its prognostic impact. Nevertheless, even in these cases, specific allelic configurations retained significant hazard ratios (Extended Data Figure 3d-f), indicating that discrete *TP53* genomic states can still identify clinically aggressive subsets despite the confounding effect of genome-wide instability.
Finally, in some cases where the aggregated gene mutant dosage classes were not prognostic, we could still retrieve individual allelic configurations associated with significant hazard rations, such as for *KDM6A* in bladder cancer, *PTEN* in breast cancer, *EGFR* in KRAS-WT lung cancers, *TERT* in melanoma, *CDKN2A* in pancreatic adenocarcinoma, *SPOP* in prostate cancer, *TP53*, *PTEN* and *PIK3R1* in uterine endometrial cancer. The complete list of hazard ratio estimates are available in Supplementary Table S11.

Supplementary Note 5: Mutant dosage enrichment in primary tumours vs metastases

We compared the distribution of gene mutant dosage between primary (n = 13,216) and metastatic (n = 8,721) tumour samples from the MSK-MET cohort.
Our analysis revealed an uneven distribution of INCOMMON gene mutant dosage between primary and metastatic tumours from 27 tumour types, 95 genes and 21 hotspot mutations (Figure 4), using a two-tailed Fisher's exact test at significance level α = 0.1, together with a score r from the standardised residuals. Although the median enrichment was small for all classes (r = 0.732 for low mutant dosage in primary tumours, r = 0.136 for balanced dosage and r=0.39 for high dosage in metastases), the distribution of scores was significantly different across classes (Extended Data Figure 5). As a general trend, low mutant dosage was more frequent in primary tumours, whereas high mutant dosage was enriched in metastases (Supplementary Figure S15; full analysis in Supplementary Table S6).
We observed a systematic enrichment of high mutant dosages in metastatic samples and low mutant dosages in primary samples, for both TP53 and PIK3CA in breast and colorectal cancers, consistent with late biallelic inactivation of TSGs and amplified PI3K pathway activation, that might be associated with metastatic competence[40–43]. We found a similar enrichment pattern for TP53 also in hepatocellular carcinomas, in pancreatic and prostate cancer, in melanoma, and in lung cancers together with KRAS, EGFR and STK11, all key regulators of RAS/MAPK or RTK signaling potentially implicated in metastatic dissemination[44], and for PIK3CA in uterine endometrioid cancer along with ARID1A, a chromatin-remodeling gene in which biallelic alteration might be linked to progression[43]. Interestingly, in hypermutant uterine cancers, low dosages of POLE were enriched in primary

samples and high dosages in the metastases, suggesting the potential role of POLE dosage in shaping tumour mutation rates[45].

Additionally, high mutant dosage was enriched in several metastatic contexts: APC in prostate cancer metastases, indicating that WNT pathway deregulation might emerge as a later event[46]; KEAP1 in lung cancer metastases lacking KRAS or EGFR mutations, suggesting that KEAP1 loss may be advantageous in metastatic niches[47]; TP53 in small cell lung cancer metastases, that might be explained by the requirement for complete p53 pathway inactivation for late metastatic progression[48]; and KMT2A in melanoma metastases, where chromatin modification might play a central role in clonal diversification and metastatic fitness. Interestingly, in primary melanoma tumours, low dosages of KMT2D, NTRK3, PREX2, ROS1, TP63 and ZFHX3 were more frequent, a pattern compatible with these alterations acting earlier in tumour initiation rather than during dissemination[49]. Low dosages of ZFHX3 were enriched also in primary prostate cancers[50], and of KRAS in pancreatic cancers, potentially indicating that partial KRAS activation characterises early tumour development, with higher dosages emerging later in metastatic outgrowth[17,18].

Overall, the strongest enrichment score in metastatic samples was for TP53 in MSS colorectal (r = 8.52, P < 0.0001) and prostate cancers (r = 6.23, P < 0.0001), supporting prior observations that WT allele loss is necessary for proliferation toward metastasis[51], and for PIK3CA in HR+HER2- breast cancers (r = 5.33, P < 0.0001), consistent with its role in chemotherapy resistance[52]. We validated our results on the MSK-MET cohort using n = 21,462 primary and n = 12,049 metastasis samples from the GENIE-DFCI cohort (Extended Data Figure 5, Supplementary Figure S16). A large fraction (89.29%) of the 56 significant markers that we found in MSK-MET had a consistent enrichment score between the two cohorts (inter-chort variation lower than intra-cohort, Methods), confirming the general trend of low mutant dosage to be more frequent in primary tumours, and high mutant dosages in metastases across a total of n = 55,448 clinical samples.

Supplementary Note 6: Mutant dosage-based metastatic propensity

To investigate the impact of gene mutant dosage on the metastatic propensity, we calculated the odds ratio (OR) of primary tumours with low, balanced or high mutant gene dosage to metastasise relative to wild-type (WT) tumours. In a preliminary univariate analysis, we found 12 genes and 4 specific hotspots with significant OR across 10 tumour types (Figure 5a). High mutant dosage increased the metastatic propensity in n = 9 cases (20.5% of the significant ones), and decreased it in n = 5 cases (Extended Data Figure 5). Low mutant dosage was primarily associated with reduced metastatic propensity (n = 11 cases), with only one case having a negative impact. The balanced class had, in most cases, a similar trend of the corresponding high or low counterpart, but with a less pronounced effect.

To account for potential confounders, we applied a multivariable logistic regression model incorporating sex, age at sequencing, sample type, TMB and FGA, determining statistical significance using a Wald test with FDR-correction (Methods; Supplementary Figure S17; full analysis in Supplementary Table S7). The impact on metastatic propensity remained significant in 17 cases, including 8 genes and 4 hotspots across 8 tumour types (Figure 5b).

High mutant dosage was the only class significantly associated with higher metastatic propensity in several cases. In KRAS-wildtype lung adenocarcinomas, high-dosage EGFR mutations - particularly exon 19 deletions (E746 A750del) - were associated with a strong, albeit of borderline statistical significance, more than fourfold increase in metastatic propensity (adjusted odds ratio OR = 4.42, CI = 1.31-27.5, adjusted p-value P = 0.0884), suggesting that strong pathway activation might promote proliferation, stress adaptation, and invasive potential; by contrast,

low-dosage mutations conferred reduced metastatic risk, suggesting that partial EGFR activation may suffice for tumor initiation but be insufficient to drive dissemination[53,54]. The effect was clear for TP53 in prostate adenocarcinoma (OR = 2.59, CI = 1.27-6.00, P = 0.0450) and ARID1A in endometrial cancers (OR = 2.73, CI = 1.31-5.83, P = 0.0476), potentially representing double-hit events of these tumour suppressors that could enhance invasive potential[40,43]. Remarkably, these genes would not have emerged as markers of metastatic propensity if analysed without considering mutant dosage, i.e. by only comparing mutant vs WT tumours.

Consistent with results from the univariate analysis, several high-dosage mutant classes were associated with a trend toward reduced metastatic risk (OR < 1). In bladder cancer, high-dosage FGFR3 (OR = 0.511, CI = 0.315-0.834, P = 0.0629) and STAG2 (OR = 0.452, CI = 0.276-0.743, P = 0.0312) mutations halved the risk of metastasis, potentially identifying a category of tumours with luminal papillary phenotype and reduced cellular invasiveness[55–57]. Notably, high dosage FBXW7 mutations in colorectal MSS cancers reduced metastatic risk by one third (OR = 0.350, CI = 0.199-0.644, P = 0.00734), as well as PIK3CA mutations in uterine cancer (OR = 0.327, CI = 0.146-0.711, P = 0.0381), supporting recent findings[58,59].

More commonly, low dosage mutations were associated with reduced metastatic incidence. This was statistically significant for TERT (OR = 0.163, CI = 0.0392-0.635, P = 0.0111) and ARID1A mutations (OR = 0.324, CI = 0.151-0.716, P = 0.0111) in hepatocellular carcinoma, reflecting that partial loss of telomerase activity or chromatin remodelling may be insufficient to confer full metastatic fitness[34,43]. Low dosage PIK3CA mutations (OR = 0.541, CI = 0.363-0.806, P = 0.0177) including the E545K hotspot (OR = 0.432, CI = 0.226-0.811, P = 0.0340) in HR+HER- breast cancers, similarly suggested that moderate pathway activation can support primary tumor growth but limit dissemination[52]. In KRAS-wildtype lung adenocarcinoma, low dosage EGFR mutations (OR = 0.457, CI = 0.329-0.636, P < 0.0001), including the L858R hotspot (OR = 0.406, CI = 0.254-0.654, P = 0.0004) were associated with decreased metastatic propensity, suggesting that partial EGFR signaling might suffice for tumor initiation but be insufficient to drive invasion and adaptation in distant niches[53]. The balanced dosage class showed reduced metastatic propensity in selected cases, including FGFR3 (S249C mutations) in bladder cancer (0.392, CI = 0.232-0.661, P = 0.00271) and ARID1A in MSS colorectal cancer (OR = 0.392, CI = 0.222-0.726, P = 0.0308), indicating that an intermediate level of pathway perturbation may confer limited metastatic advantage[55–57]. Mutant TP53 tumours were generally more prone to metastasise than WT, irrespective of the mutant dosage, as in bladder cancer, KRAS-wildtype lung adenocarcinoma with EGFR mutations, and in MSS colorectal cancer[51]. In the latter case, while an increase in metastatic propensity for the high dosage relative to the balanced state was observed, its statistics remained below significance.

Supplementary Note 7: Mutant dosage-based metastatic organotropism.

We then analysed organ-specific patterns of metastasis (Figure 5c,d), using multinomial regression adjusted for sex, age at sequencing, TMB and FGA (Methods; Supplementary Figure S18; full analysis in Supplementary Table S8). We found 33 patterns associated with mutant dosage, including 20 with strong statistical support (FDR-adjusted p-value P ≤ 0.05, Wald Test), and 13 showing borderline evidence (P ≤ 0.1), covering 14 genes and 4 hotspots across 5 primary tumours and 11 metastatic sites.

Beyond global metastatic risk, mutant dosage also predicted specific patterns of metastatic spread. We identified 33 tumour type–specific biomarkers 20 with strong statistical support for which mutant dosage stratified organotropic tendencies of metastasis, revealing a new layer of complexity in the molecular logic of organotropism and extending previous findings based on the independent analysis of mutations and copy number alterations[60]. In hormone-receptor–positive breast cancer, dosage-dependent alterations in epithelial adhesion, hormone

signalling, and differentiation (across CDH1, ESR1, GATA3, TP53) channelled metastatic spread toward ovarian, pulmonary, or lymphatic routes, reflecting how graded perturbation of luminal-lineage circuitry shapes niche preference. In colorectal cancer, different dosage intensities across WNT, RAS/MAPK, and p53 pathways segregated tumours toward intra-abdominal, ovarian, pulmonary, lymphatic, or CNS dissemination, consistent with a modular architecture in which escalating pathway activation or suppression unlocks distinct metastatic competencies[61]. In lung adenocarcinoma, dosage variation across receptor tyrosine kinase and oxidative stress pathways (notably EGFR, KEAP1, ARID1A, PTPRD, PTPRT) delineated local thoracic versus distant organotropism, aligning with known metabolic and microenvironmental constraints of lung-derived metastases[13,62]. In melanoma, dosage-dependent tuning of MAPK, telomerase, and DNA-damage pathways coloured liver, lung, and brain tropism, suggesting that partial versus maximal pathway activation confers distinct microenvironmental compatibilities. In pancreatic cancer, dosage-driven modulation of TP53 and SMAD4 separated intra-abdominal, lymphatic, and pulmonary routes, highlighting how intermediate disruption of chromatin, EMT, or stress-response programs can redirect the metastatic trajectory. These organotropic patterns – decipherable only through integrated mutation and copy-number analysis – open new avenues for understanding and potentially predicting the systemic spread of cancer.

CDH1 in HR+HER2– breast cancer displayed the strongest effect: both balanced (OR = 11.8, CI = 2.80–49.8, P = 0.00273) and high dosages (OR = 9.58, CI = 2.09–43.8, P = 0.00837) conferred a roughly tenfold increase in ovarian metastasis, whereas low dosage showed no significant effect. This sharp contrast may suggest a threshold-like requirement for enhanced epithelial plasticity during peritoneal dissemination. In the same subtype, GATA3 mutations showed a similarly strong but distinct pattern, with both balanced (OR = 3.24, CI = 1.32-7.98, P = 0.0311) and low dosage (OR = 10.4, CI = 3.06-35.7, P = 0.000331) predisposing to lung metastasis, indicating that intermediate activation may optimise migratory or lineage-plastic states required for pulmonary seeding. ESR1 low-dosage mutations were protective against lymphatic dissemination (OR = 0.135, CI = 0.03-0.61, P = 0.0411), whereas TP53 mutations showed a dosage-dependent increase in lymphatic tropism, with balanced (OR = 3.27, CI = 1.48-7.21, P = 0.0102) and low dosages (OR = 7.56, CI = 2.26-25.29, P = 0.00185) associated with progressively higher odds of lymph-node metastasis. These patterns suggest that partial impairment of p53-mediated differentiation programs may favour lymphatic seeding, whereas reduced perturbation of hormone-receptor may confer protection against lymphotropic spread75.

In MSS colorectal cancers, the odds of metastatic spread to central nervous system (CNS) metastases were more than fourfold for high dosage KRAS mutations (OR = 4.64, CI = 1.46-14.8, P = 0.0554), reaching a five-fold for G12 mutations (OR = 5.30, CI = 1.69-16.6, P = 0.00992), suggesting that KRAS-driven metabolic reprogramming and chromosomal instability might be linked to enhanced colonization of the CNS.
Conversely, balanced dosage *KRAS* mutations were associated with higher likelihood of intra-abdominal and especially lung metastases (OR = 1.98, CI = 1.26-3.11, P = 0.0223). APC showed a different, largely suppressive pattern: balanced (OR = 0.353, CI = 0.19-0.65, P = 0.00412) and high-dosage (OR = 0.304, CI = 0.17-0.56, P = 0.000646) mutations significantly reduced intra-abdominal metastasis, while high dosage tended to reduce ovarian dissemination (OR = 0.402, CI = 0.16-1, P = 0.0833). These effects, along with a borderline reduction in CNS metastases (OR = 0.270, CI = 0.08-0.87, P = 0.0694), suggested attenuated WNT-driven migratory potential at higher mutant dosages, with an inverse-dosage organotropism76. PIK3CA mutations displayed an opposite pattern: low dosage strongly increased ovarian metastasis (OR = 7.41, CI = 1.28-42.78, P = 0.0351), highlighting a low dosage optimum for PI3K-driven peritoneal spread77.

In lung adenocarcinoma, multiple genes showed dosage-dependent behaviour. Balanced-dosage KEAP1 mutations increased bone metastasis (OR = 2.22, CI = 1.3-3.8, P = 0.0117), whereas high-dosage mutations increased head-and-neck metastases, albeit with borderline significance (OR = 6.26, CI = 1.12-35.0, P = 0.0737). This divergence highlighted a dosage-stratified organotropic spectrum, in which increasing levels of KEAP1

inactivation may differentially support metastatic fitness across anatomical sites78. EGFR balanced-dosage mutations also increased bone metastasis (OR = 1.91, CI = 1.13-3.25, P = 0.0543), while high-dosage mutations increased liver involvement (OR = 2.03, CI = 1.1-3.74, P = 0.0574) and low dosage strongly increased mediastinal metastasis (OR = 3.94, CI = 1.29-12.09, P = 0.0327). The E746 A750del hotspot amplified these patterns, with high dosage predicting liver (OR = 5.04, CI = 1.62-15.65, P = 0.0103) and balanced dosage predicting pleural metastasis (OR = 2.52, CI = 1.09-5.86, P = 0.0774). The L858R hotspot at balanced dosage further increased lung metastases (OR = 7.52, CI = 1.82-31.1, P = 0.0134). These results revealed highly allele- and dosage-dependent organotropic routes for EGFR-driven tumours. KRAS showed a complementary pattern: low-dosage mutations, whether general (OR = 0.328, CI = 0.15-0.70, P = 0.0104) or G12 (OR = 0.405, CI = 0.19-0.88, P = 0.0540), were associated with reduced odds of pleural metastasis, suggesting that intermediate levels of RAS-GTP signalling may be insufficient to support pleural colonisation. PTPRT and PTPRD mutations showed weaker but consistent effects: balanced-dosage PTPRT mutations were associated with reduced liver involvement (OR = 0.279, CI = 0.09-0.84, P = 0.0596), whereas high-dosage PTPRD mutations showed a borderline-significant increase in adrenal spread (OR = 3.90, CI = 1.15-13.24, P = 0.0576). Although modest in statistical strength, these patterns suggested that phosphatase-mediated modulation of signalling thresholds may contribute to organ-specific metastatic compatibility.

In pancreatic adenocarcinoma, both balanced (OR = 0.252, CI = 0.12-0.54, P = 0.00114) and low (OR = 0.439, CI = 0.21-0.91, P = 0.0802) TP53 mutant dosage were associated with reduced intra-abdominal dissemination, and balanced dosage also corresponded to a lower likelihood of lymphnode involvement (OR = 0.216, CI = 0.08-0.56, P = 0.00262). Balanced-dosage SMAD4 mutations showed strong lung tropism (OR = 4.30, CI = 1.48-12.53, P = 0.0375), suggesting that partial suppression of TGF-β signals might enhance epithelial–mesenchymal plasticity required for pulmonary seeding80.

In melanoma, high (OR = 0.349, CI = 0.15-0.81, P = 0.0882) and balanced (OR = 0.212, CI = 0.08-0.55, P = 0.00917) BRAF mutations, including the V600E hotspot, were protective from liver metastases. TP53 mutations showed an enrichment for CNS metastasis only at balanced dosage (OR = 0.208, CI = 0.07-0.65, P = 0.0399), indicating that modest p53 impairment may facilitate neural-niche colonisation.

Overall, these findings demonstrate that metastatic tropism is not simply mutation-specific but is frequently dosage-dependent, with distinct dosage classes within a single gene often predisposing tumours to entirely different organotropic routes.

Supplementary Tables

- Supplementary Table S1: Priors on (k,m) from WGS data
- Supplementary Table S2: Priors on count rate per copy
- Supplementary Table S3: Computational efficiency
- Supplementary Table S4: Survival analysis (KM)
- Supplementary Table S5: Survival analysis (Cox)
- Supplementary Table S6: Mutant Dosage vs Adjusted VAF
- Supplementary Table S7: Survival analysis at (k,m) resolution

- Supplementary Table S8: Mutant Dosage enrichment
- Supplementary Table S9: Metastatic propensity
- Supplementary Table S10: Metastatic tropism
- Supplementary Table S11: HMF to INTOGEN conversion

Supplementary Figures

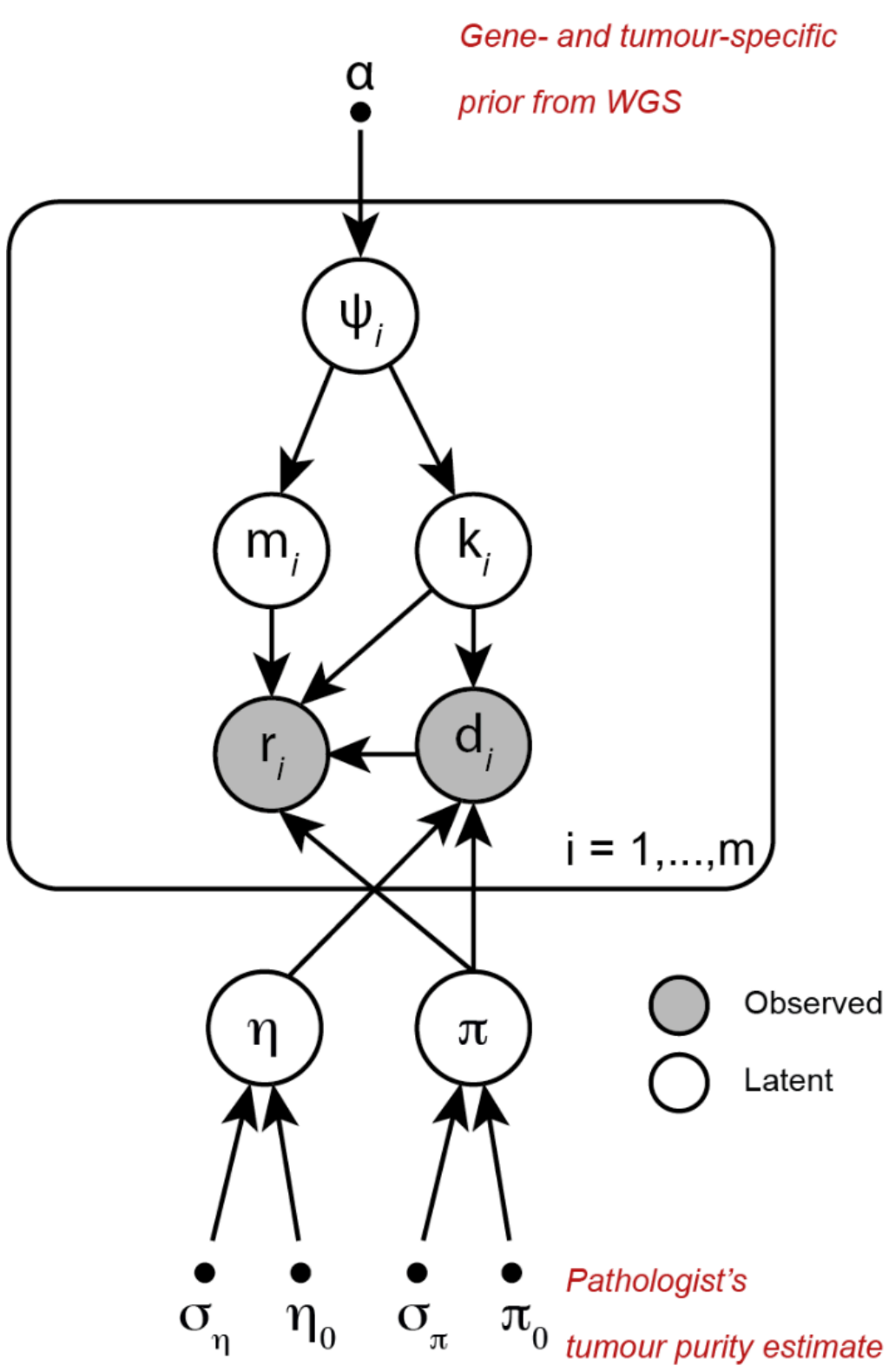

$$\psi_i \sim \text{Dirichlet}(\alpha_i)$$

$$d_i \mid \pi, \eta, k_i \sim \text{Poisson}(\lambda_i) \qquad \lambda_i = (1-\pi)2\eta + \pi k_i \eta$$

$$r_i \mid \pi, k_i, m_i \sim \text{Binomial}(\varphi_i) \qquad \varphi_i = \frac{m_i \pi}{2(1-\pi) + k_i \pi}$$

$$\pi \mid \pi_0, \sigma_\pi \sim \text{Beta}(\alpha_\pi, \beta_\pi) \qquad \alpha_\pi = \pi_0\left[\frac{\pi_0(1-\pi_0)}{\sigma_\pi^2} - 1\right] \qquad \beta_\pi = (1-\pi_0)\left[\frac{\pi_0(1-\pi_0)}{\sigma_\pi^2} - 1\right]$$

$$\eta_i = \sum_k \frac{p(k) d_i}{2(1-\pi_0) + k\pi_0}$$

$$\eta_0 = \frac{1}{m} \sum_{i=1}^{m} \eta_i$$

$$\eta \mid \eta_0, \sigma_\eta \sim \text{Gamma}(\alpha_\eta, \beta_\eta) \qquad \sigma_\eta^2 = \frac{1}{m-1} \sum_{i=1}^{m} (\eta_i - \eta_0)^2$$

$$\alpha_\eta = \frac{\eta_0^2}{\sigma_\eta^2} \qquad \beta_\eta = \frac{\eta_0}{\sigma_\eta^2}$$

Supplementary Figure S1: Probabilistic graphical model of INCOMMON. Unobserved (latent) variables are indicated as white circles, observed variables as grey circles and fixed hyperparameters as dots. Prior probabilities ψ for each combination of mutation total copy number k and multiplicity m are sampled from a Dirichlet distribution, with concentration parameters α reflecting the relative fraction observed from large-scale WGS cohorts. The prior probability for tumour purity π is centred at the pathologist's estimate $\pi 0$ with fixed variance $\sigma\pi$, whereas the mean $\eta 0$ and variance $\sigma\eta$ of the prior probability for the read count rate per chromosome copy η are estimated from the data. The total number of reads at each site d follows a Poisson distribution with rate λ contributed by normal ($(1 - \pi)2\eta$) and tumour ($\pi k\eta$) cells. The number of reads with the variant r follows a Binomial distribution with success probability ϕ defined as the expected variant allele frequency $m\pi/(2(1 - \pi) + k\pi)$.

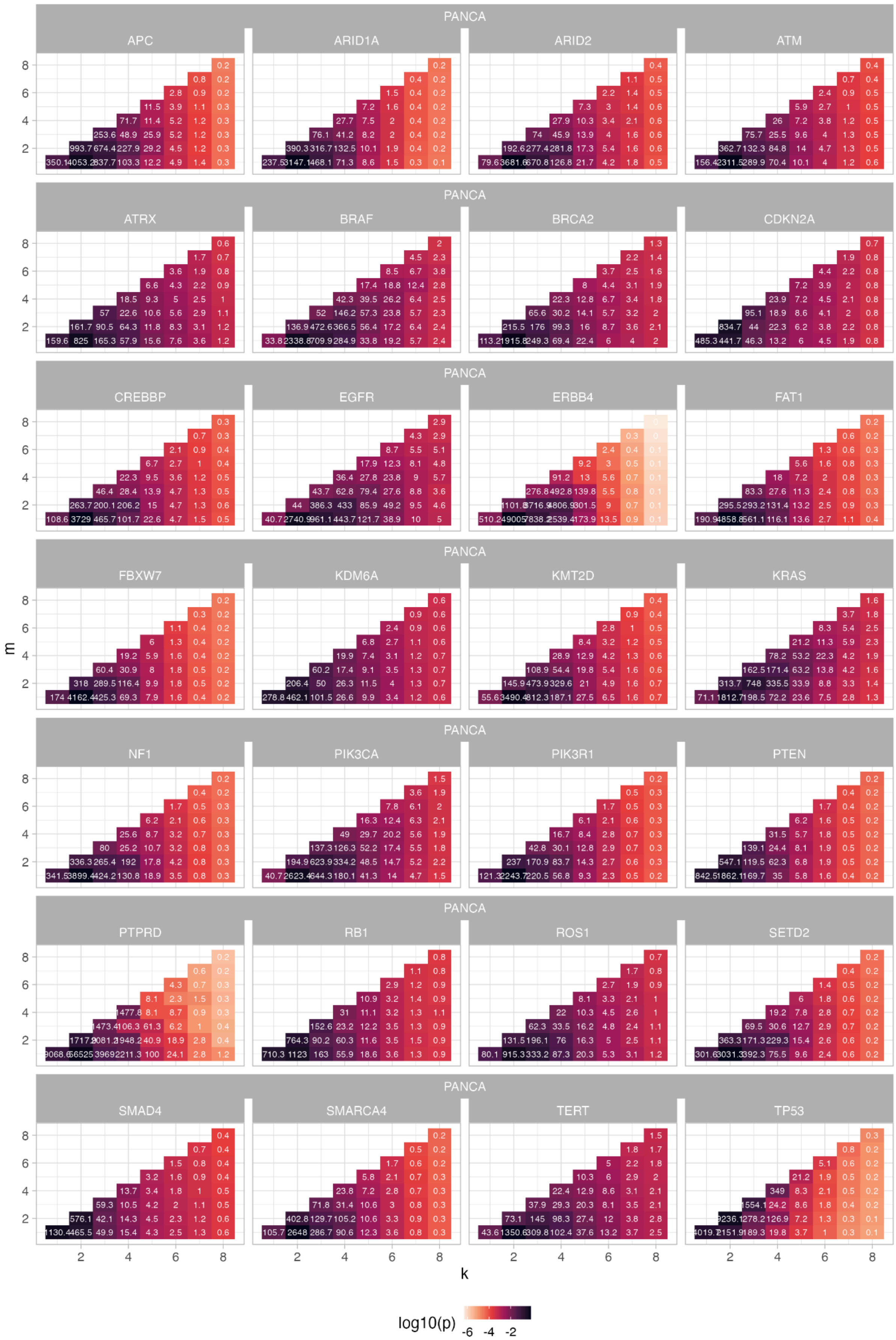
PANCA
APC
ARID1A
ARID2
ATM
ATRX
BRAF
BRCA2
CDKN2A
CREBBP
EGFR
ERBB4
FAT1
FBXW7
KDM6A
KMT2D
KRAS
NF1
PIK3CA
PIK3R1
PTEN
PTPRD
RB1
ROS1
SETD2
SMAD4
SMARCA4
TERT
TP53
m
k
log10(p)

Supplementary Figure S2: Pan-cancer (PANCA) prior distribution on the copy number and multiplicity configurations (k, m) obtained from the PCAWG and HMF whole genome sequencing cohorts. The colour of each cell corresponds to the relative fraction of the configuration in the dataset, while the number label inside is equal to the actual count (or pseudo-count, see Methods), used as a Dirichlet concentration parameter.

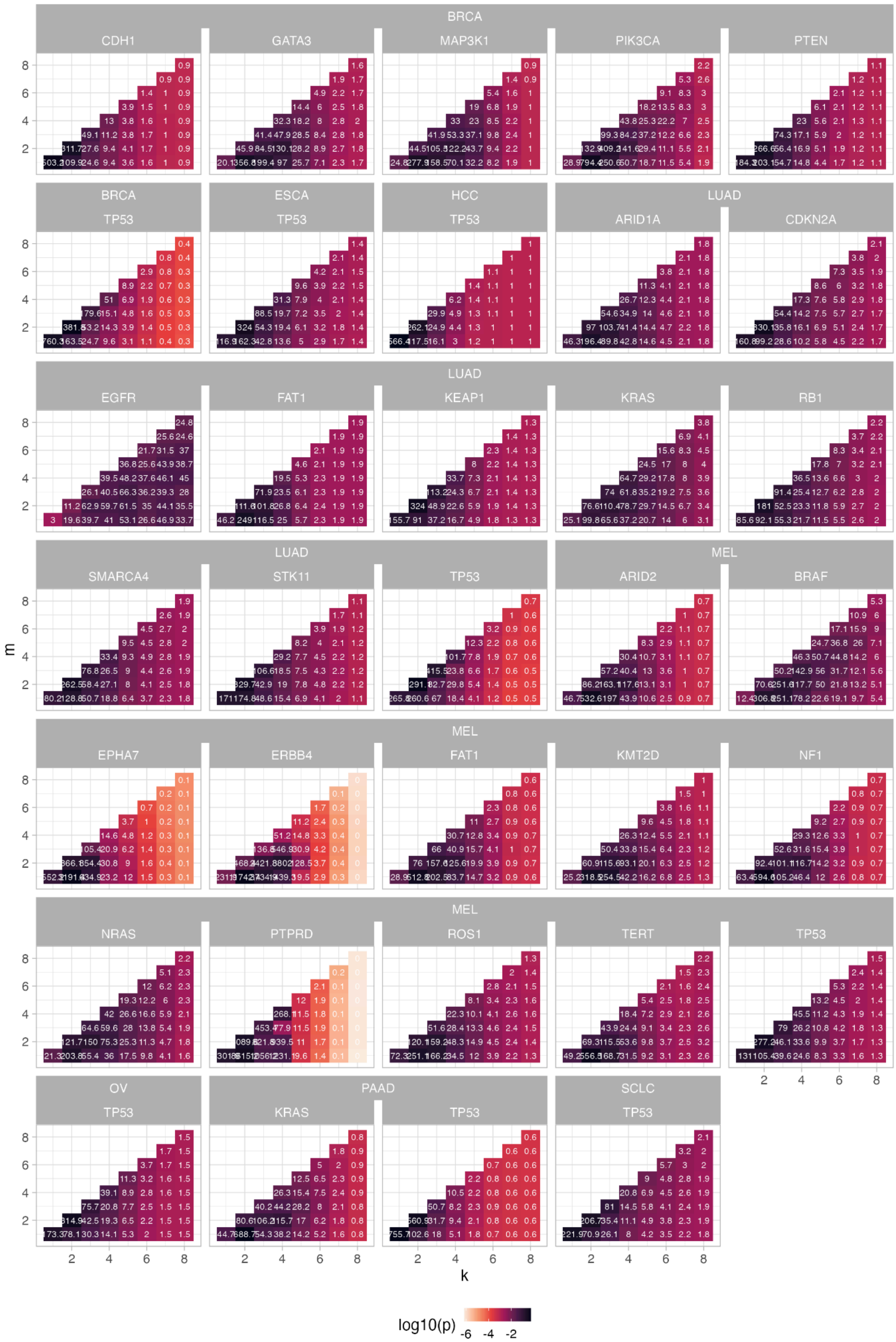
BRCA
CDH1
GATA3
MAP3K1
PIK3CA
PTEN
BRCA
TP53
ESCA
TP53
HCC
TP53
LUAD
ARID1A
CDKN2A
LUAD
EGFR
FAT1
KEAP1
KRAS
RB1
LUAD
SMARCA4
STK11
TP53
MEL
ARID2
BRAF
MEL
EPHA7
ERBB4
FAT1
KMT2D
NF1
MEL
NRAS
PTPRD
ROS1
TERT
TP53
OV
TP53
PAAD
KRAS
TP53
SCLC
TP53
m
k
log10(p)
-6
-4
-2

Supplementary Figure S3: Tumour type-specific prior distribution on the copy number and multiplicity configurations (k, m) obtained from the PCAWG and HMF whole genome sequencing cohorts. The colour of each cell corresponds to the relative fraction of the configuration in the dataset, while the number label inside is equal to the actual count (or pseudo-count, see Methods), used as a Dirichlet concentration parameter.

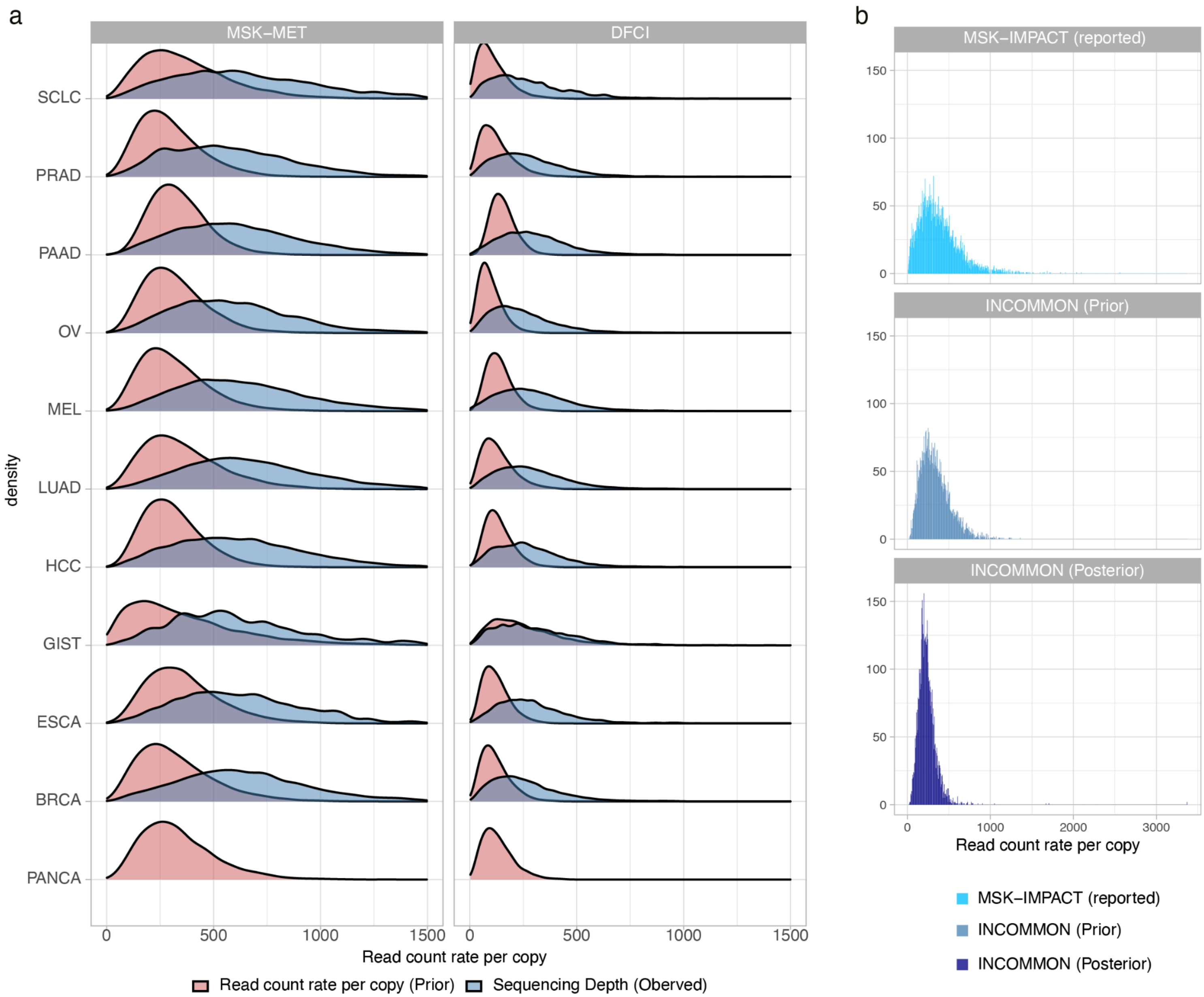

Supplementary Figure S4: a. Gamma prior distribution (red) over the read count per chromosome copy, compared with the observed distribution of sequencing depth (blue), across the MSK-MET and GENIE-DFCI cohorts. Notice that the empirical distribution of sequencing depth is confounded by mutation copy number and tumour purity. b. Read count rate per chromosome copy as reported for the MSK-IMPACT targeted panel[21], aligning with prior and posterior INCOMMON distributions.

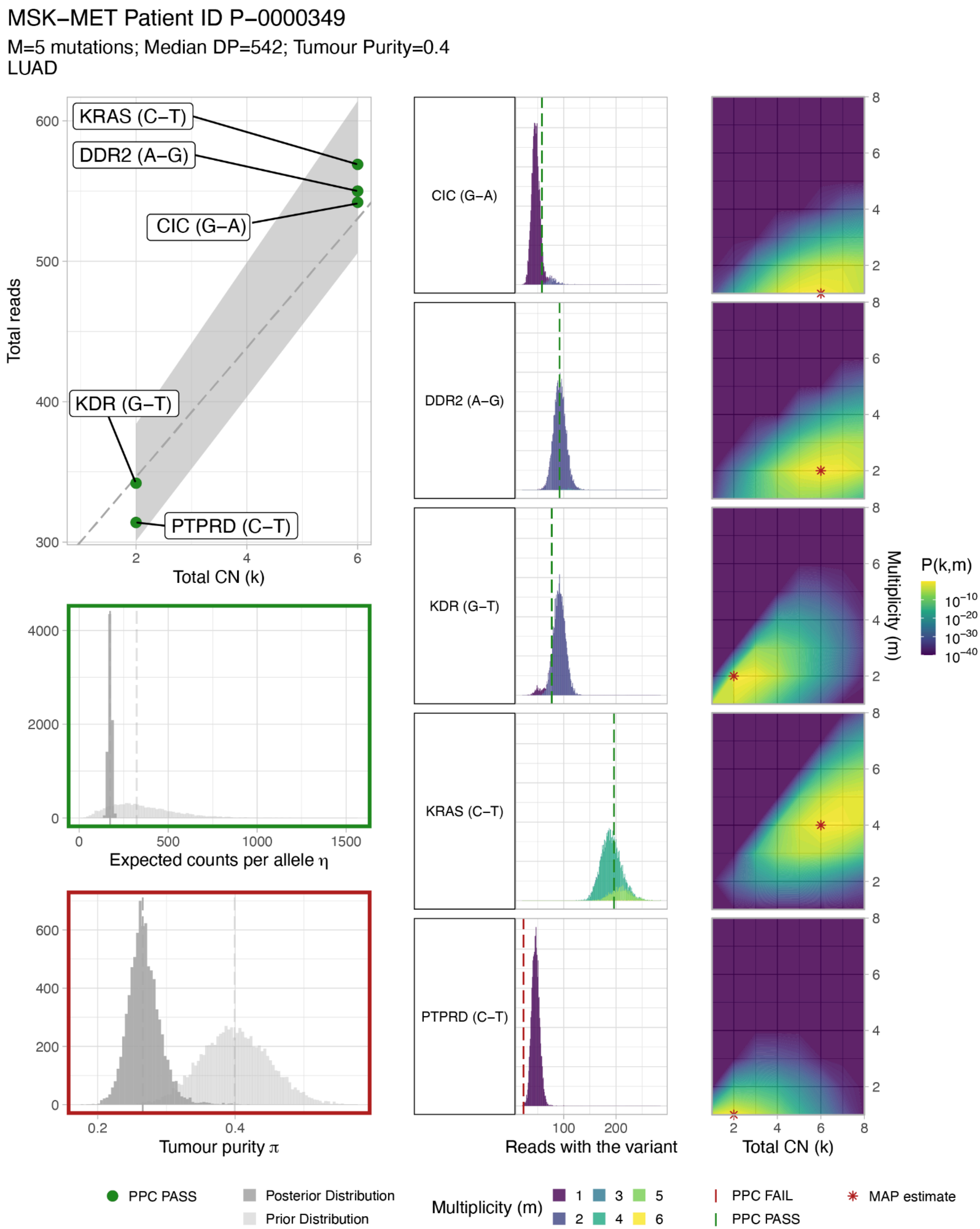
MSK−MET Patient ID P−0000349
M=5 mutations; Median DP=542; Tumour Purity=0.4
LUAD
KRAS (C−T)
DDR2 (A−G)
CIC (G−A)
KDR (G−T)
PTPRD (C−T)
Total reads
Total CN (k)
Expected counts per allele η
Tumour purity π
Reads with the variant
Multiplicity (m)
P(k,m)
PPC PASS
Posterior Distribution
Prior Distribution
PPC FAIL
MAP estimate

Supplementary Figure S5: INCOMMON report for MSK-MET lung cancer patient P-0000349. All posterior predictive checks (PPC) are passed, except for tumour purity (99.4% of samples from the posterior distribution - mean π = 0.251 - deviate from the prior mean π_0 = 0.4 more than expected a priori) and multiplicity of the C-T mutation on PTPRD gene (the observed number of reads with the variant NV = 23 lies in the lower 5% tail of the posterior predictive distribution, indicating a poor fit).

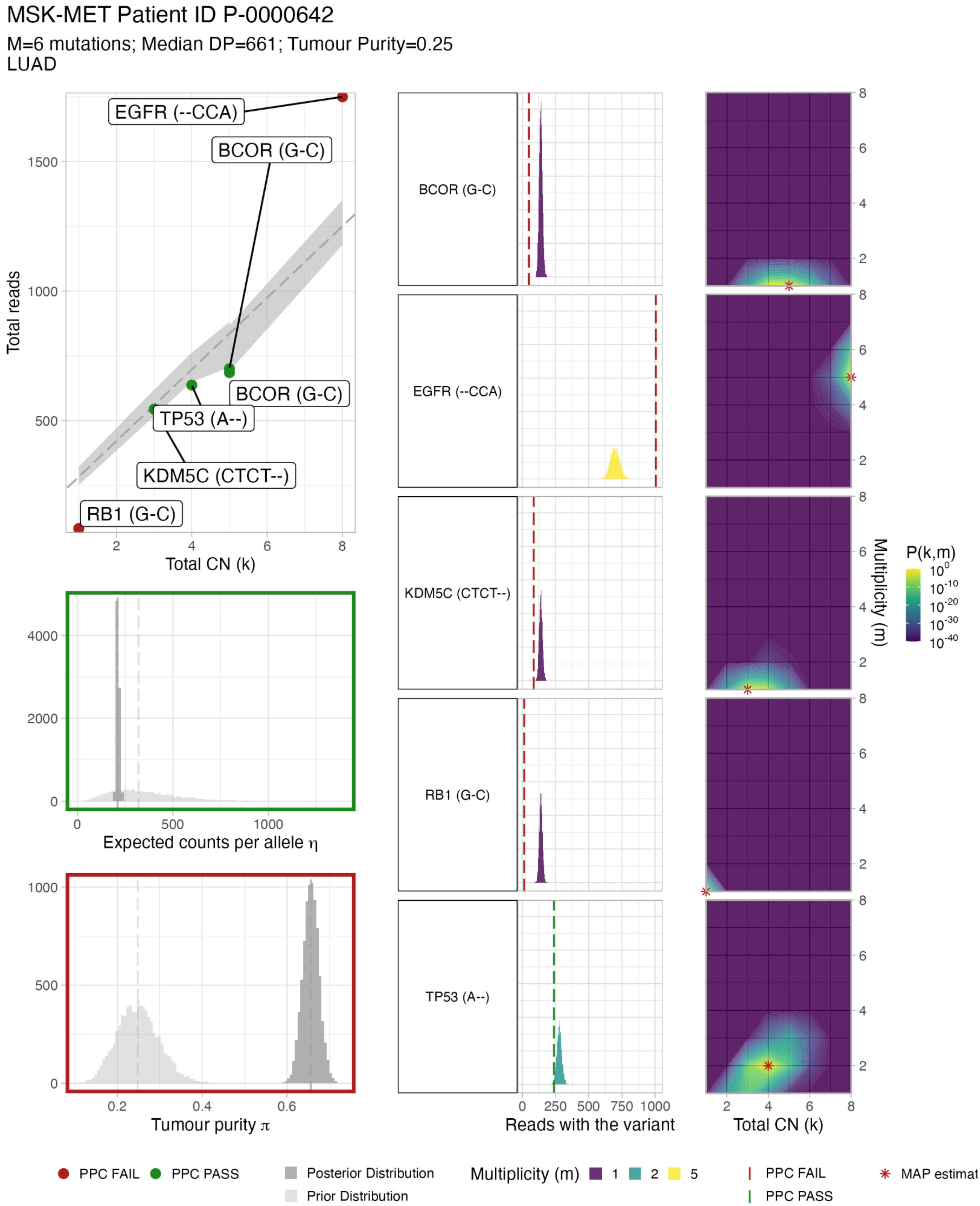

Supplementary Figure S6: INCOMMON report for MSK-MET lung cancer patient P-0000642, a case of poor fit, failing posterior-predictive checks (PPC) on total copy number for G-C substitution on RB1 and CCA insertion on EGFR, tumour

purity (inferred π = 0.641 versus prior estimate π_0 = 0.25, with no overlap between prior and posterior distributions), multiplicity of on all sequenced alterations except for an A deletion on TP53.

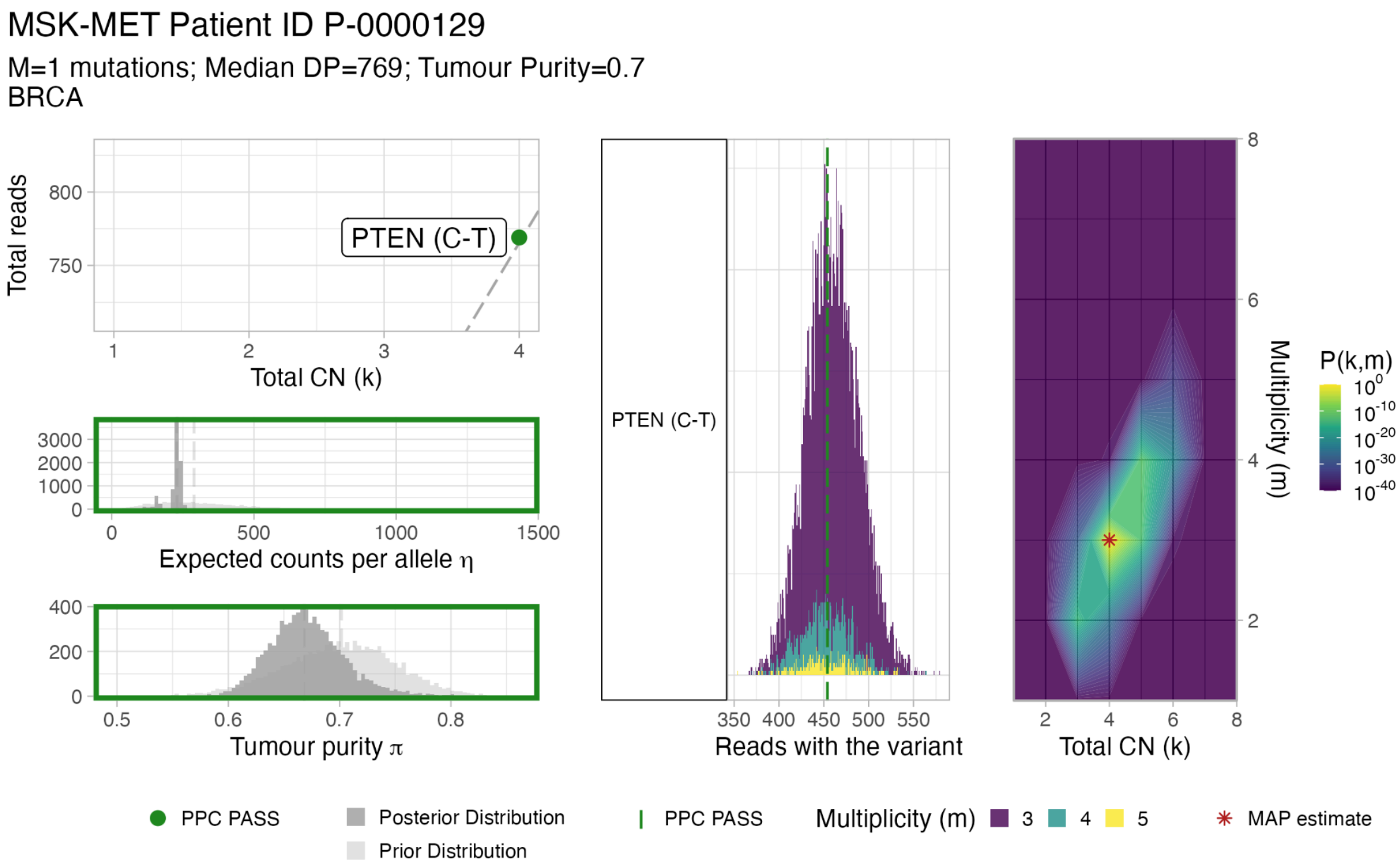

Supplementary Figure S7: INCOMMON report for MSK-MET breast cancer patient P-0000129, a particular case with one single mutation, a C-T substitution in the PTEN gene, is detected by the targeted panel assay. Even if one single data point is available, INCOMMON is able to infer the expected read counts per allele η, the tumour purity π, and the mutation total copy number and multiplicity (k = 4, m = 3), in this case. The inference is feasible thanks to the Bayesian formulation of INCOMMON, which allows leveraging prior distributions for the model parameters $\Theta = (\pi, \eta, k, m)$.

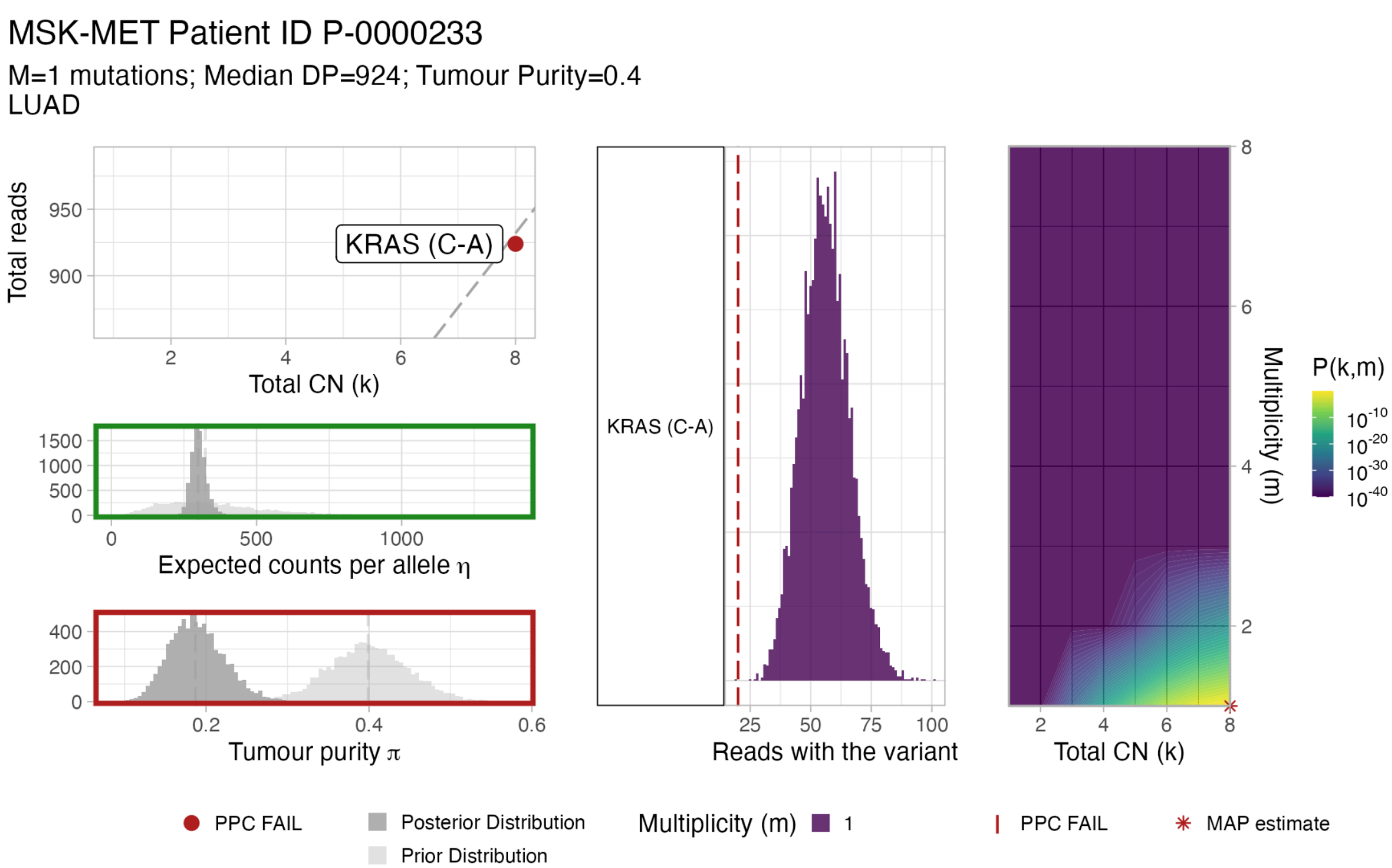

Supplementary Figure S8: INCOMMON report for MSK-MET lung cancer patient P-0000233, a case with one single mutation, a C-A substitution in the KRAS gene, is detected by the targeted panel assay. In this particular case, posterior-predictive checks indicate a poor fit of total copy-number (total number of reads lower than expected), tumour purity (deviation from prior input purity outside the allowed error of $\epsilon = \pm 10 - 15\%$), and multiplicity (number of reads with the variant lower than expected). This demonstrates the capability of INCOMMON to flag samples with a poor fit.

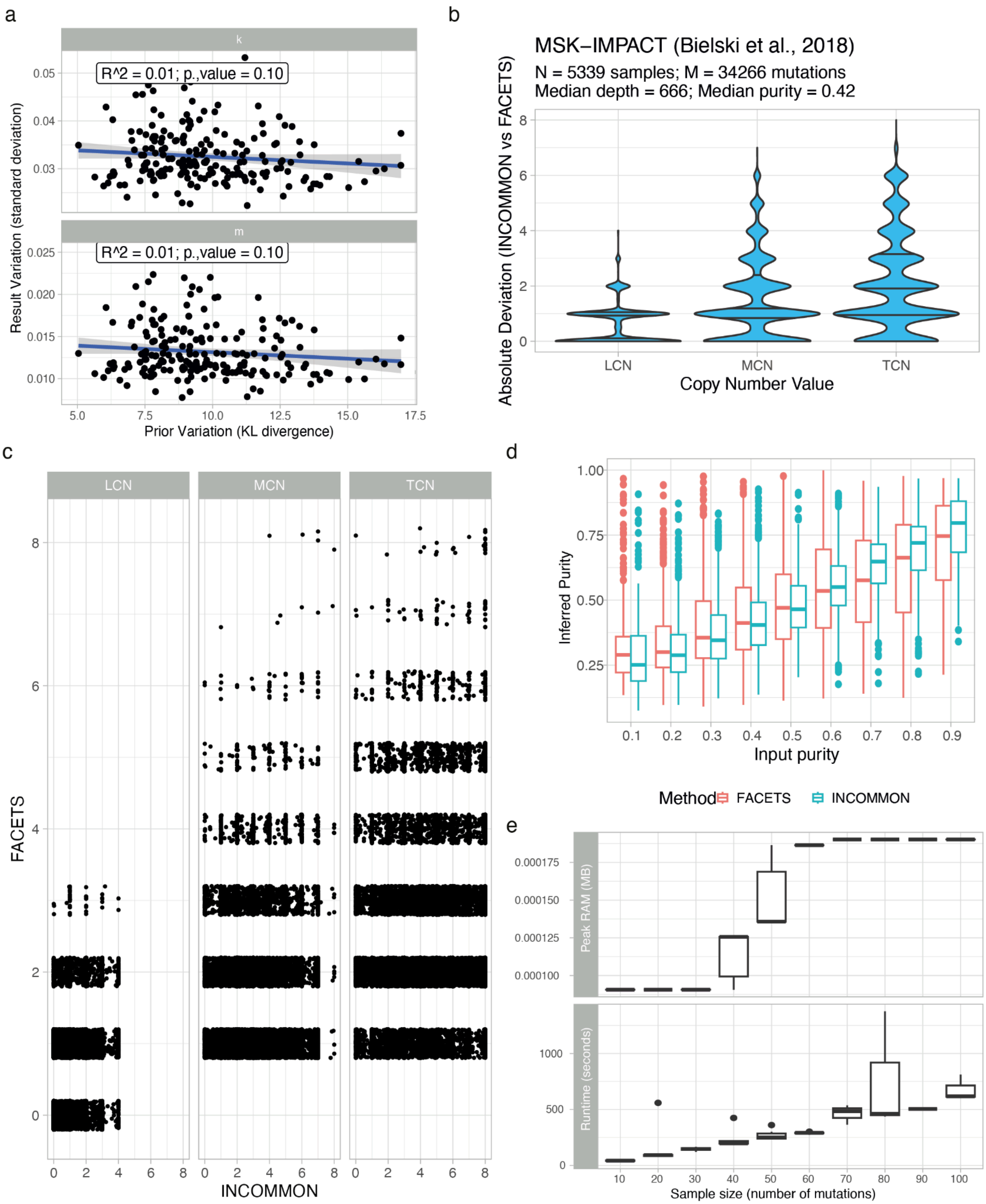

Supplementary Figure S9: a. Standard deviation of the mean absolute error (MAE) estimates against prior variation measured by Kullback-Leibler divergence, across N = 50 iterations of random-splitting (70% training, 30% test) of the combined PCAWG and HMF data. b,c. Comparison of INCOMMON and FACETS copy-number calls across the MSK-IMPACT dataset published in Bielski et. al. d. Comparison of INCOMMON and FACETS inference of tumour purity. e.

The low peak RAM usage (top panel) and runtime required for the classification of samples of increasing size (number of mutations) demonstrate the high computational efficiency of INCOMMON.

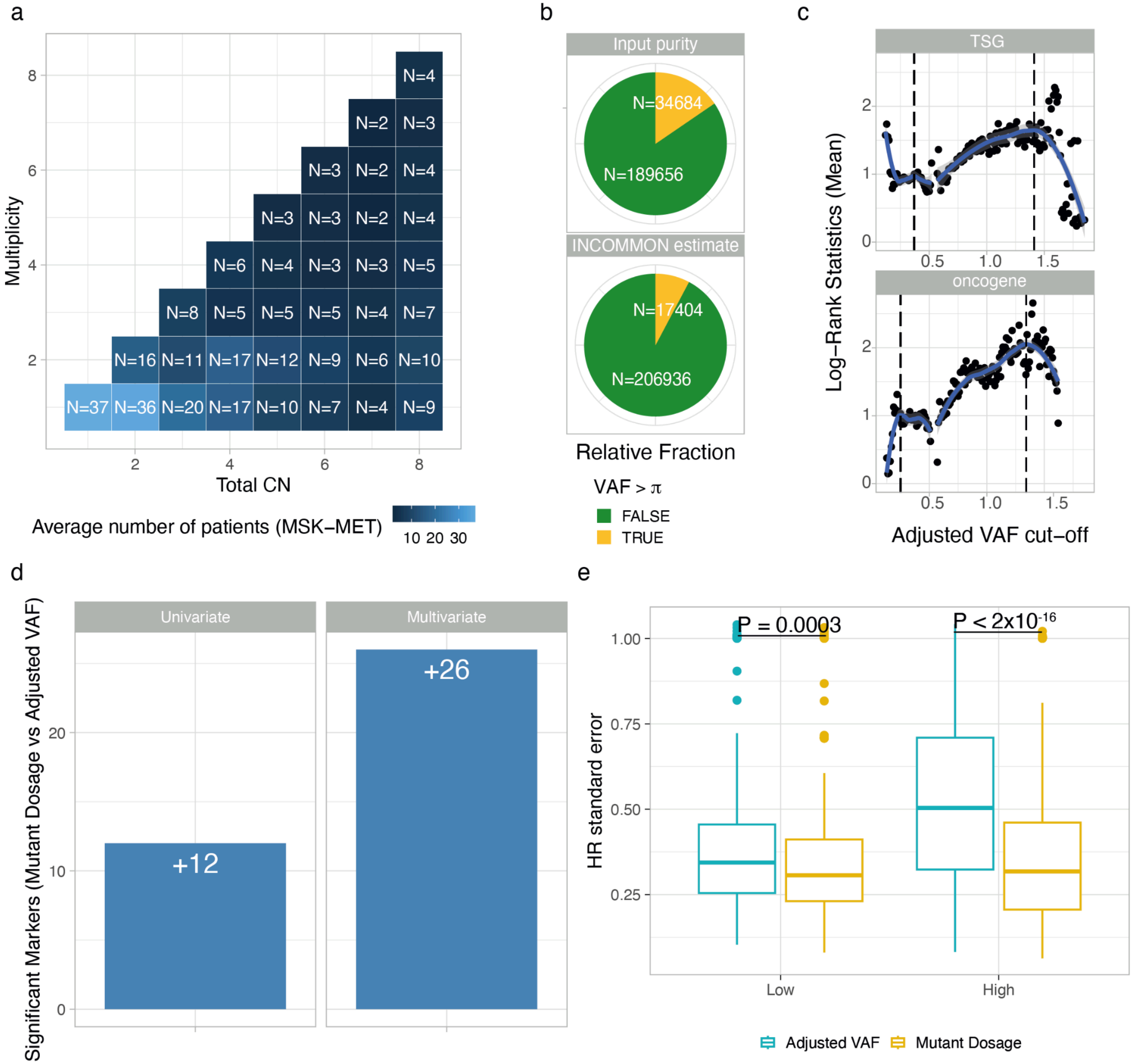

Supplementary Figure S10: a. Average number of patients per (k, m) configuration in the MSK-MET cohort, across genes and tumour types. b. The relative fraction of MSK-MET samples with inconsistent VAF and tumour purity (VAF > π) decreases when using the INCOMMON estimate instead of the publicly available input purity. c. Optimal adjusted VAF (VAF/π) thresholds for patient stratification into Low, Intermediate and High adjusted VAF, maximising the average Log-Rank statistics across TSGs, oncogenes and tumour types in the MSK-MET cohort. d. Absolute increase in the number of significant Mutant Dosage markers with respect to adjusted VAF classification, in univariate and multivariate Cox regression (using patients'

age, sex, TMB, FGA and sample type as covariates). e. The standard error of hazard ratios (HR) in multivariate Cox regression, using adjusted VAF stratification, is significantly higher than in Mutant Dosage stratification.

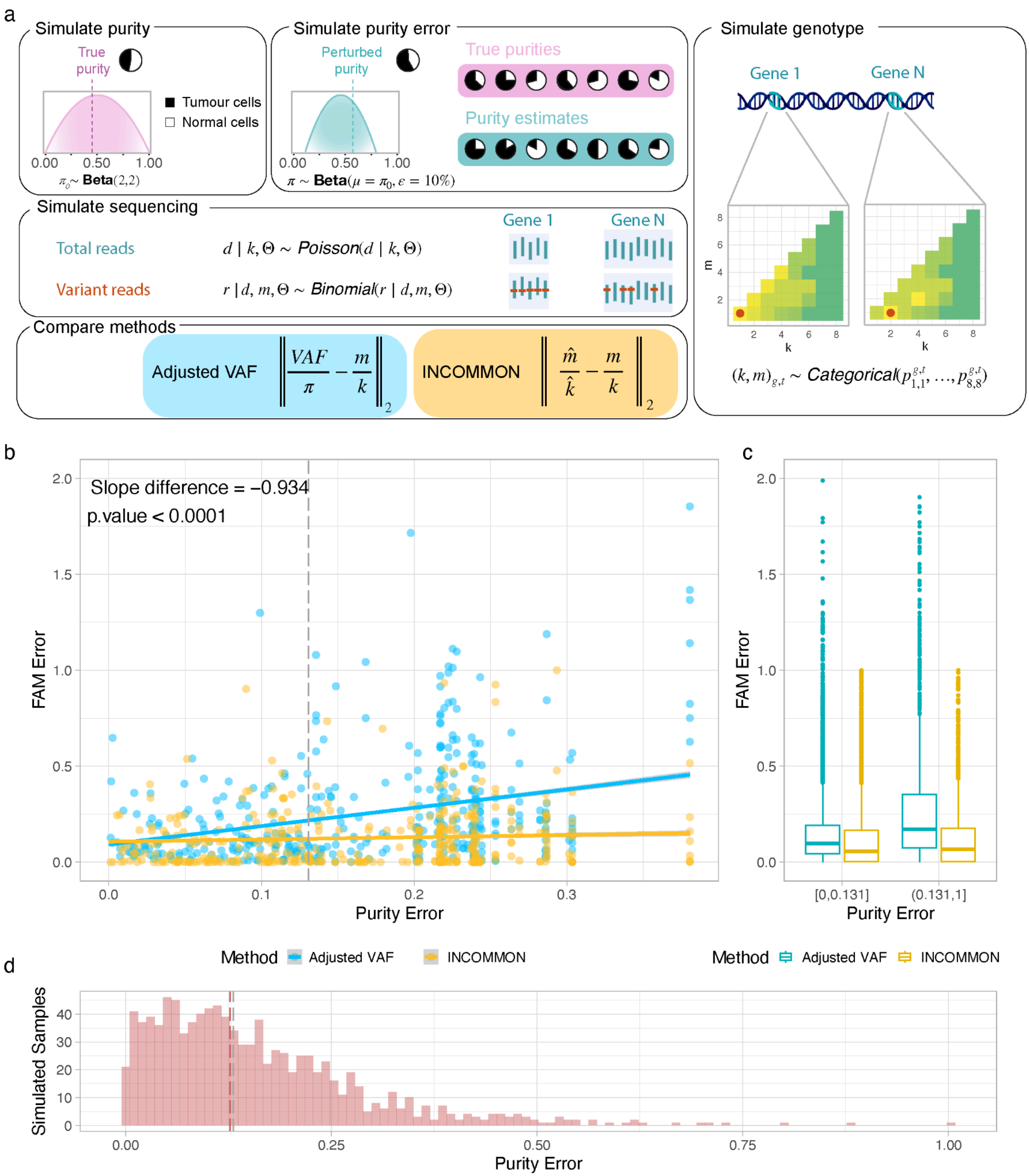

Supplementary Figure S11: a. Simulation framework used to compare INCOMMON and adjusted VAF approaches: tumour purities were sampled from a Beta distribution, then perturbed to introduce random estimation error. Mutation copy number (k) and multiplicity (m) for randomly sampled mutant genes were sampled from empirical prior distributions, followed by

simulation of sequencing read counts. Estimated FAM values from both methods were compared against the ground truth. b,c. Adjusted VAF exhibits substantially larger error than INCOMMON as the purity error increases. d. Distribution of simulated purity errors, with median value (red line) matching that of TCGA consensus purity estimates (grey line).

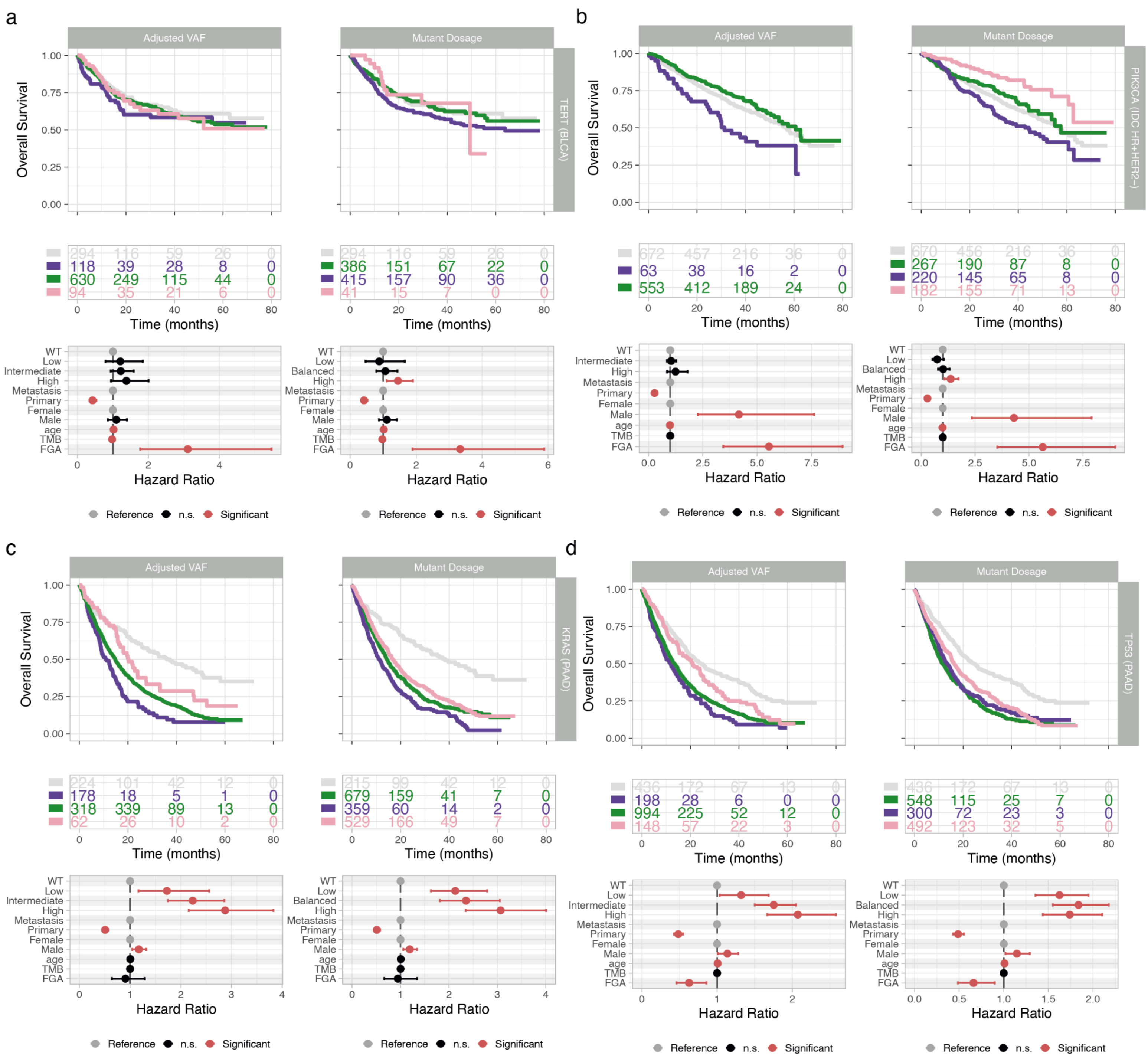

Supplementary Figure S12: Representative examples comparing Mutant Dosage and Adjusted VAF stratifications for overall survival (OS). Both approaches define three classes (Low, Intermediate/Balanced, High) and are evaluated using Kaplan–Meier curves and multivariate Cox regression adjusted for primary vs. metastatic status, sex, age, TMB, and FGA. a–b. Differences in prognostic marker discovery rates arise because Adjusted VAF generally mirrors the trend of decreasing

OS with increasing Mutant Dosage, but often fails to reach statistical significance due to higher uncertainty in hazard ratio (HR) estimates. Examples include TERT-mutant bladder cancer and PIK3CA-mutant HR+/HER2- breast cancer. c. In most settings, both methods identify similar prognostic groups with comparable HR estimates, although Adjusted VAF typically produces wider confidence intervals; KRAS-mutant pancreatic cancer illustrates this case. d. In a smaller set of cases, adjusted VAF yields the same number of classes but assigns more distinct and interpretable HR values across groups than Mutant Dosage; TP53-mutant pancreatic adenocarcinoma is an example.

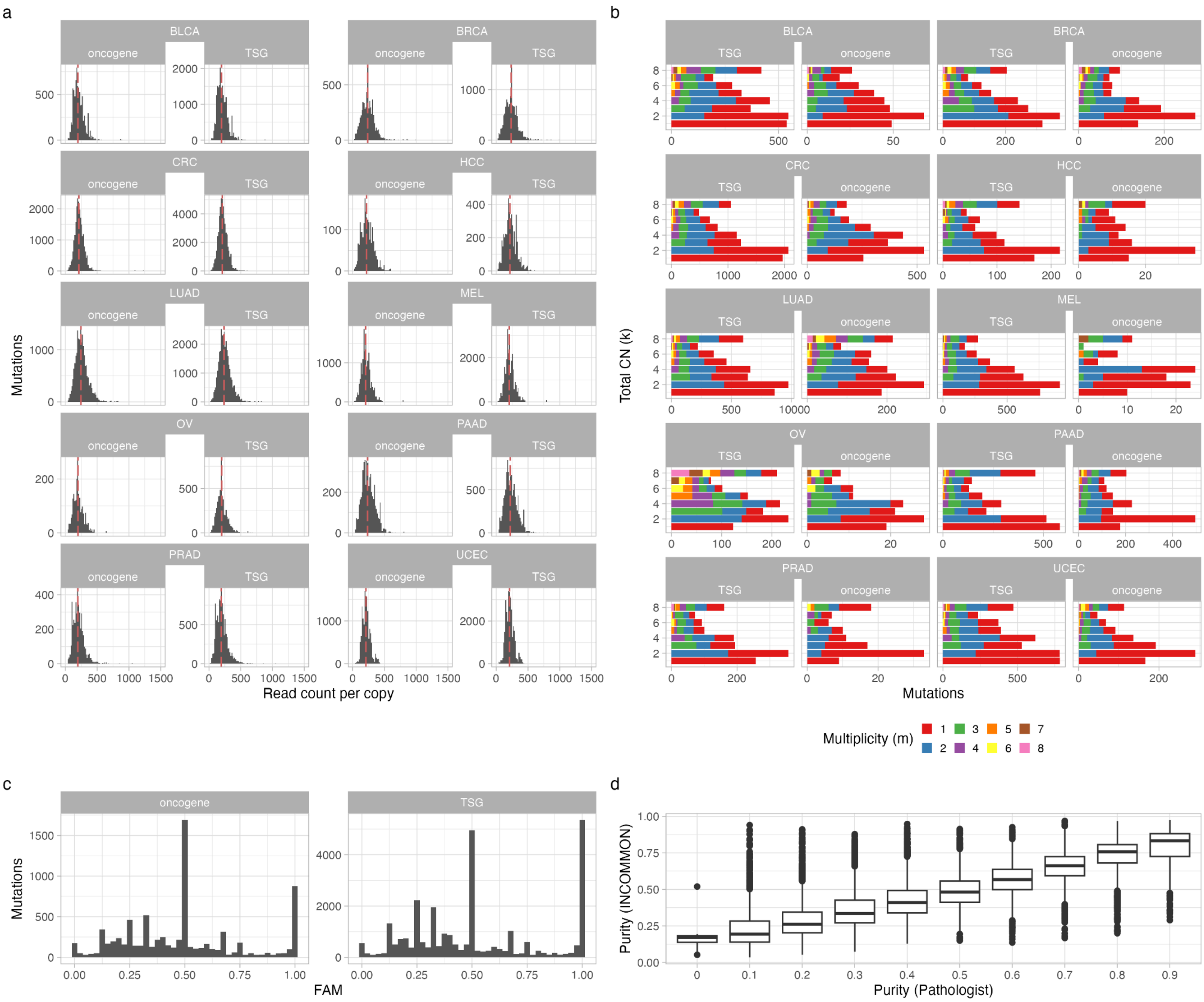

Supplementary Figure S13: a. Distribution of maximum a posteriori (MAP) values of read count rate per copy η, across the main tumour types of the MSK-MET cohort. Red lines indicate median values. b. Distribution of MAP values of total copy number k and multiplicity m. c. Distribution of the MAP values of FAM. d. Comparison of MAP values of the sample purities with the pathologists' estimates.

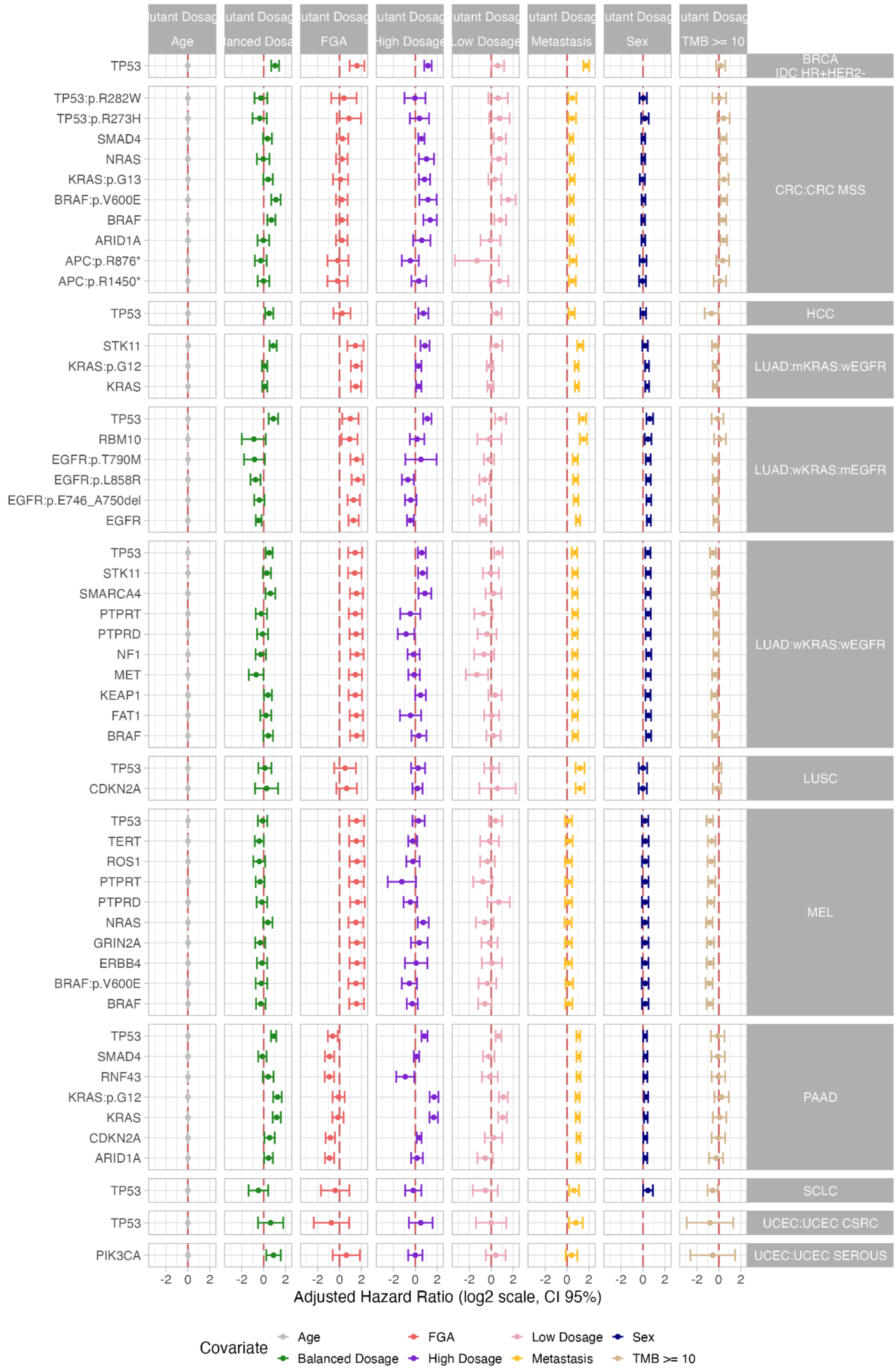
utant Dosag
Age
anced Dosa
FGA
High Dosage
ow Dosage
Metastasis
Sex
TMB >= 10
BRCA
IDC HR+HER2-
CRC:CRC MSS
HCC
LUAD:mKRAS:wEGFR
LUAD:wKRAS:mEGFR
LUAD:wKRAS:wEGFR
LUSC
MEL
PAAD
SCLC
UCEC:UCEC CSRC
UCEC:UCEC SEROUS
TP53
TP53:p.R282W
TP53:p.R273H
SMAD4
NRAS
KRAS:p.G13
BRAF:p.V600E
BRAF
ARID1A
APC:p.R876*
APC:p.R1450*
STK11
KRAS:p.G12
KRAS
RBM10
EGFR:p.T790M
EGFR:p.L858R
EGFR:p.E746_A750del
EGFR
SMARCA4
PTPRT
PTPRD
NF1
MET
KEAP1
FAT1
CDKN2A
TERT
ROS1
GRIN2A
ERBB4
RNF43
PIK3CA
-2 0 2
Adjusted Hazard Ratio (log2 scale, CI 95%)
Covariate
Age
Balanced Dosage
FGA
High Dosage
Low Dosage
Metastasis
Sex
TMB >= 10

Supplementary Figure S14: Adjusted hazard ratios (HR, log2 scale) with 95% confidence interval for all the covariates included in the multivariate Cox regression models of overall survival. The included covariates are mutant dosage class, age, sex, TMB, FGA and sample type. Red vertical lines indicate HR = 1. For each tumour type, only the most frequently mutated genes are shown.

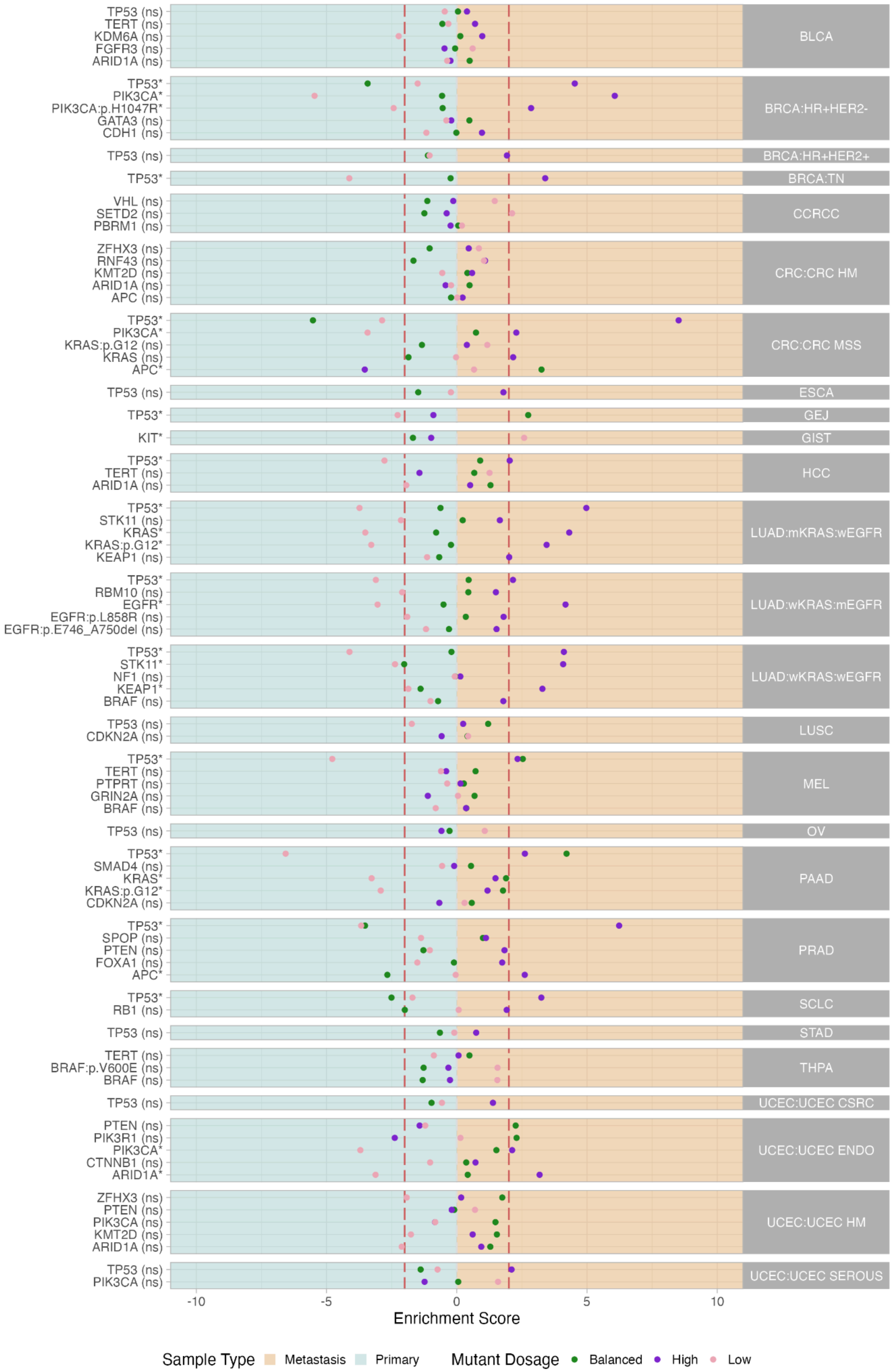

TP53 (ns)
TERT (ns)
KDM6A (ns)
FGFR3 (ns)
ARID1A (ns)
BLCA
TP53*
PIK3CA*
PIK3CA:p.H1047R*
GATA3 (ns)
CDH1 (ns)
BRCA:HR+HER2-
TP53 (ns)
BRCA:HR+HER2+
TP53*
BRCA:TN
VHL (ns)
SETD2 (ns)
PBRM1 (ns)
CCRCC
ZFHX3 (ns)
RNF43 (ns)
KMT2D (ns)
ARID1A (ns)
APC (ns)
CRC:CRC HM
TP53*
PIK3CA*
KRAS:p.G12 (ns)
KRAS (ns)
APC*
CRC:CRC MSS
TP53 (ns)
ESCA
TP53*
GEJ
KIT*
GIST
TP53*
TERT (ns)
ARID1A (ns)
HCC
TP53*
STK11 (ns)
KRAS*
KRAS:p.G12*
KEAP1 (ns)
LUAD:mKRAS:wEGFR
TP53*
RBM10 (ns)
EGFR*
EGFR:p.L858R (ns)
EGFR:p.E746_A750del (ns)
LUAD:wKRAS:mEGFR
TP53*
STK11*
NF1 (ns)
KEAP1*
BRAF (ns)
LUAD:wKRAS:wEGFR
TP53 (ns)
CDKN2A (ns)
LUSC
TP53*
TERT (ns)
PTPRT (ns)
GRIN2A (ns)
BRAF (ns)
MEL
TP53 (ns)
OV
TP53*
SMAD4 (ns)
KRAS*
KRAS:p.G12*
CDKN2A (ns)
PAAD
TP53*
SPOP (ns)
PTEN (ns)
FOXA1 (ns)
APC*
PRAD
TP53*
RB1 (ns)
SCLC
TP53 (ns)
STAD
TERT (ns)
BRAF:p.V600E (ns)
BRAF (ns)
THPA
TP53 (ns)
UCEC:UCEC CSRC
PTEN (ns)
PIK3R1 (ns)
PIK3CA*
CTNNB1 (ns)
ARID1A*
UCEC:UCEC ENDO
ZFHX3 (ns)
PTEN (ns)
PIK3CA (ns)
KMT2D (ns)
ARID1A (ns)
UCEC:UCEC HM
TP53 (ns)
PIK3CA (ns)
UCEC:UCEC SEROUS
-10
-5
0
5
10
Enrichment Score
Sample Type
Metastasis
Primary
Mutant Dosage
Balanced
High
Low

Supplementary Figure S15: Enrichment scores r in primary tumours vs metastases of low, balanced and high mutant dosage classes, across the most frequently mutated genes per tumour type in the MSK-MET cohort. The enrichment score is evaluated through a two-tailed Fisher's exact test with a significance level α = 0.1 and using standardised residuals. Vertical dashed lines indicate thresholds of large effect size (r = 2).

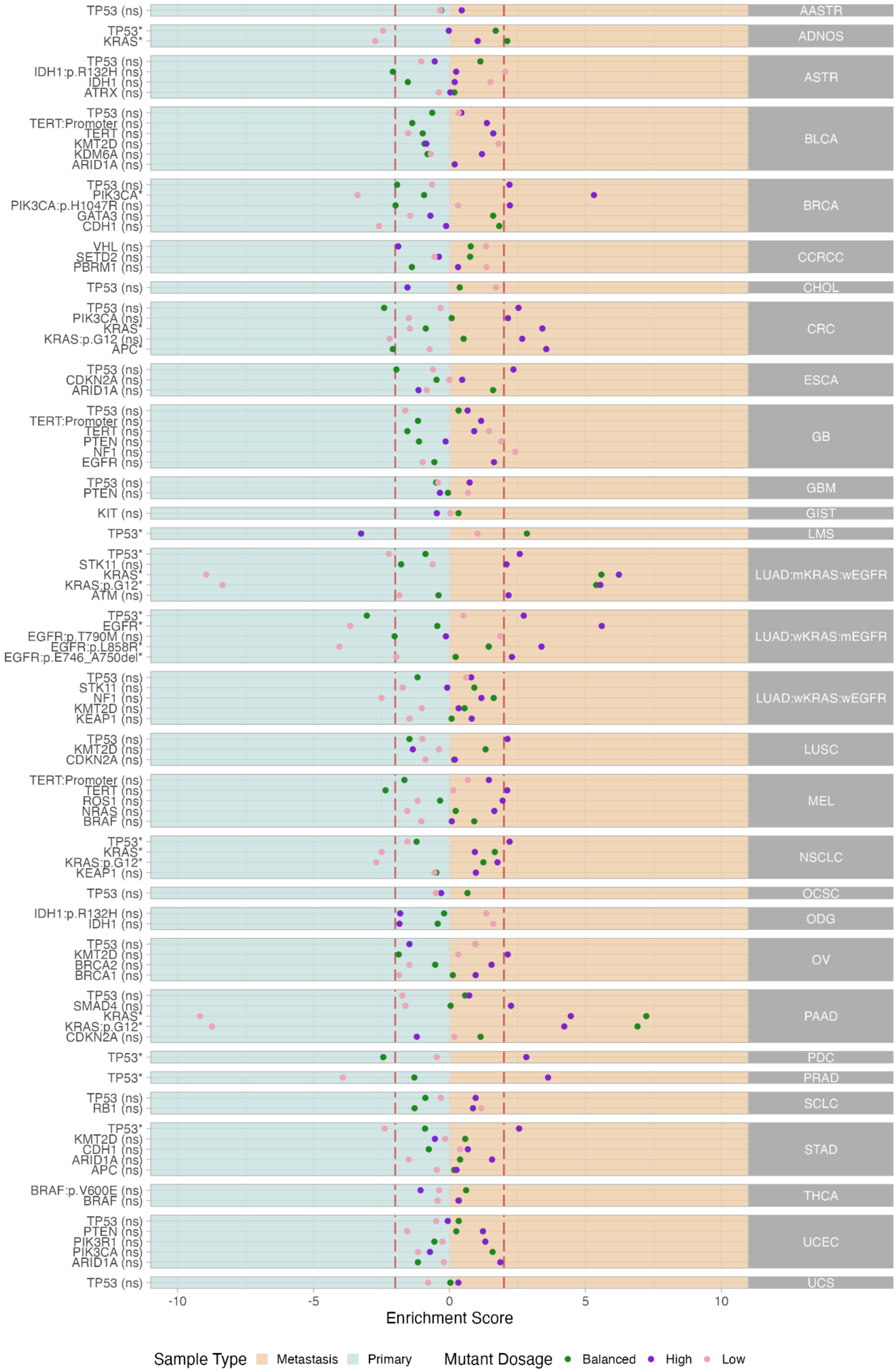

TP53 (ns)
AASTR
TP53*
KRAS*
ADNOS
TP53 (ns)
IDH1:p.R132H (ns)
IDH1 (ns)
ATRX (ns)
ASTR
TP53 (ns)
TERT:Promoter (ns)
TERT (ns)
KMT2D (ns)
KDM6A (ns)
ARID1A (ns)
BLCA
TP53 (ns)
PIK3CA*
PIK3CA:p.H1047R (ns)
GATA3 (ns)
CDH1 (ns)
BRCA
VHL (ns)
SETD2 (ns)
PBRM1 (ns)
CCRCC
TP53 (ns)
CHOL
TP53 (ns)
PIK3CA (ns)
KRAS*
KRAS:p.G12 (ns)
APC*
CRC
TP53 (ns)
CDKN2A (ns)
ARID1A (ns)
ESCA
TP53 (ns)
TERT:Promoter (ns)
TERT (ns)
PTEN (ns)
NF1 (ns)
EGFR (ns)
GB
TP53 (ns)
PTEN (ns)
GBM
KIT (ns)
GIST
TP53*
LMS
TP53*
STK11 (ns)
KRAS*
KRAS:p.G12*
ATM (ns)
LUAD:mKRAS:wEGFR
TP53*
EGFR*
EGFR:p.T790M (ns)
EGFR:p.L858R*
EGFR:p.E746_A750del*
LUAD:wKRAS:mEGFR
TP53 (ns)
STK11 (ns)
NF1 (ns)
KMT2D (ns)
KEAP1 (ns)
LUAD:wKRAS:wEGFR
TP53 (ns)
KMT2D (ns)
CDKN2A (ns)
LUSC
TERT:Promoter (ns)
TERT (ns)
ROS1 (ns)
NRAS (ns)
BRAF (ns)
MEL
TP53*
KRAS*
KRAS:p.G12*
KEAP1 (ns)
NSCLC
TP53 (ns)
OCSC
IDH1:p.R132H (ns)
IDH1 (ns)
ODG
TP53 (ns)
KMT2D (ns)
BRCA2 (ns)
BRCA1 (ns)
OV
TP53 (ns)
SMAD4 (ns)
KRAS*
KRAS:p.G12*
CDKN2A (ns)
PAAD
TP53*
PDC
TP53*
PRAD
TP53 (ns)
RB1 (ns)
SCLC
TP53*
KMT2D (ns)
CDH1 (ns)
ARID1A (ns)
APC (ns)
STAD
BRAF:p.V600E (ns)
BRAF (ns)
THCA
TP53 (ns)
PTEN (ns)
PIK3R1 (ns)
PIK3CA (ns)
ARID1A (ns)
UCEC
TP53 (ns)
UCS
-10
-5
0
5
10
Enrichment Score
Sample Type
Metastasis
Primary
Mutant Dosage
Balanced
High
Low

Supplementary Figure S16: Enrichment scores r in primary tumours vs metastases of low, balanced and high mutant dosage classes, across the most frequently mutated genes per tumour type in the GENIE-DFCI cohort. The enrichment score is evaluated through a two-tailed Fisher's exact test with a significance level α = 0.1 and using standardised residuals. Vertical dashed lines indicate thresholds of large effect size (r = 2).

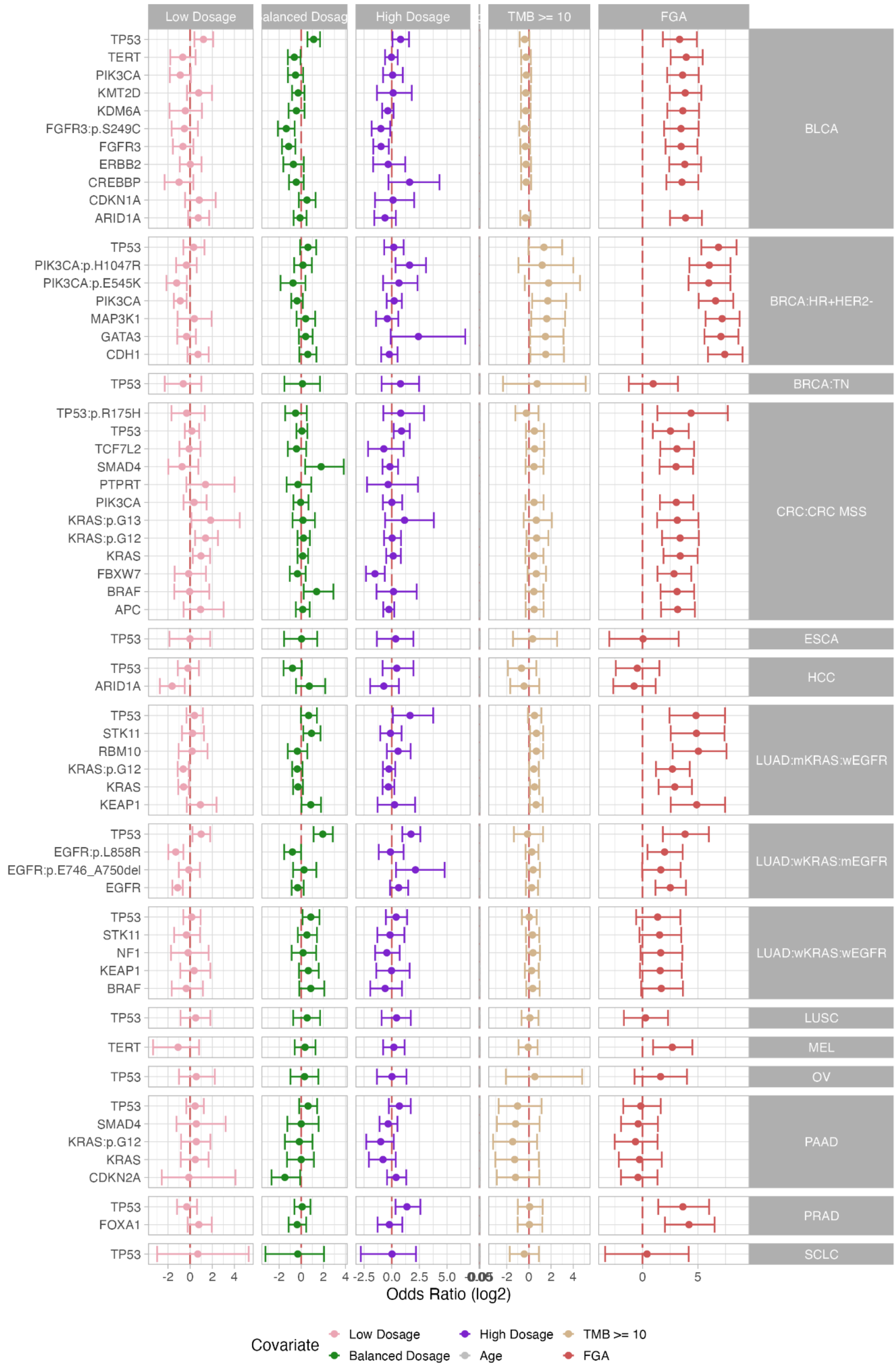

Supplementary Figure S17: Adjusted OR of metastatisation (log2 scale) with 95% confidence interval, from multivariate logistic regression models. Covariates include low, balanced and high mutant dosage classes, age, tumour mutational burden

(TMB) and fragment of genome altered (FGA). Red vertical lines indicate OR = 1. For each tumour type, only the most frequently mutated genes are shown.

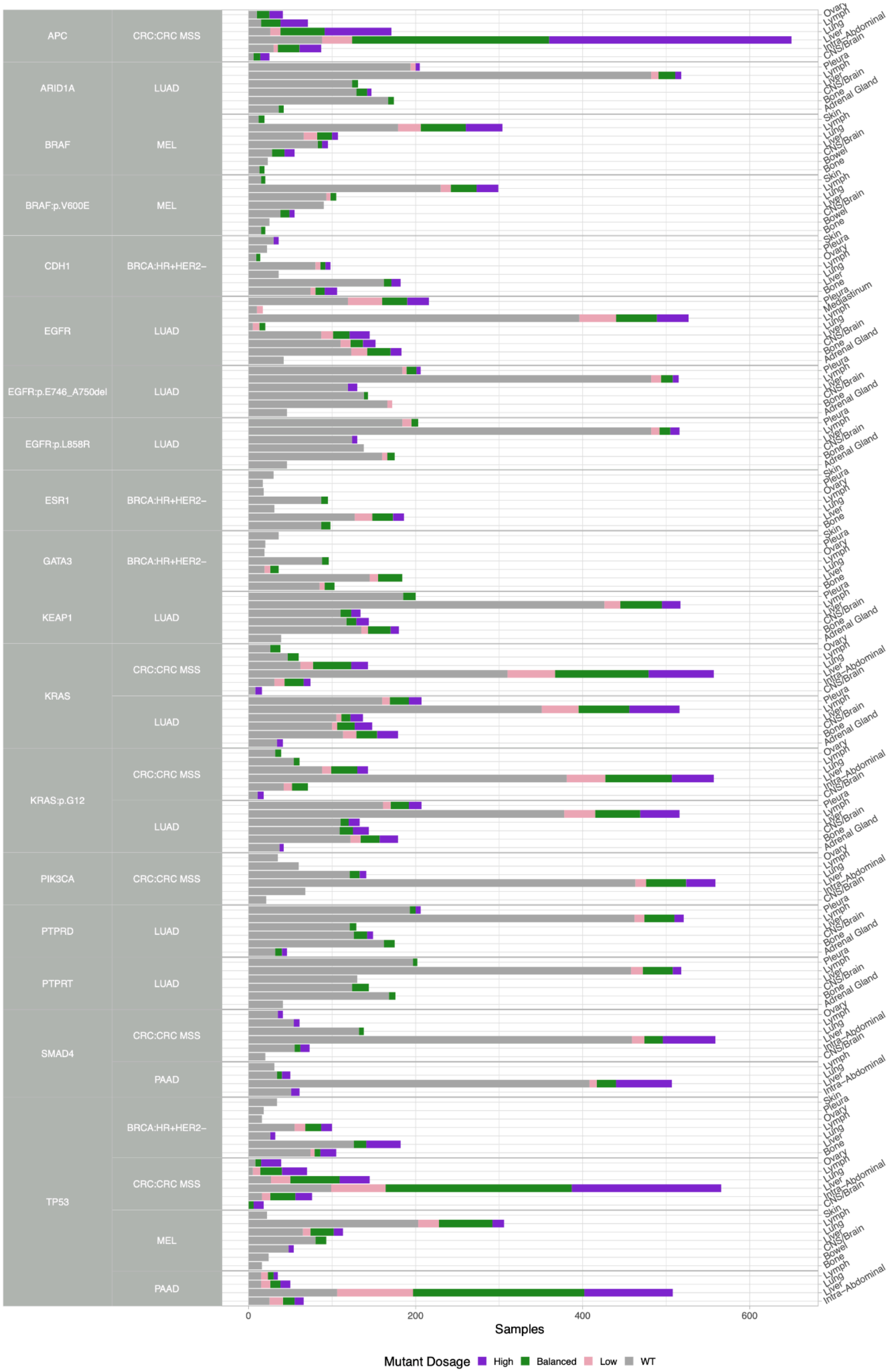

APC
CRC:CRC MSS
ARID1A
LUAD
BRAF
MEL
BRAF:p.V600E
MEL
CDH1
BRCA:HR+HER2−
EGFR
LUAD
EGFR:p.E746_A750del
LUAD
EGFR:p.L858R
LUAD
ESR1
BRCA:HR+HER2−
GATA3
BRCA:HR+HER2−
KEAP1
LUAD
KRAS
CRC:CRC MSS
LUAD
KRAS:p.G12
CRC:CRC MSS
LUAD
PIK3CA
CRC:CRC MSS
PTPRD
LUAD
PTPRT
LUAD
SMAD4
CRC:CRC MSS
PAAD
TP53
BRCA:HR+HER2−
CRC:CRC MSS
MEL
PAAD
0
200
400
600
Samples
Mutant Dosage
High
Balanced
Low
WT

Supplementary Figure S18: Mutant dosage-specific multinomial distribution of metastatic sites per gene and tumour type, comparing organotropic trajectories across wild-type (WT), low, balanced and high mutant dosage classes.

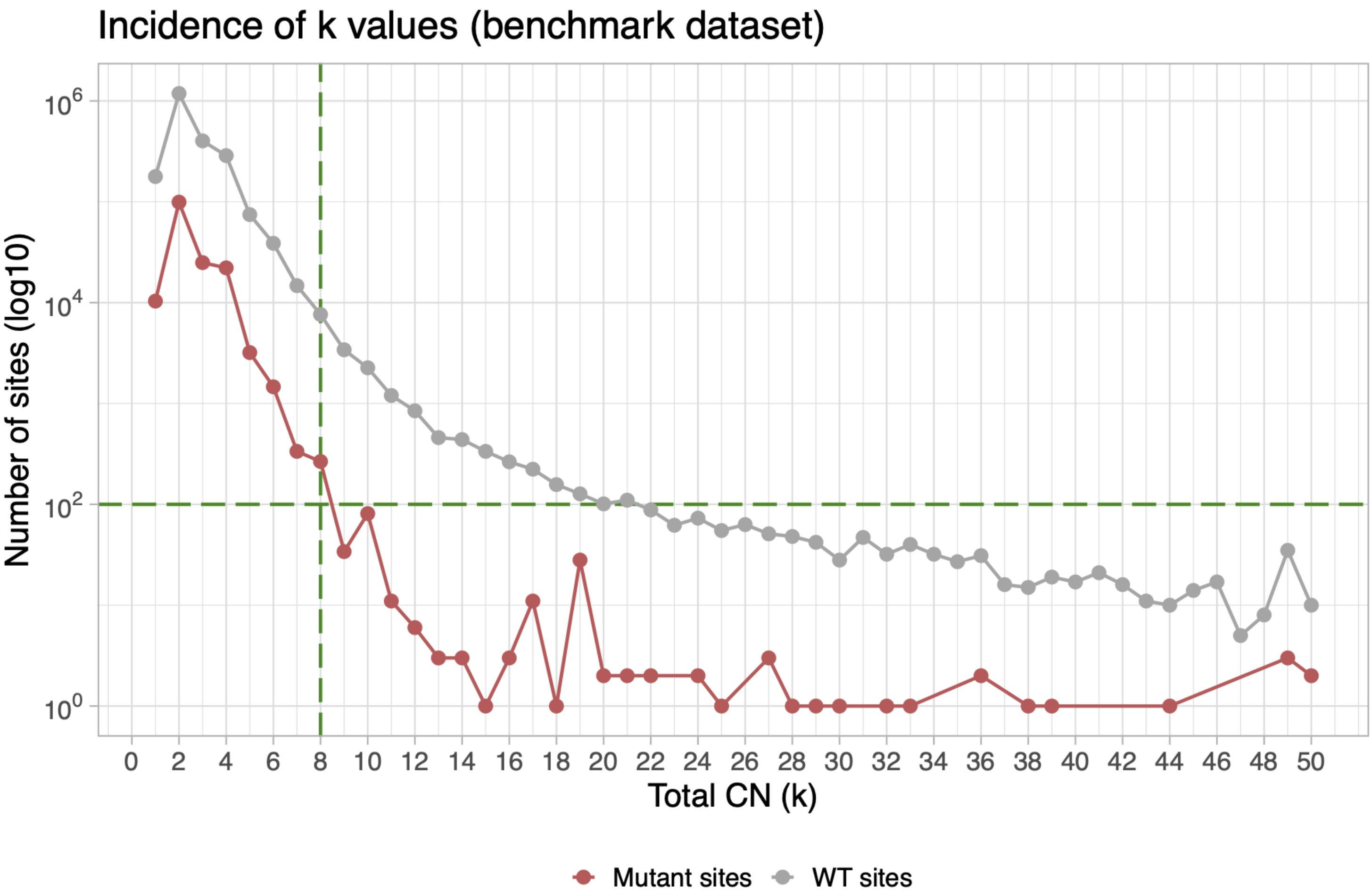

Supplementary Figure S19: Number of mutant (red) and wild-type (grey) sites per total copy number across 8,339 samples from PCAWG, HMF and TCGA. In the analyses reported in this work, we set the maximum total copy number at $kmax$ = 8 to reduce the computational burden. High copy gains were more frequent in wild-type regions, and for copy number values k > $kmax$ we observed a significant drop in the number of mutant sites per copy-number (less than 100).

References

1. Guo, Y. *et al.* Prognostic and clinicopathological value of GATA binding protein 3 in breast cancer: A systematic review and meta-analysis. *PLoS One* **12**, e0174843 (2017).
2. Migliaccio, I. *et al.* PIK3CA co-occurring mutations and copy-number gain in hormone receptor positive and HER2 negative breast cancer. *NPJ Breast Cancer* **8**, 24 (2022).
3. Ciriello, G. *et al.* Comprehensive molecular portraits of invasive lobular breast cancer. *Cell* **163**, 506–519 (2015).
4. Jorissen, R. N. *et al.* Wild-type APC predicts poor prognosis in microsatellite-stable proximal colon cancer. *Br. J. Cancer* **113**, 979–988 (2015).
5. Yokota, T. *et al.* BRAF mutation is a powerful prognostic factor in advanced and recurrent colorectal cancer. *Br. J. Cancer* **104**, 856–862 (2011).
6. Guo, Y. A. *et al.* Mutation hotspots at CTCF binding sites coupled to chromosomal instability in gastrointestinal cancers. *Nat. Commun.* **9**, 1520 (2018).
7. Pileri, P. *et al.* FAT1: a potential target for monoclonal antibody therapy in colon cancer. *Br. J. Cancer* **115**, 40–51 (2016).

8. Nguyen, K. *et al.* MAP3K family review and correlations with patient survival outcomes in various cancer types. *Front. Biosci. (Landmark Ed.)* **27**, 167 (2022).
9. Wasserman, I. *et al.* SMAD4 loss in colorectal cancer patients correlates with recurrence, loss of immune infiltrate, and chemoresistance. *Clin. Cancer Res.* **25**, 1948–1956 (2019).
10. Wenzel, J. *et al.* Loss of the nuclear Wnt pathway effector TCF7L2 promotes migration and invasion of human colorectal cancer cells. *Oncogene* **39**, 3893–3909 (2020).
11. Long, J. *et al.* Development and validation of a TP53-associated immune prognostic model for hepatocellular carcinoma. *EBioMedicine* **42**, 363–374 (2019).
12. Zhou, F. *et al.* The changing treatment landscape of EGFR-mutant non-small-cell lung cancer. *Nat. Rev. Clin. Oncol.* **22**, 95–116 (2025).
13. Scalera, S. *et al.* Clonal KEAP1 mutations with loss of heterozygosity share reduced immunotherapy efficacy and low immune cell infiltration in lung adenocarcinoma. *Ann. Oncol.* **34**, 275–288 (2023).
14. Manolakos, P., Boccuto, L. & Ivankovic, D. S. A critical review of the impact of SMARCA4 mutations on survival outcomes in non-small cell lung cancer. *J. Pers. Med.* **14**, 684 (2024).
15. Willmer, T., Cooper, A., Sims, D., Govender, D. & Prince, S. The T-box transcription factor 3 is a promising biomarker and a key regulator of the oncogenic phenotype of a diverse range of sarcoma subtypes. *Oncogenesis* **5**, e199 (2016).
16. Mueller, S. *et al.* A disease model resource reveals core principles of tissue-specific cancer evolution. *Nature* 1–12 (2026).
17. Mueller, S. *et al.* Evolutionary routes and KRAS dosage define pancreatic cancer phenotypes. *Nature* **554**, 62–68 (2018).
18. Varghese, A. M. *et al.* Clinicogenomic landscape of pancreatic adenocarcinoma identifies KRAS mutant dosage as prognostic of overall survival. *Nat. Med.* **31**, 466–477 (2025).
19. Suryo Rahmanto, Y. *et al.* Inactivation of Arid1a in the endometrium is associated with endometrioid tumorigenesis through transcriptional reprogramming. *Nat. Commun.* **11**, 2717 (2020).
20. Chiang, S. *et al.* BCOR is a robust diagnostic immunohistochemical marker of genetically diverse high-grade endometrial stromal sarcoma, including tumors exhibiting variant morphology. *Mod. Pathol.* **30**, 1251–1261 (2017).
21. Koivu, M. K. A. *et al.* Trans-activating mutations of the pseudokinase ERBB3. *Oncogene* **43**, 2253–2265 (2024).
22. Akiyama-Abe, A. *et al.* Loss of PTEN expression is an independent predictor of favourable survival in endometrial carcinomas. *Br. J. Cancer* **109**, 1703–1710 (2013).
23. Yarchoan, M., Hopkins, A. & Jaffee, E. M. Tumor mutational burden and response rate to PD-1 inhibition. *N. Engl. J. Med.* **377**, 2500–2501 (2017).
24. Strickler, J. H., Hanks, B. A. & Khasraw, M. Tumor mutational burden as a predictor of immunotherapy response: Is more always better? *Clin. Cancer Res.* **27**, 1236–1241 (2021).
25. Aggarwal, C. *et al.* Assessment of tumor mutational burden and outcomes in patients with diverse advanced cancers treated with immunotherapy. *JAMA Netw. Open* **6**, e2311181 (2023).
26. Frankell, A. M. *et al.* The evolution of lung cancer and impact of subclonal selection in TRACERx. *Nature* **616**, 525–533 (2023).
27. Spurr, L. F. & Pitroda, S. P. Exploiting tumor aneuploidy as a biomarker and therapeutic target in patients treated with immune checkpoint blockade. *NPJ Precis Oncol* **8**, 1 (2024).
28. Modrek, B. *et al.* Oncogenic activating mutations are associated with local copy gain. *Mol. Cancer Res.* **7**, 1244–1252 (2009).
29. Chiosea, S. I., Sherer, C. K., Jelic, T. & Dacic, S. KRAS mutant allele-specific imbalance in lung adenocarcinoma. *Mod. Pathol.* **24**, 1571–1577 (2011).
30. Hartman, D. J., Davison, J. M., Foxwell, T. J., Nikiforova, M. N. & Chiosea, S. I. Mutant allele-specific imbalance modulates prognostic impact of KRAS mutations in colorectal adenocarcinoma and is associated with worse overall survival. *Int. J. Cancer* **131**, 1810–1817 (2012).
31. Fung, A. S. *et al.* Prognostic and predictive effect of KRAS gene copy number and mutation status in early stage non-small cell lung cancer patients. *Transl. Lung Cancer Res.* **10**, 826–838 (2021).
32. Punekar, S. R., Velcheti, V., Neel, B. G. & Wong, K.-K. The current state of the art and future trends in RAS-targeted cancer therapies. *Nat. Rev. Clin. Oncol.* **19**, 637–655 (2022).
33. Correia, L. *et al.* Allelic expression imbalance of PIK3CA mutations is frequent in breast cancer and prognostically significant. *NPJ Breast Cancer* **8**, 71 (2022).
34. Rheinbay, E. *et al.* Analyses of non-coding somatic drivers in 2,658 cancer whole genomes. *Nature* **578**, 102–111 (2020).
35. Ricciuti, B. *et al.* Diminished efficacy of programmed death-(ligand)1 inhibition in STK11- and KEAP1-mutant lung adenocarcinoma is affected by KRAS mutation status. *J. Thorac. Oncol.* **17**, 399–410 (2022).
36. Hanel, W. & Moll, U. M. Links between mutant p53 and genomic instability. *J. Cell. Biochem.* **113**, 433–439 (2012).
37. Wang, Y. *et al.* APOBEC mutagenesis is a common process in normal human small intestine. *Nat. Genet.* **55**, 246–254 (2023).
38. Sanz-Garcia, E., Argiles, G., Elez, E. & Tabernero, J. BRAF mutant colorectal cancer: prognosis, treatment, and new perspectives. *Ann. Oncol.* **28**, 2648–2657 (2017).
39. Wang, H., Guo, M., Wei, H. & Chen, Y. Targeting p53 pathways: mechanisms, structures, and advances in therapy.

Signal Transduct. Target. Ther. **8**, 92 (2023).
40. Martínez-Jiménez, F. *et al.* Pan-cancer whole-genome comparison of primary and metastatic solid tumours. *Nature* 1–9 (2023).
41. Priestley, P. *et al.* Pan-cancer whole-genome analyses of metastatic solid tumours. *Nature* **575**, 210–216 (2019).
42. Collins, N. B. *et al.* PI3K activation allows immune evasion by promoting an inhibitory myeloid tumor microenvironment. *J. Immunother. Cancer* **10**, e003402 (2022).
43. Wilson, M. R. *et al.* ARID1A and PI3-kinase pathway mutations in the endometrium drive epithelial transdifferentiation and collective invasion. *Nat. Commun.* **10**, 3554 (2019).
44. Bahar, M. E., Kim, H. J. & Kim, D. R. Targeting the RAS/RAF/MAPK pathway for cancer therapy: from mechanism to clinical studies. *Signal Transduct. Target. Ther.* **8**, 455 (2023).
45. Church, D. N. *et al.* Prognostic significance of POLE proofreading mutations in endometrial cancer. *J. Natl. Cancer Inst.* **107**, 402 (2015).
46. Gerlinger, M. *et al.* Intratumor heterogeneity and branched evolution revealed by multiregion sequencing. *N. Engl. J. Med.* **366**, 883–892 (2012).
47. Zucker, M. *et al.* Pan-cancer analysis of biallelic inactivation in tumor suppressor genes identifies KEAP1 zygosity as a predictive biomarker in lung cancer. *Cell* **188**, 851–867.e17 (2025).
48. George, J. *et al.* Comprehensive genomic profiles of small cell lung cancer. *Nature* **524**, 47–53 (2015).
49. Hayward, N. K. *et al.* Whole-genome landscapes of major melanoma subtypes. *Nature* **545**, 175–180 (2017).
50. Hu, Q. *et al.* ZFHX3 acts as a tumor suppressor in prostate cancer by targeting FTO-mediated m6A demethylation. *Cell Death Discov.* **10**, 284 (2024).
51. Nakayama, M. *et al.* Loss of wild-type p53 promotes mutant p53-driven metastasis through acquisition of survival and tumor-initiating properties. *Nat. Commun.* **11**, 2333 (2020).
52. Mosele, F. *et al.* Outcome and molecular landscape of patients with PIK3CA-mutated metastatic breast cancer. *Ann. Oncol.* **31**, 377–386 (2020).
53. Oakley, G. J., 3rd & Chiosea, S. I. Higher dosage of the epidermal growth factor receptor mutant allele in lung adenocarcinoma correlates with younger age, stage IV at presentation, and poorer survival. *J. Thorac. Oncol.* **6**, 1407–1412 (2011).
54. Malapelle, U. *et al.* EGFR mutant allelic-specific imbalance assessment in routine samples of non-small cell lung cancer. *J. Clin. Pathol.* **68**, 739–741 (2015).
55. Richart, L. *et al.* STAG2 loss-of-function affects short-range genomic contacts and modulates the basal-luminal transcriptional program of bladder cancer cells. *Nucleic Acids Res.* **49**, 11005–11021 (2021).
56. Robinson, B. D. *et al.* Upper tract urothelial carcinoma has a luminal-papillary T-cell depleted contexture and activated FGFR3 signaling. *Nat. Commun.* **10**, 2977 (2019).
57. Leyderman, M. *et al.* Metastasis development in non-muscle-invasive bladder cancer. *Nat. Rev. Urol.* **22**, 375–386 (2024).
58. Li, D. *et al.* FBXW7 and its downstream NOTCH pathway could be potential indicators of organ-free metastasis in colorectal cancer. *Front. Oncol.* **11**, 783564 (2021).
59. Bredin, H. K., Krakstad, C. & Hoivik, E. A. PIK3CA mutations and their impact on survival outcomes of patients with endometrial cancer: A systematic review and meta-analysis. *PLoS One* **18**, e0283203 (2023).
60. Nguyen, B. *et al.* Genomic characterization of metastatic patterns from prospective clinical sequencing of 25,000 patients. *Cell* **185**, 563–575.e11 (2022).
61. Golas, M. M. *et al.* Cytogenetic signatures favoring metastatic organotropism in colorectal cancer. *Nat. Commun.* **16**, 3261 (2025).
62. Sharie, A. H. A. *et al.* Lung adenocarcinoma with bone metastases: Clinicogenomic profiling and insights into prognostic factors. *Cancer Control* **32**, 10732748251325587 (2025).
