# Supplementary material for: Gene mutant dosage is associated with prognosis and metastatic tropism in 60,000 clinical cancer samples
Source: Nat Genet. 2026 Jul 31;58(8):1906–17. doi: 10.1038/s41588-026-02666-z (PMC13447112; doi:10.1038/s41588-026-02666-z)
Supplement: Supplementary file 2 [file 41588_2026_2666_MOESM2_ESM.pdf]

nature portfolio

Corresponding author(s): Giulio Caravagna

Last updated by author(s): 2026/04/28

Reporting Summary

Nature Portfolio wishes to improve the reproducibility of the work that we publish. This form provides structure for consistency and transparency in reporting. For further information on Nature Portfolio policies, see our Editorial Policies and the Editorial Policy Checklist.

Statistics

For all statistical analyses, confirm that the following items are present in the figure legend, table legend, main text, or Methods section.

n/a	Confirmed	
☐	☒	The exact sample size (*n*) for each experimental group/condition, given as a discrete number and unit of measurement
☐	☒	A statement on whether measurements were taken from distinct samples or whether the same sample was measured repeatedly
☐	☒	The statistical test(s) used AND whether they are one- or two-sided<br>*Only common tests should be described solely by name; describe more complex techniques in the Methods section.*
☐	☒	A description of all covariates tested
☐	☒	A description of any assumptions or corrections, such as tests of normality and adjustment for multiple comparisons
☐	☒	A full description of the statistical parameters including central tendency (e.g. means) or other basic estimates (e.g. regression coefficient) AND variation (e.g. standard deviation) or associated estimates of uncertainty (e.g. confidence intervals)
☐	☒	For null hypothesis testing, the test statistic (e.g. *F*, *t*, *r*) with confidence intervals, effect sizes, degrees of freedom and *P* value noted<br>*Give P values as exact values whenever suitable.*
☐	☒	For Bayesian analysis, information on the choice of priors and Markov chain Monte Carlo settings
☐	☒	For hierarchical and complex designs, identification of the appropriate level for tests and full reporting of outcomes
☐	☒	Estimates of effect sizes (e.g. Cohen's *d*, Pearson's *r*), indicating how they were calculated

Our web collection on statistics for biologists contains articles on many of the points above.

Software and code

Policy information about availability of computer code

Data collection	All the relevant data analysed in this work was obtained from public data resources with no use of software.
Data analysis	All the analyses were carried out using the INCOMMON R package (v1.0, https://github.com/caravagnalab/INCOMMON). Statistical analyses were conducted in R version 4.3.2 using the following packages: tidyverse (v2.0.0), stats (v4.3.3), survival (v3.7-0), survminer (v0.4.9), multcomp (v1.4-26), and nnet (v7.3-20)

For manuscripts utilizing custom algorithms or software that are central to the research but not yet described in published literature, software must be made available to editors and reviewers. We strongly encourage code deposition in a community repository (e.g. GitHub). See the Nature Portfolio guidelines for submitting code & software for further information.

Data

Policy information about availability of data

All manuscripts must include a data availability statement. This statement should provide the following information, where applicable:

- Accession codes, unique identifiers, or web links for publicly available datasets
- A description of any restrictions on data availability
- For clinical datasets or third party data, please ensure that the statement adheres to our policy

All analyses presented in this paper, from data gathering to analysis results, are available in Zenodo at https://zenodo.org/uploads/15498338. PCAWG and TCGA data used in this article (mutations with validated matched CNAs) are available from Zenodo at https://doi.org/10.5281/zenodo.6410935.The MSK MetTropism

data used in this article have been downloaded from the CBioPortal at https://www.cbioportal.org, under cohort "msk_met_2021". AACR GENIE-DFCI data has been downloaded from Synapse at https://www.synapse.org/ through access codes syn50678641, syn50678411, syn50678410, syn50678644, syn50678531, syn50678642, syn50678530, syn50678532, syn50678295, syn50678640, syn50678653, syn50678296. HMF data (segmentation files and purity/ploidy estimates for ? = 4,496 samples) were obtained from the Hartwig Medical Foundation (version 17/10/2020), using the request forms that can be found at www.hartwigmedicalfoundation.nl. The files were generated using the PURPLE pipeline, available at github.com/hartwigmedical/hmftools/. We then manually converted the Hartwig annotations to match those in ICGC (Supplementary Table S9). INCOMMON is an open-source R package downloadable from Github at https://caravagnalab.github.io/INCOMMON. The tool webpage contains RMarkdown vignettes to run analyses, visualise inputs and outputs, and parametrise the tool. INCOMMON is also available as a ShinyApp that can be used to browse analysis results and run online similar analyses. The app is available online at https://ncalonaci.shinyapps.io/incommon/.

Research involving human participants, their data, or biological material

Policy information about studies with human participants or human data. See also policy information about sex, gender (identity/presentation), and sexual orientation and race, ethnicity and racism.

Reporting on sex and gender

Use the terms sex (biological attribute) and gender (shaped by social and cultural circumstances) carefully in order to avoid confusing both terms. Indicate if findings apply to only one sex or gender; describe whether sex and gender were considered in study design; whether sex and/or gender was determined based on self-reporting or assigned and methods used.
Provide in the source data disaggregated sex and gender data, where this information has been collected, and if consent has been obtained for sharing of individual-level data; provide overall numbers in this Reporting Summary. Please state if this information has not been collected.
Report sex- and gender-based analyses where performed, justify reasons for lack of sex- and gender-based analysis.

Reporting on race, ethnicity, or other socially relevant groupings

Please specify the socially constructed or socially relevant categorization variable(s) used in your manuscript and explain why they were used. Please note that such variables should not be used as proxies for other socially constructed/relevant variables (for example, race or ethnicity should not be used as a proxy for socioeconomic status).
Provide clear definitions of the relevant terms used, how they were provided (by the participants/respondents, the researchers, or third parties), and the method(s) used to classify people into the different categories (e.g. self-report, census or administrative data, social media data, etc.)
Please provide details about how you controlled for confounding variables in your analyses.

Population characteristics

Describe the covariate-relevant population characteristics of the human research participants (e.g. age, genotypic information, past and current diagnosis and treatment categories). If you filled out the behavioural & social sciences study design questions and have nothing to add here, write "See above."

Recruitment

Describe how participants were recruited. Outline any potential self-selection bias or other biases that may be present and how these are likely to impact results.

Ethics oversight

Identify the organization(s) that approved the study protocol.

Note that full information on the approval of the study protocol must also be provided in the manuscript.

Field-specific reporting

Please select the one below that is the best fit for your research. If you are not sure, read the appropriate sections before making your selection.

☒ Life sciences ☐ Behavioural & social sciences ☐ Ecological, evolutionary & environmental sciences

For a reference copy of the document with all sections, see nature.com/documents/nr-reporting-summary-flat.pdf

Life sciences study design

All studies must disclose on these points even when the disclosure is negative.

Sample size

Sample size was not predetermined by statistical power calculations but was defined by the number of patients included in the original dataset. No new data were generated. The number of datasets included in this study was determined to enable (i) robust estimation of empirical prior distributions and (ii) sufficiently powered discovery and validation of associations with clinical outcomes.
Empirical priors were derived from a benchmark cohort assembled from high-resolution whole-genome sequencing data from the Pan-Cancer Analysis of Whole Genomes (N = 2,777) and the Hartwig Medical Foundation (N = 8,616), providing large sample size, tumour-type diversity, and representation of both primary and metastatic disease for stable estimation of copy number configurations.
The method was applied to the MSK-MET cohort (N = 21,937 tumours) to identify associations between mutant dosage and overall survival, as well as differences between primary (N = 13,216) and metastatic (N = 8,721) tumours, enabling the discovery of biomarkers related to prognosis, metastatic propensity, and tropism. These findings were independently validated in the AACR Project GENIE dataset (N = 33,511 tumours).
These datasets provide sufficient statistical power, clinical heterogeneity, and independent replication to support robust and generalizable identification of clinically relevant biomarkers.

Data exclusions

Samples were excluded if they lacked essential data required for the analysis (e.g., missing purity estimates, sequencing data, or clinical annotations). Specific exclusion criteria and resulting sample counts are described in the Methods and/or figure legends. All exclusions were made prior to analysis and were not outcome-driven.

Replication	This was a retrospective study using existing clinical and genomic data. No experimental replication was applicable. Where possible, robustness of findings was assessed through internal validation. All analysis code and parameters are provided for reproducibility.
Randomization	Randomization was not applicable, as this study did not involve experimental interventions. All analyses were observational and based on pre-existing clinical and sequencing data from the MSK-MET cohort.
Blinding	Blinding was not applicable, as no treatment allocation or outcome assessment was performed in this retrospective analysis.

Reporting for specific materials, systems and methods

We require information from authors about some types of materials, experimental systems and methods used in many studies. Here, indicate whether each material, system or method listed is relevant to your study. If you are not sure if a list item applies to your research, read the appropriate section before selecting a response.

Materials & experimental systems

n/a	Involved in the study
☒	☐ Antibodies
☒	☐ Eukaryotic cell lines
☒	☐ Palaeontology and archaeology
☒	☐ Animals and other organisms
☒	☐ Clinical data
☒	☐ Dual use research of concern
☒	☐ Plants

Methods

n/a	Involved in the study
☒	☐ ChIP-seq
☒	☐ Flow cytometry
☒	☐ MRI-based neuroimaging

Plants

Seed stocks	*Report on the source of all seed stocks or other plant material used. If applicable, state the seed stock centre and catalogue number. If plant specimens were collected from the field, describe the collection location, date and sampling procedures.*
Novel plant genotypes	*Describe the methods by which all novel plant genotypes were produced. This includes those generated by transgenic approaches, gene editing, chemical/radiation-based mutagenesis and hybridization. For transgenic lines, describe the transformation method, the number of independent lines analyzed and the generation upon which experiments were performed. For gene-edited lines, describe the editor used, the endogenous sequence targeted for editing, the targeting guide RNA sequence (if applicable) and how the editor was applied.*
Authentication	*Describe any authentication procedures for each seed stock used or novel genotype generated. Describe any experiments used to assess the effect of a mutation and, where applicable, how potential secondary effects (e.g. second site T-DNA insertions, mosiacism, off-target gene editing) were examined.*
